# Domain architecture of plant eukaryotic translation initiation factor 3 subunit E governs interaction with translational *cis*-elements to regulate pollen tube growth

**DOI:** 10.1093/plcell/koag005

**Published:** 2026-02-17

**Authors:** Vinod Kumar, Rémy Merret, Marie-Christine Carpentier, David Honys, Said Hafidh

**Affiliations:** Laboratory of Pollen Biology, Institute of Experimental Botany of the Czech Academy of Sciences, Prague 6 165 00, Czech Republic; Department of Experimental Plant Biology, Faculty of Science, Charles University, Praha 2 128 44, Czech Republic; Institut de biologie moléculaire des plantes, CNRS, Université de Strasbourg, Strasbourg, France; Laboratoire Génome et Développement des Plantes, UMR5096, CNRS, Perpignan 66860, France; Laboratory of Pollen Biology, Institute of Experimental Botany of the Czech Academy of Sciences, Prague 6 165 00, Czech Republic; Laboratory of Pollen Biology, Institute of Experimental Botany of the Czech Academy of Sciences, Prague 6 165 00, Czech Republic

## Abstract

An octameric eukaryotic translation initiation factor 3 subunit E (eIF3E) preserves translational homeostasis through selective messenger RNA (mRNA) recognition and ribosome assembly. Yet, the mechanisms by which eIF3E maintains translational equilibrium remain poorly understood. We show here that eIF3E domain architecture and phosphorylation sites (Thr417, Ser421) are conserved across eukaryotes. Deleting the Proteasome-COP9 signalosome-Initiation factor 3 domain (PCI domain) abolished nuclear localization, disrupted eIF3E–eIF3L interaction, and impaired eIF3E dissociation from the polysomes. Affnity RNA immunoprecipitation sequencing of eIF3E::YFP in tobacco pollen tubes identified mRNAs bearing coding-sequence motifs (MC1 to MC3) that co-immunoprecipitate with eIF3E. Using mRNA reporter assay, we reveal that these motifs act in tandem as eIF3E-dependent translational repressors and enhancers. AlphaFold3 structural modeling and Förster resonance energy transfer verification indicate that PCI domain deletion or PCI-phosphosite mutagenesis weaken eIF3E–eIF3L interactions and block translational activation of MC2 RNA reporter. We further show that loss of the PCI domain or PCI-phosphosite mutagenesis misregulate pollen tube growth and membrane organization. Together, our findings underscore eIF3E as a selective regulator of mRNA translation that couples *cis*-motif recognition to membrane integrity and pollen tube growth, thereby ensuring plant fertility.

## Teaser

Translation initiation subunit eIF3E helps balance protein production by coordinating opposing genetic *cis*-elements, affecting pollen tube growth and the shape of cell membranes.

## Graphical Abstract

**Graphical Abstract:**
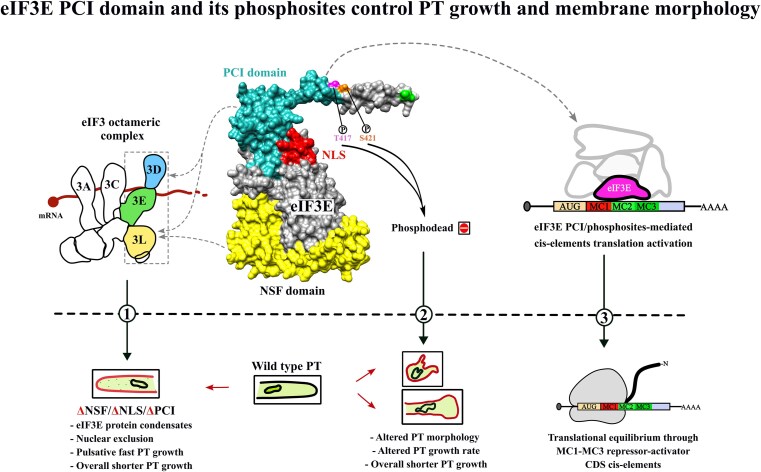

## Introduction

The translation of messenger RNA (mRNA) is a critical determinant of gene expression, influencing plant growth, development, and responses to environmental stimuli (Wu et al. 2024; Yuan et al. 2024). Translation is primarily regulated at the initiation phase, which is orchestrated by eukaryotic translation initiation factors (eIFs) (Browning et al. 2001; Brito Querido et al. 2024; Kapp and Lorsch 2004). Among these, eIF3 is the largest and most complex initiation factor, consisting of 13 non-identical subunits (eIF3A–M) in animals and playing a central role in assembling the pre-initiation complex (Asano et al. 1997; Burks et al. 2001; Dong and Zhang 2006; Hinnebusch 2006; Sha et al. 2009; Brito Querido et al. 2024).

In budding yeast (*Saccharomyces cerevisiae*), eIF3 forms a smaller complex composed of 5 stoichiometric subunits orthologs of human eIF3A, -3B, -3C, -3G, and -3I, and 1 substoichiometric subunit, eIF3J, which together form the core complex essential for translation initiation (Asano et al. 1997; Hinnebusch 2006; Valášek et al. 2017; Raabe et al. 2019; Herrmannová et al. 2024). In *Schizosaccharomyces pombe*, 2 distinct eIF3 assemblies exist: 1 containing eIF3M (eIF3A, -3B, -3C, -3F, -3G, -3H, -3I, -3M) and another containing eIF3E (eIF3A, -3B, -3C, -3D, -3E, -3F, -3G, -3I), both sharing a conserved core (Akiyoshi et al. 2001; Dunand-Sauthier et al. 2002; Zhou et al. 2005; Ray et al. 2008). In mammals, a minimal octameric core (eIF3A, -3C, -3E, -3F, -3H, -3K, -3L, -3M) forms through C-terminal Proteasome-COP9 signalosome-Initiation factor 3 (PCI) domain interactions (Smith et al. 2016), while eIF3J connects the yeast-like core subcomplex (eIF3B, -3G, -3I) to the octamer via eIF3A (Hinnebusch 2006). The peripheral subunit eIF3D is recruited through its association with eIF3E (Valášek et al. 2017).

In plants such as *Arabidopsis thaliana*, *Triticum aestivum*, and *Oryza sativa*, eIF3 comprises 12 subunits, reflecting a structural organization similar to that of mammals (Browning et al. 2001; Li et al. 2016; Raabe et al. 2019). Several plant eIF3 subunits have acquired specialized regulatory functions across developmental stages and tissues. The major subunit eIF3A, together with eIF3B and eIF3C, interacts with the reinitiation-supporting protein to promote reinitiation on long viral mRNAs such as those of Cauliflower mosaic virus (Thiébeauld et al. 2009). Mutation of eIF3C results in embryonic defects and seed abortion (Roy et al. 2010), while both eIF3B and eIF3C undergo light-dependent phosphorylation (Boex-Fontvieille et al. 2013). The peripheral subunit eIF3D is expressed during microgametogenesis, although no coding-sequence mutation with a discernible phenotype has been identified (Roy et al. 2010).

The eIF3E subunit, well-characterized in both plants and animals, is essential for sporophytic and gametophytic development. In Arabidopsis, null Ateif3e mutants exhibit embryo lethality and pollen germination defects (Yahalom et al. 2008; Roy et al. 2010, 2011). eIF3E also interacts with the 26S proteasome and the COP9 signalosome also through the PCI domain (Karniol et al. 1998; Pick et al. 2009; Smith et al. 2016), and *eif3e* and csn mutants display similar phenotypes, suggesting that eIF3E may be regulated via COP9 signalosome-mediated degradation (Yahalom et al. 2008). Additionally, eIF3E facilitates the recruitment of eIF4G to the pre-initiation complex (Yahalom et al. 2008). In rice, OseIF3E regulates seedling development, pollen maturation, and yield-related traits through interactions with OsICK kinases (Wang et al. 2016). Overexpression of AteIF3E reduces translation activity *in vitro* by competitively binding the 40S ribosomal subunit, thereby impeding eIF3 complex association (Paz-Aviram et al. 2008). Moreover, AteIF3F and AteIF3H, both interacting with eIF3E, are critical for pollen germination, pollen tube growth, and embryo development (Xia et al. 2010; Roy et al. 2011). Similar roles are observed for OseIF3F and OseIF3H in rice, where mutations cause male gametophyte arrest and fertility defects (Kim et al. 2004; Li et al. 2016). Recently, AteIF3M2 was shown to enhance pollen tube thermotolerance by reducing reactive oxygen species accumulation (Kahrizi et al. 2025). Together, these findings highlight the essential and diverse functions of eIF3 subunits in plant development and fertility.

During translation initiation, eIF3 prevents premature joining of the 60S and 40S ribosomal subunits, while recruiting the ternary complex (eIF2–GTP–Met–tRNAᵢᴹᵉᵗ) to form the 43S pre-initiation complex (Chaudhuri et al. 1999; Sonenberg and Hinnebusch 2009). eIF3 also aids mRNA recruitment through RNA recognition motifs (RRMs) present in octameric subunits such as eIF3B and eIF3G, and through interactions with the cap-binding eIF4F complex. Within eIF3, the eIF3B C-terminal PCI domain mediates binding to eIF3G and eIF3I, while its N-terminal RRM interacts with eIF3A and eIF3J (Dong et al. 2013). Deletion of the eIF3B RRM disrupts complex integrity and impairs 40S binding. eIF3 cooperates with eIF4E and eIF4G to form the 48S pre-initiation complex, positioning the ribosome at the mRNA 5′ cap (Kolupaeva et al. 2005; Hinnebusch 2006; Smith et al. 2016). eIF4E also associates with the poly(A)-binding protein (PABP) to circularize mRNA and enhance translational efficiency (Brito Querido et al. 2024). Recent studies suggest that eIF3E directly interacts with eIF4G within the 43S complex, further supporting its key role in cap-dependent translation initiation (Chiluiza et al. 2011). Upon start codon recognition, GTP hydrolysis by eIF5 triggers dissociation of most eIF3 subunits, allowing elongation factors eEF1A and eEF2 to join the ribosome for active translation (Hinnebusch and Lorsch 2012; Valášek et al. 2017).

Beyond its canonical role, eIF3E has been implicated in selective mRNA translation. In fission yeast, eIF3E associates with mRNAs encoding metabolic enzymes, transcription factors, and transporters essential for viability (Zhou et al. 2005). Depletion of eIF3E or eIF3D disrupts translation of mitochondrial mRNAs, leading to oxidative stress and premature aging (Shah et al. 2016). In Drosophila, eIF3E interacts with eIF3C to regulate patched1 (Ptch1) mRNA translation, a key developmental regulator (Fujii et al. 2021). Although eIF3E lacks a canonical RRM, it likely associates with specific transcripts through interactions with RRM-containing eIF3 subunits and translation regulators such as eIF4G and PABPs (Gorgoni and Gray 2004).

To dissect the molecular basis of eIF3E function in plant reproductive development (Hafidh and Honys 2021), we combined in silico structural predictions with *in vivo* interaction and functional assays. We performed RNA immunoprecipitation sequencing (RIP-seq) following AteIF3E::YFP expression in tobacco pollen tubes. Our analysis revealed that eIF3E co-purifies with mRNAs involved in pollen tube tip growth, energy metabolism, and cell wall remodeling. Using AlphaFold3-guided modeling, co-immunoprecipitation (co-IP), and proteomic analyses in Arabidopsis and Nicotiana pollen systems, we mapped the eIF3E interactome and identified its conserved associations within the eIF3 octameric core. Furthermore, domain-deletion and ribosome-fractionation analyses revealed the essential role of the C-terminal PCI domain in eIF3E dissociation and translational efficiency. Together, these complementary approaches uncover how eIF3E coordinates translation initiation and energy-regulated growth during pollen tube development and identifies eIF3E as a central regulator of translational activation and pollen tube morphogenesis.

## Results

### eIF3E exhibits conserved domain architecture and functional redundancy

**Figure 1:**
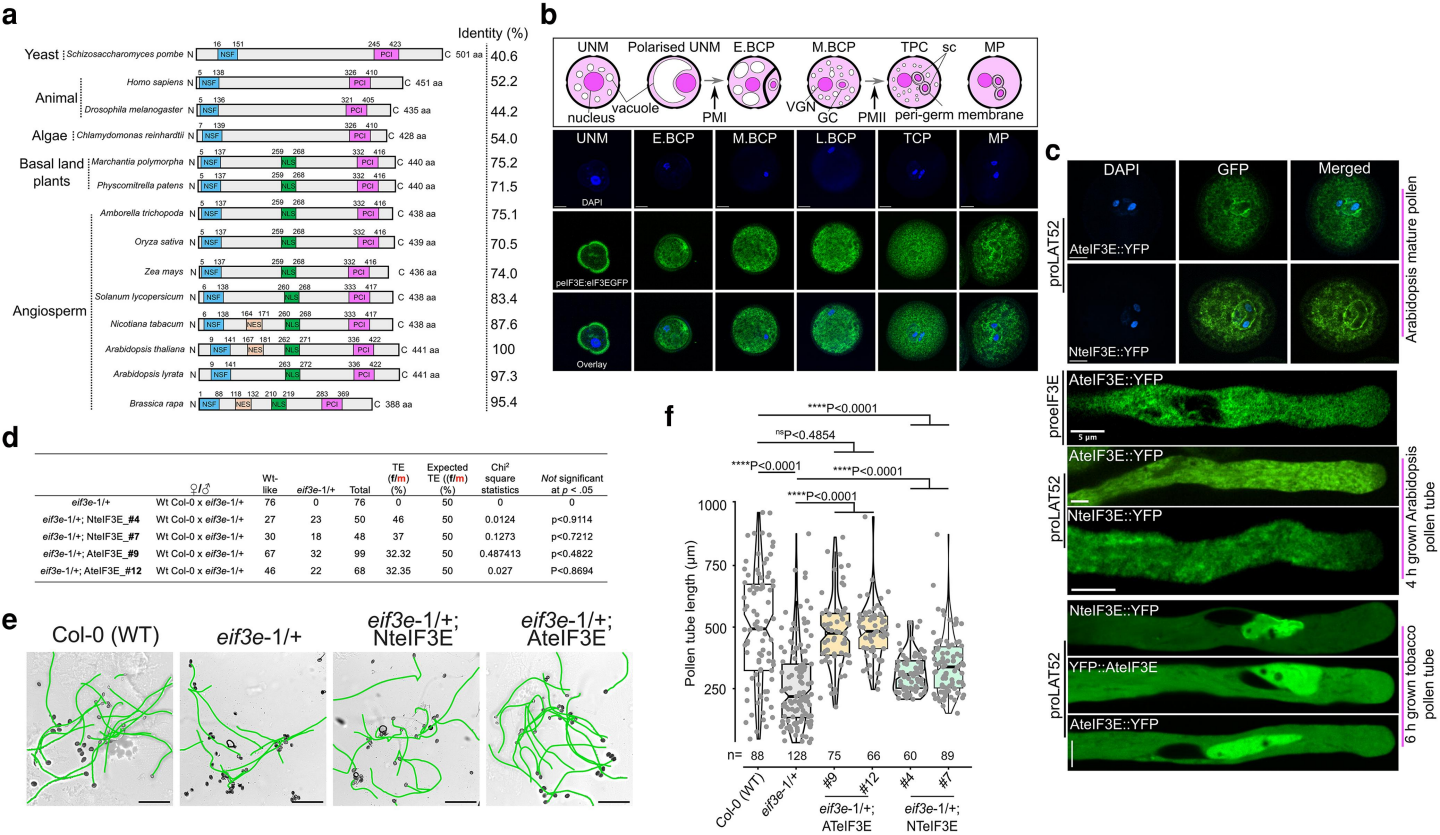
Comparative phylogenetic, localization and functional analysis of eIF3E. (a) Domain architecture of the eIF3E subunit from Archaeplastida, yeast, human, and *Drosophila*. NSF and PCI domains were predicted by InterPro, Expasy-PROSITE, SMART, and EMBL databases. NLS were predicted with NLS Mapper, while NES were predicted by LocNES or manually curated based on homology alignments. (b) Pollen developmental stages from anthers expressing AteIF3E under its native promoter, tagged with YFP (peIF3E:AteIF3E::YFP), counterstained with 4′,6-diamidine-2′-phenylindole dihydrochloride. High-resolution confocal imaging with the Airyscan module reveals eIF3E localization across developmental stages: uninuclear microspore (UNM), early bicellular (E-BCP), late bicellular (L-BCP), tricellular (TCP), and mature pollen (MP). A punctate distribution surrounds the peri-germ cell membrane of the male germ unit (MGU) at the MP stage. Illustrations depict corresponding developmental stages. Arrows indicate pollen mitosis I (PMI) and pollen mitosis II (PMII). Scale bar = 10 *μ*m. (c) Confocal images of pollen and pollen tubes expressing AteIF3E or NteIF3E fused to N- or C-terminal YFP under the LAT52 promoter. Both proteins localize around the MGU with granular cytoplasmic signals in mature pollen and show uniform distribution in in vitro–grown pollen tubes of stable *Arabidopsis* transgenic lines. Comparative localization of tobacco NteIF3E::YFP and *Arabidopsis* eIF3E::YFP (N- or C-terminal fusions) was further assessed in transiently transformed tobacco pollen tubes. (d) Male transmission efficiency (TE) of the *eif3e-1* mutant following complementation with NteIF3E or AteIF3E expressed under the LAT52 promoter. TE was calculated as [TE = (Wt/Het × 100)]. Statistical significance was assessed by *χ*^2^ test (*P* < 0.01). (e, f) Complementation of the *Arabidopsis eif3e-1* mutant pollen tube germination defect. Both NteIF3E::YFP and AteIF3E::YFP fully rescued the phenotype. Notch-boxplot embedded in violin plot prepared in R studio, where center line represent the median and the first and third quartiles indicate 25th and 75th percentiles, and the whiskers extend from minimum to maximum with 1.5 times the interquartile range from the 25th and 75th percentiles. All the data points shown as gray dots indicates an individual pollen tube length. Statistical comparisons was performed with unpaired nonparametric *t*-test using the Mann–Whitney *U* test (GraphPad Prism 9.1.1). ^#^Number of independent lines evaluated. Sample size range from *n* = 60 to 128 (at least 3 replicates were performed for each individual data set). Scale bar = 200 *μ*m applied to all panel images.

To define the conserved essential features of eIF3E in eukaryotes, phylogenetic analysis was performed using 65 diverse sequenced genomes from Archaeplastida to explore eIF3E structural conservation with closely related homologs (Figs. 1aand S1a). The dataset included representatives from Rhodophyta and Chlorophytes, such as *Porphyra umbilicalis*, *Botryococcus braunii*, *Chlamydomonas reinhardtii*, *Dunaliella salina*, *Micromonas pusilla*, and *Volvox carteri*. Additionally, we have incorporated bryophytes (*Physcomitrium patens*), peat moss (*Sphagnum fallax*), liverwort (*Marchantia polymorpha*), lycophyte (*Selaginella moellendorffii*), basal angiosperm (*Amborella trichopoda*), 11 monocots, and 41 dicots. To provide a broader evolutionary context, we have also included yeast (*S. pombe*), fruitfly (*Drosophila melanogaster*) and humans (*Homo sapiens*). Phylogenetic reconstruction revealed that eIF3E homologs clustered into 5 main groups. Notably, *A. trichopoda*, the most basal angiosperm, grouped with monocots and was phylogenetically closer to liverwort and moss while diverged from dicots (Figs. 1a and S1a). This suggests that eIF3E amino acid diversification was not significantly influenced by the evolution of flowering plants or vascularization. Structurally, we identified 4 conserved features in eIF3E; an N-terminal superfamily (NSF) domain, a non-canonical nuclear export signal (NES) with a 2-3-1 pattern (*Φ1XXΦ2XXXΦ3XΦ4*, where *Φ* represents Leu, Val, Ile, Phe, or Met, and *X* represents any amino acid, Fung et al. 2021), a centrally located nuclear localization signal (NLS), and a C-terminal PCI domain (Fig. 1a). The PCI domain is known to mediate interactions with ribosomal proteins, other subunits of the eIF3 octameric complex, COP9 signalosomes, and the 19S regulatory lid subunit of the proteasome complex (Yahalom et al. 2008; Sesen et al. 2017). These domains appear generally conserved across eukaryotes with exception of the NLS motif that was notably absent in green algae, humans, *Drosophila*, and yeast (Fig. 1a). Additionally, computational predictions identified a NES signal exclusively in *Brassica rapa*, *A. thaliana* and *Nicotiana tabacum* eIF3E, though manual alignment analysis revealed putative NES-like sequences across all eIF3E homologs (Fig. 1a).

We next investigated the structural significance of eIF3E domains in plants using Arabidopsis stable lines and *N. tabacum* pollen tube as a model for transient expression. We chose pollen tube as a model system due to its strong reliance on translation and the ability to rapidly manipulate a native single-cell system through gene gun-mediated transient transformation (Kumar and Hafidh 2024). This approach allowed us to study various cellular processes, including morphology, tip growth, protein metabolism, cell wall and plasma membrane organization, and cell-cell communication.

We show that eIF3E is constitutively expressed throughout plant development (Fig. S1b, c). To examine eIF3E subcellular localization, we generated AteIF3E and NteIF3E fusion proteins tagged with YFP fluorescence reporter driven either by native eIF3E promoter or by pollen-specific LAT52 promoter for transient expression in tobacco pollen tubes. These constructs were also introduced into Arabidopsis stable lines. Arabidopsis stable lines expressing native promoter proAteIF3E:AteIF3E::YFP showed AteIF3E::YFP localization predominantly in the cytoplasm from the microspore stage to bicellular pollen (Fig. 1b). At the mature pollen stage, AteIF3E::YFP exhibited distinct localization around the vegetative cell and peri-germ cell membrane, forming a punctate structures surrounding the sperm cells (Fig. 1b). These punctate ribonucleoprotein particles (RNPs) were also present in the vegetative cytoplasm but showed weak or no detectable signal in the nuclei of vegetative or the sperm cells (Fig. 1b). Similarly, LAT52-driven AteIF3E::YFP, proLAT52:NteIF3E::YFP, displayed the same localization pattern as proAteIF3E:AteIF3E::YFP in mature pollen of Arabidopsis stable lines (Fig. 1c). However, in Arabidopsis pollen tubes expressing either proAteIF3E:AteIF3E::YFP, proLAT52:AteIF3E::YFP or proLAT52:NteIF3E::YFP, no longer exhibited the distinct localization at the peri-germ cell membrane or the RNPs punctates (Fig. 1c). Instead, both proteins (AteIF3E::YFP and NteIF3E::YFP) were uniformly distributed in the cytoplasm, indicating a conserved eIF3E subcellular localization from the 2 species (Fig. 1c).

To elucidate the eIF3E localization pattern in tobacco pollen tubes, we transiently expressed proLAT52:NteIF3E::YFP and compared with N- and C-terminal fusion of AteIF3E (proLAT52:AteIF3E::YFP and proLAT52:YFP::AteIF3E, respectively). Our observations revealed that NteIF3E::YFP and both AteIF3E::YFP and YFP::AteIF3E exhibited identical localization pattern in tobacco pollen tubes, with signal detected in the cytoplasm and the nucleus of the vegetative cell (Fig. 1c). To evaluate the functional conservation between AteIF3E and NteIF3E, we complemented the Arabidopsis *eif3e*-1 mutant allele. The *eif3e*-1 allele is embryo-lethal, show reduce pollen germination, poor pollen tube growth and fertilization defects, but it is not required for pollen maturation (Yahalom et al. 2008; Roy et al. 2011; Fig. S1d). Functional complementation assays using proLAT52:AteIF3E::YFP and proLAT52:NteIF3E::YFP constructs effectively rescued the male transmission defect of the *eif3e*-1 allele, with NteIF3E exhibiting slightly higher rescue efficiency compared with AteIF3E (Fig. 1d). Both constructs also complemented the pollen tube growth defect, although AteIF3E performed slightly better than NteIF3E in this assay (Fig. 1e, f). Given the reliability of the transmission assay, which requires minimal manipulation and present clearer genotyping output, the variation in complementation efficiency observed in the *in vitro* pollen tube assay is likely due to technical variability rather than functional divergence between the 2 orthologs. This conclusion is further supported by the high amino acid identity between AteIF3E and NteIF3E (87.6%) as well as their identical subcellular localization in both species.

### The NSF and PCI domains are required for eIF3E subcellular localization and function

To assess the contribution of individual domains on eIF3E protein localization and function, we systematically deleted each conserved domain and generated Arabidopsis stable lines and transiently transformed tobacco pollen tubes expressing domain-deletion variants fused to YFP. Constructs included proUBQ10:AteIF3E^ΔNSF^::YFP, proUBQ10:AteIF3E^ΔNLS^::YFP, and proUBQ10:AteIF3E^ΔPCI^::YFP, alongside their tobacco counterparts proUBQ10:NteIF3E^ΔNSF^::YFP, proUBQ10:NteIF3E^ΔNLS^::YFP, and proUBQ10:NteIF3E^ΔPCI^::YFP.

**Figure 2:**
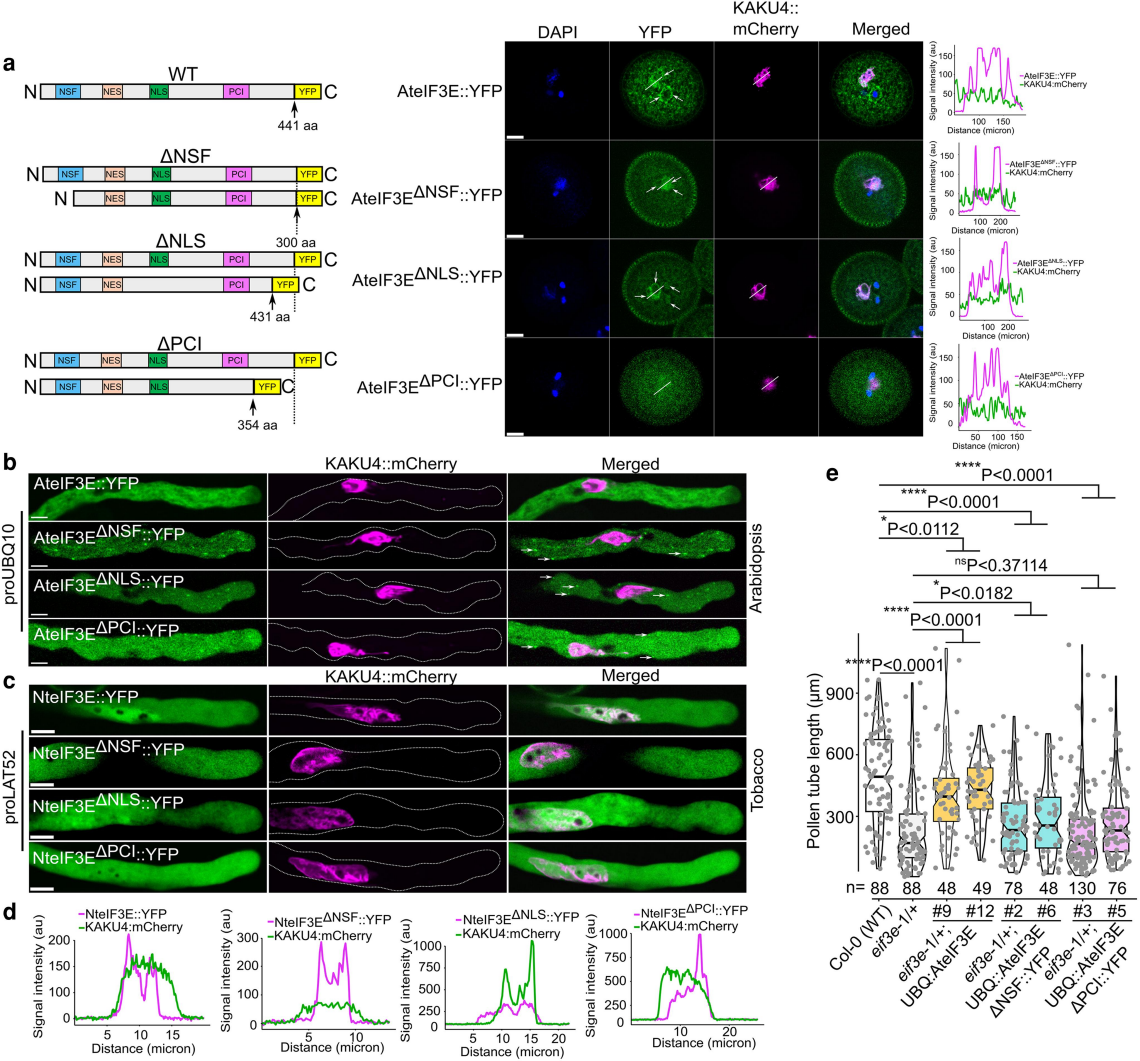
Domains architecture define eIF3E subcellular localization. (a) Subcellular localization of AteIF3E::YFP, AteIF3E^ΔNSF^::YFP, AteIF3E^ΔPCI^::YFP, and AteIF3E^ΔNLS^::YFP in stable Arabidopsis mature pollen. Full-length AteIF3E::YFP localized predominantly in the cytoplasm, with partial accumulation in the vegetative cell nucleus, and showed strong enrichment at the peri-germ membrane of mature pollen, as confirmed by co-localization with the nuclear envelope marker KAKU4. In contrast, all deletion variants lost both peri-germ and vegetative nuclear localization. White arrows indicates the vegetative nuclear or sperm cells localization of all deletion variants whereas full-length AteIF3E localized around the MGU. Plot profile represent the degree of co-localization of AteIF3E and its deletion variants with KAKU4 in the vegetative nuclei as represented by the white line (*n* = 15). Scale bar = 10 *μ*m for all panels (b) In vitro–grown Arabidopsis pollen tubes. While full-length AteIF3E::YFP was uniformly distributed in the cytoplasm but excluded from the clear zone at the tip, all deletion variants formed cytoplasmic protein puncta (arrows) after 4 h of pollen tube germination. White arrows mark formation of cytoplasmic puncta in all the deletion variants, whereas no cytoplasmic puncta were observed in full-length AteIF3E in stable Arabidopsis pollen tube (*n* = 10). Dotted line in the KAKU4::mCherry panel highlights the boundary of the pollen tube. Plot profile indicate that no co-localization of either full-length AteIF3E or its deletion variants with KAKU4, suggesting no vegetative nuclear signal. Scale bar = 10 *μ*m for all the panels. (c) Localization of NteIF3E::YFP and its truncated versions (NteIF3E^ΔNSF^::YFP, NteIF3E^ΔNLS^::YFP, and NteIF3E^ΔPCI^::YFP) in transient tobacco pollen tubes. All deletion variants lacked nuclear signal when co-expressed with KAKU4 as represented by plot profile. Unlike Arabidopsis, these truncations did not frequently generate cytoplasmic puncta in tobacco pollen tubes, however, protein aggregates appeared after 10 h of pollen tube germination (Fig. S2). Scale bar = 10 *μ*m applied to all the panels. (d) Fluorescence intensity profiles across the nucleus showing the degree of co-localization between NteIF3E::YFP or its deletion variants and KAKU4. (e) Complementation analysis of the Arabidopsis *eif3e*-1/+ mutant pollen tube phenotype using AteIF3E deletion variants as represented by Notch-boxplot embedded in violin plot in which center indicates the median and the first and third quartiles indicate 25th and 75th percentiles, and the whiskers extend from minimum to maximum with 1.5 times the interquartile range from the 25th and 75th percentiles, whereas all the data points are shown as gray dots indicates an individual pollen tube length. The ΔNSF construct displayed severely reduced, though not statistically significant, complementation efficiency compared with full-length AteIF3E::YFP, whereas the ΔPCI construct completely failed to rescue the mutant phenotype. Statistical test was performed using the unpaired nonparametric *t*-test using Mann–Whitney *U* test in GraphPad Prism 9.1.1., *n* = range from 48 to 130 for each dataset, # independent lines tested.

In Arabidopsis, deletion of the NSF domain disrupted the typical peri-germ membrane and cytoplasmic RNP puncta pattern of AteIF3E::YFP. Instead, the AteIF3E^ΔNSF^::YFP accumulated diffusely at the peri-germ membrane and aberrantly within the vegetative cell nucleoplasm, overlapping with the nuclear lamina marker KAKU4 (Goto et al. 2020; Fig. 2a). Removal of the NLS led to mislocalization of AteIF3E^ΔNLS^::YFP to both vegetative and sperm cell nuclei, with loss of peri-germ membrane and cytoplasmic RNP puncta (Fig. 2a). While the AteIF3E^ΔPCI^::YFP showed the most severe defect, displaying only diffuse cytoplasmic fluorescence without any RNP puncta formation (Fig. 2a). Consistently, in Arabidopsis pollen, all 3 deletion variants were therefore excluded from nuclei, peri-germ membrane localization and formed cytoplasmic RNP puncta distinct from full-length AteIF3E::YFP (Fig. 2b).

In transient tobacco pollen tubes, wild-type NteIF3E::YFP localized to both nuclei and cytoplasm, whereas all deletion variants exhibited only partial nuclear and diffuse cytoplasmic localization, lacking RNP puncta during early germination (≤6 h; Fig. 2c). By 10 h, however, >50% of NteIF3E^ΔNSF^::YFP–expressing pollen tubes (*n* = 33) formed pronounced cytoplasmic puncta, a phenotype absent in the NLS and PCI deletion variants (Fig. S2a to c). These NteIF3E^ΔNSF^ RNP puncta co-localized with the processing body (PB) marker proDCP5:DCP5::mCherry and the stress granule (SG) marker proRBP47:RBP47::mCherry (Fig. S2d, e). Co-expression with KAKU4::mCherry confirmed nuclear envelope co-localization for wild-type NteIF3E, whereas all deletion variants displayed only partial overlap (Fig. 2c, d).

Overexpression of AteIF3E^ΔNSF^::YFP, AteIF3E^ΔNLS^::YFP, or AteIF3E^ΔPCI^::YFP in stable wild-type Arabidopsis caused dominant-negative effects, including reduced pollen size (16% to 33%, *n* > 500) and shorter pollen tubes after 6 h of pollen germination, phenotypes correlated with YFP-positive pollen grains (Fig. S3a to d). Moreover, the AteIF3E^ΔNSF^::YFP showed reduced complementation of the *eif3e*-1 mutation, whereas AteIF3E^ΔPCI^::YFP failed to complement entirely (Fig. 2e).

Collectively, these results demonstrate that all 3 domains of eIF3E; the NSF, NLS, and the PCI, are essential for proper subcellular localization and function in both Arabidopsis and tobacco pollen tubes. The pronounced phenotypic defects observed in Arabidopsis upon domain deletions underscore an increase sensitivity of Arabidopsis pollen development and pollen tube growth upon eIF3E truncation and loss-of-function.

### AlphaFold3 modeling and *in vivo* co-IP reveal eIF3E association with core octameric subunits in reproductive tissues

To identify proteins interacting with eIF3E in reproductive tissues, we investigated the eIF3E interactome using both computational and experimental approaches. *In silico* structural predictions were performed using AlphaFold3 (Abramson et al. 2024) and ColabFold (Mirdita et al. 2022), which independently modeled interactions between AteIF3E and several eIF3 core subunits. AlphaFold3, an improved protein–protein interaction (PPI) prediction tool based on AlphaFold2, provides more accurate predictions of side-chain and overall protein structures, enhancing PPI forecasts. ColabFold, which incorporates a fast homology search (MMseq2) with AlphaFold2 or RoseTTAFold, offers quicker predictions and visual analysis through UCSF ChimeraX, providing an independent reference for comparison (Zhang and Skolnick 2004; Xu and Zhang 2010; Goddard et al. 2018; Pettersen et al. 2021; Mirdita et al. 2022; Meng et al. 2023; Abramson et al. 2024).

**Figure 3:**
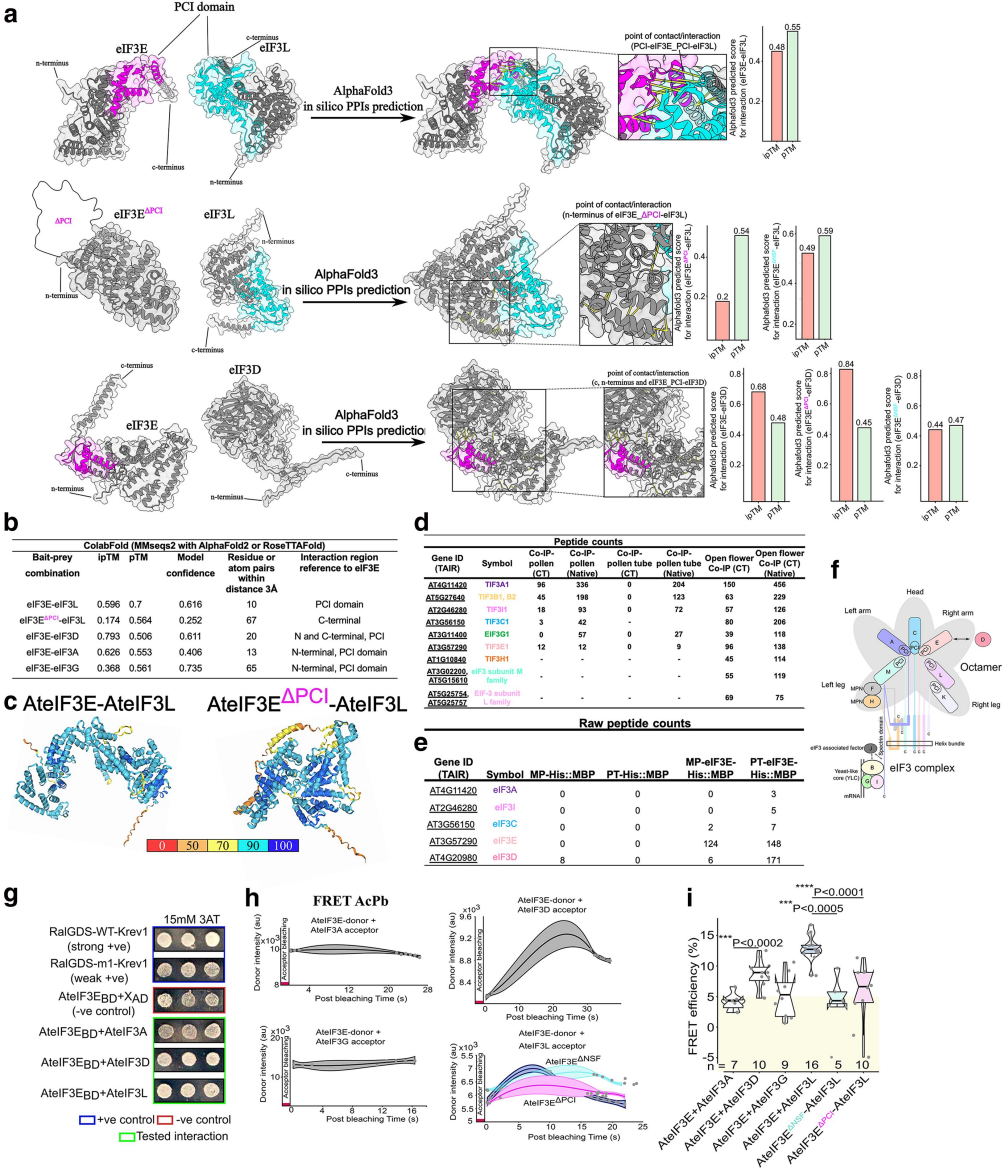
eIF3E interacts with core eIF3 subunits. (a) Predicted 2D structural models of eIF3E interactions with eIF3L and eIF3D were generated using AlphaFold3 and ColabFold (MMseqs2 with AlphaFold2 or RoseTTAFold), and the best models were refined in UCSF ChimeraX. Key contact sites were identified between the PCI domain of eIF3E (pink) and eIF3L (cyan), including a 10-amino acid stretch (yellow). Models of PCI domain–truncated eIF3E with eIF3L are shown; on the right, a zoomed view highlights 67 shifted contact points, indicating that the PCI domain is critical for eIF3E–eIF3L interaction. The lower panels present predicted models of eIF3E with peripheral subunit eIF3D, with highlighted eIF3E–eIF3D contact sites. Bar charts demonstrate the possible interaction between bait (AteIF3E) or prey (AteIF3L and AteIF3D) as represented by ipTM/pTM scores that were calculated by the online AlphaFold server (https://alphafoldserver.com/). Comparative models and prediction scores are provided in Fig. S4. (b) Table summarizing predicted scores generated by ColabFold (MMseqs2 with AlphaFold2 or RoseTTAFold) for ipTM, pTM, and overall model confidence in PPI predictions. (c) Independent ColabFold structural predictions of the eIF3E–eIF3L interaction, validated against a truncated eIF3E PCI domain. Models are color-coded by pLDDT values, reflecting per-residue confidence: dark blue (>90, very high confidence), light blue (70 to 90, confident), yellow (50 to 70, low confidence), and orange (<50, very low confidence) and green represent the low error with high confidence prediction residues as determined by PAE. Contact points and domains were highlighted in UCSF ChimeraX. (d-f) Tables of LC-MS/MS raw peptide counts and schematic summary of the eIF3 octameric complex with individual subunits color-coded, showing interactions detected in AteIF3E co-IP samples from pollen, pollen tubes, flowers, and recombinant NteIF3E in vitro assays. (g) Y2H interaction assays showing interaction between eIF3E with the eIF3D and eIF3L subunits of the octameric complex. Blue square panel represent a strong (RalGDS-WT-Krev1) and weak positive (RalGDS-m1-Krev1) control, whereas red square box highlight negative control AteIF3EBD + XAD (X- empty vector). Green square box shows the interaction of AteIF3E with AteIF3A, AteIF3D and AteIF3L. (h, i) Acceptor photobleach-based FRET analyses confirmed a direct interaction of AteIF3E with AteIF3D and AteIF3L, but not with AteIF3A or AteIF3G, and further demonstrated that the Arabidopsis NSF and PCI domains are essential for the AteIF3E–AteIF3L interaction. Time-course plot of the measured response of donor intensity over time, the median is indicated by the center line; the box spans first and third quartiles (25th to 75th percentile), and whiskers extend to values within 1.5 × the interquartile range. The FRET efficiency for the interaction either with full-length AteIF3E or PCI/NSF domain deletions with selected subunits (AteIF3A, AteIF3D, AteIF3G, and AteIF3L) are represented by Notch-boxplot embedded in violin plot. Statistical differences were calculated in GraphPad Prism 9.1.1 using unpaired nonparametric *t*-test followed by a Mann–Whitney *U* test. *n* = number of samples size (ranges from 7 to 10 cells).

Both algorithms predicted confidence interactions between AteIF3E with AteIF3D and AteIF3L, with consistent interfacial contact sites localized to the C-terminal region of the PCI domain (Figs. 3a to c and S4a to c). AlphaFold3 yielded ipTM/pTM scores of 0.68/0.48 for the eIF3E–eIF3D pair and 0.48/0.55 for eIF3E–eIF3L, while ColabFold reported comparable values of 0.793/0.506 and 0.596/0.7, respectively. In contrast, predicted interactions with the more distant subunits eIF3A and eIF3G had substantially lower confidence scores; 0.17/0.4 (AlphaFold3) and 0.626/0.553 (ColabFold) for eIF3E-eIF3A, and 0.26/0.42 (AlphaFold3) and 0.368/0.561 (ColabFold) for eIF3E-eIF3G (Figs. 3b and S4a). Substitution of the PCI domain in the eIF3E^Δ^PCI–eIF3L model markedly reduced the predicted interaction scores, 0.2/0.54 (AlphaFold3) and 0.174/0.564 (ColabFold) compared with full length AteIF3E-eIF3L interaction (Figs. 3a, b and S4a), supporting the PCI domain as the principal interface mediating contact with eIF3L (Fig. 3a to c).

To experimentally validate these predictions, we performed co-IP using Arabidopsis lines expressing native proAteIF3E:eIF3E::YFP in the *eif3e*-1 background. Proteins were extracted from open flowers, mature pollen grains, and 4 h *in vitro* germinated pollen tubes, and immunoprecipitated using agarose GFP-trap (ChromoTek). Native proAteIF3E::YFP served as a control to filter out nonspecific interactors. We detected bands corresponding to the expected molecular weights of 78.9 kDa (AteIF3E::YFP fusion) and 27 kDa (YFP tag only control) (Fig. S4d, e). Gel-free LC-MS/MS analysis of the eluates revealed co-purification of eIF3E with multiple core and non-core eIF3 subunits in all tissues examined (Fig. S4f). eIF3J, eIF3K, and eIF3L were detected exclusively in open flowers. Several ribosomal proteins, including RPS5A, RPS13A, RPL8, RPL12B/C, and RPL7D, were also enriched in the eIF3E::YFP complex (Table S1), suggesting a close association between eIF3E and the translational machinery.

To assess conservation of the eIF3E interactome, we reconstituted the complex in *N. tabacum* pollen tubes using recombinant His–MBP::NteIF3E purified by immobilized Ni^2+^-charged metal affinity chromatography (IMAC). Following incubation with protein extracts from mature pollen and 4 h pollen tubes, co-purified proteins were analyzed by LC-MS/MS. NteIF3E retained a distinct set of proteins relative to His–MBP controls, including eIF3A, eIF3C, eIF3D, and eIF3I (Figs. 3d and S4g, h), consistent with the Arabidopsis eIF3E interactome. Ribosomal proteins (RPS6, RPS14, RPS16) and mitochondrial homeostasis enzymes including ATP synthase, succinate dehydrogenase, NADH dehydrogenase, and glutamate dehydrogenase, as well as heat-shock response related proteins (HSP70 and BIP1) were also enriched (Table S1), implicating eIF3E in the coordination of translation and energy metabolism during pollen germination and tube growth.

In total, the comprehensive proteomic profiling of Arabidopsis eIF3E::YFP co-immunoprecipitates identified 1,342 proteins in inflorescences, 1,478 in mature pollen, and 239 in pollen tubes (Fig. S5a). These interactors were enriched for ribosomal, cytoplasmic, and membrane-associated proteins, as well as RNA-binding proteins (Fig. S5b). Functional enrichment analysis revealed strong associations with carbon metabolism, nucleotide biosynthesis, and secondary metabolite pathways essential for pollen development and tube elongation (Fig. S5c, d). Cluster and heat map analyses grouped interactors into 5 major classes: eIF3 complex subunits, ribosomal proteins, mitochondrial proteins, stress-related proteins, and plasma membrane associated proteins (Fig. S5e). A gene-by-gene survey indicated that the AteIF3E::YFP interactome was enriched in membrane-associated proteins highly expressed in pollen and pollen tubes, including fructose-bisphosphate aldolase 4, cell wall invertase 2, plant LIM-domain protein 2A, germin-like protein 8, glutathione transferase, mitogen-activated protein kinase 8, Mucin-like protein, and pollen-specific LIM protein 2C (Fig. S5f).

Direct physical interactions between AteIF3E and specific eIF3 subunits were further verified using yeast 2-hybrid (Y2H) and Acceptor photobleach Förster resonance energy transfer (FRET) assays. In the Y2H assay, positive interactions were detected between AteIF3E-AteIF3D and AteIF3E-AteIF3L pairs on plates containing 15 mM-3AT (Fig. 3e). No interaction was observed between AteIF3E-AteIF3A pairs (Fig. 3e). In the FRET analysis, AteIF3E directly interacted with AteIF3D and AteIF3L (Fig. 3f, g), while no interaction was observed with AteIF3A or AteIF3G, as indicated by the low FRET efficiency (Fig. 3f, g). Deletion of either the NSF or PCI domain abolished the FRET signal with AteIF3L, indicating that both domains are required for this interaction (Fig. 3f, g).

Together, these results demonstrate that eIF3E directly associates with core octameric subunits eIF3D and eIF3L via its PCI and NSF domains, and that the eIF3E interaction network is conserved between Arabidopsis and *N. tabacum*. Moreover, the eIF3E interactome in reproductive tissues is enriched in ribosomal and energy metabolism components, emphasizing eIF3E central role in translational regulation during pollen germination and pollen tube growth.

### The C-terminal PCI domain is required for efficient eIF3E dissociation from translating ribosomes

**Figure 4:**
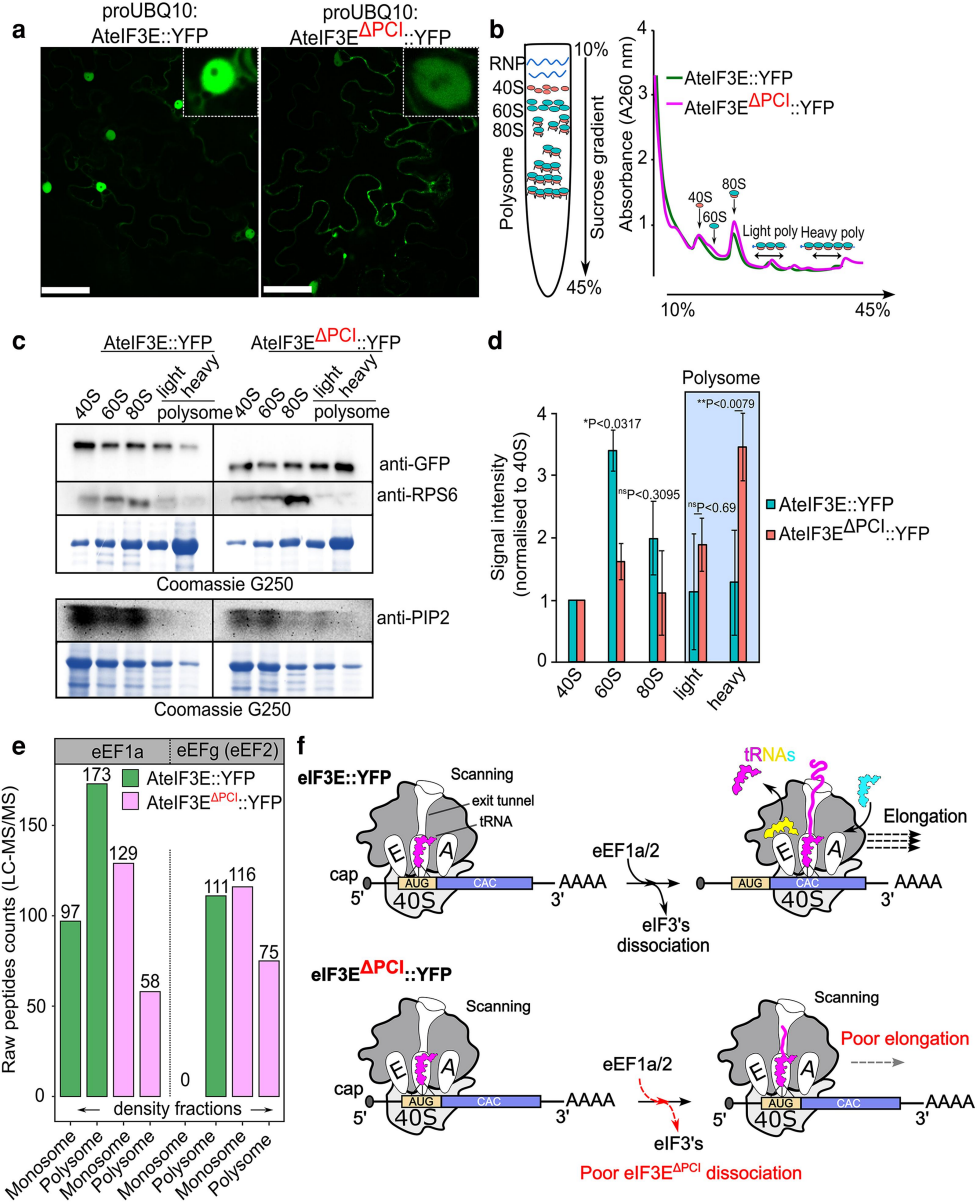
PCI domain is required for eIF3E efficient dissociation post translation initiation. (a) Confocal images depicting expression of full length AteIF3E::YFP and AteIF3E^ΔPCI^::YFP in *N. benthamiana* pavement cells used for polysome fractionation. Inset shows a nuclear-cytoplasmic localization of AteIF3E::YFP and a more cytoplasmic AteIF3E^ΔPCI^::YFP localization. Scale bar = 50 *μ*m applied for each presented images (b) Polysome fractionation from *N. benthamiana* leaf tissues expressing AteIF3E:YFP and AteIF3E^ΔPCI^::YFP under the UBQ10 promoter. A sucrose density gradient of 10% to 45% was used to separate free RNPs, 40S, 60S, 80S ribosomal subunits, as well as light (light poly) and heavy (heavy poly) translating polysomes fractions with uninterrupted scanning at 260 nm. *n* = 6 replicates. (c, d) Immunoblot analysis of full-length AteIF3E::YFP and AteIF3E^ΔPCI^::YFP across gradient fractions. Signal intensities were normalized to the 40S subunit. Compared with full-length AteIF3E::YFP, AteIF3E^ΔPCI^::YFP displayed prolonged association with both light and heavy polysomes. Antibody against ribosomal protein S6 (eS6) served as a positive light-heavy polysome marker, while plasma membrane protein PIP2 served as a negative, non-polysomal control. Data represent 6 independent experiments, each normalized to the corresponding 40S fraction. Error bars indicate ± Sd (*n* = 6). Statistical significance was determined in GraphPad Prism using unpaired nonparametric *t*-test followed by Mann–Whitney *U* test. (e) LC–MS/MS analysis were performed with at least 3 replicates from 80S monosome and polysome fractions. Expression of AteIF3E^ΔPCI^::YFP resulted in reduced abundance of elongation factors eEF1a and eEFg in 80S and polysomes fractions relative to full-length AteIF3E::YFP. (f) Schematic model illustrating the proposed effect of prolonged association of eIF3E^ΔPCI^ with 80S/polysome fractions. Impaired dissociation of eIF3E^ΔPCI^ may hinder efficient recruitment of elongation factors, thereby reducing ribosome progression and overall protein synthesis.

In eukaryotic translation, the PCI domain of eIF3E mediates interactions with both core and non-core eIF3 subunits, the eIF4F cap-binding complex, and the methionyl-tRNA carrier eIF2α-GTP ternary complex to assemble the 43S preinitiation complex (Valášek et al. 2017). Following translation initiation, helical extensions of the PCI domain facilitate eIF3 complex disassembly and ribosomal recycling through GTP-GDP hydrolysis by eIF2 allowing entry of elongation factors eEF1a and eEF2 and initiate peptide elongation for protein synthesis (Masutani et al. 2007; Hinnebusch and Lorsch 2012; Querol-Audi et al. 2013; Simonetti et al. 2016; Smith et al. 2016; Castellano and Merchante 2021; Brito Querido et al. 2024). To assess the role of the PCI domain in eIF3E recycling, we expressed proUBQ10:AteIF3E::YFP and proUBQ10:AteIF3E^ΔPCI^::YFP in *Nicotiana benthamiana* leaves and analyzed their association with ribosomal fractions by sucrose density gradient centrifugation (Mustroph et al. 2009a, 2009b; Hafidh et al. 2018). Ribosome profiling resolved distinct subfractions corresponding to free 40S and 60S subunits, monosomes (80S), and light and heavy polysomes (Fig. 4a, b).

Immunoblot analysis detected both wild-type and PCI-deleted eIF3E variants across 40S to 80S fractions and translating light-heavy polysomes (Fig. 4c). Normalization of eIF3E levels relative to the 40S subunit revealed a characteristic decline of wild-type AteIF3E::YFP from monosomes to light polysomes, with minimal signal in heavy polysomes, indicating efficient dissociation after translation initiation (Fig. 4c, d). In contrast, AteIF3E^ΔPCI^::YFP exhibited a nearly 3-fold higher retention in 80S, light, and heavy polysomal fractions (Fig. 4c, d), suggesting that the PCI domain is required for eIF3E release from active translation complexes.

Because translation elongation requires the dissociation of eIF3 and eIF2 complexes to allow recruitment of elongation factors (Hinnebusch and Lorsch 2012), we next analyzed whether PCI domain deletion impairs this transition. LC-MS/MS analysis of proteins from 80S and polysomal fractions revealed that elongation factors eEF1α and eEF2 were significantly enriched in polysomes from wild-type AteIF3E::YFP–expressing samples but markedly reduced in those expressing AteIF3E^ΔPCI^::YFP (Fig. 4e). These results indicate that persistent association of AteIF3E^ΔPCI^ with translating ribosomes hinders elongation factor recruitment and likely impedes translation elongation (Fig. 4f).

Together, these findings demonstrate that the PCI domain is not only required for incorporation of eIF3E into the eIF3 translation initiation complex but is also essential for eIF3E efficient dissociation after translation initiation, thereby ensuring proper transition to polypeptide elongation and sustained protein synthesis.

### RIP-seq co-IP identifies eIF3E mRNA targets in pollen tubes

In yeast and Drosophila, eIF3E co-immunoprecipitates with a limited set of mRNAs involved in RNA metabolism, mitochondrial homeostasis, tissue differentiation, and cell growth (Sha et al. 2009; Fujii et al. 2021; Lin et al. 2020). Notably, eIF3E lacks a canonical RRM for direct RNA binding (Fig. 1). Instead, eIF3E likely associates with target mRNAs through other subunits of the octameric eIF3 complex, such as eIF3B and eIF3H, as well as via direct interaction (alongside eIF3C and eIF3D) with eIF4G/E of the eIF4F complex, which bridges the cap-binding protein eIF4E and PABP (Lefebvre et al. 2006; Villa et al. 2013; Fig. S8). To identify eIF3E-bound mRNAs in tobacco pollen tubes, we performed RIP-seq following formaldehyde crosslinking. Pollen tubes expressing proLAT52:AteIF3E::YFP were analyzed 6 h post-transformation and compared with wild type pollen tubes control. Immunoprecipitation was conducted using anti-GFP magnetic beads (ChromoTek) across 3 biological replicates, followed by Illumina RNA sequencing. The resulting datasets revealed highly reproducible differences between AteIF3E::YFP and wild type pollen tubes in both input and the eluate fractions (Fig. S6a).

Reads were mapped to the tobacco reference genome (https://solgenomics.net/organism/Nicotiana_tabacum/genome). After filtering low-quality reads, we identified 10,062 expressed transcripts. To improve specificity, we retained transcripts enriched by at least 2-fold in the AteIF3E::YFP eluate compared with input (FC_eIF3E/FC_WT, >2), yielding 453 enriched transcripts. We applied further stringency filtering selecting only transcripts with minimal input variation between AteIF3E::YFP and WT (log_2_ FC_I_YFP-eIF3E vs WT [−1;1]), resulted in a final set of 191 high-confidence eIF3E-associated mRNA targets (Fig. S6b; Table S2). Gene ontology (GO) analysis revealed enrichment in 3 primary biological processes; RNA processing (*P* < 0.002, 6 genes), RNA modification (*P* < 0.005, 3 genes), and ncRNA metabolic processing (*P* < 0.001, 4 genes). These targets include Class I and II aminotransferases, Actin-binding FH2 formins, nucleolar protein 6, nuclear RNA-binding proteins, and hyaluronan mRNA-binding protein (Habp4), the latter involved in ribosome stabilization. Additional identified factors include ribonuclease 3-like proteins, DNA-directed RNA polymerase III (involved in 5S rRNA, tRNA, and snRNA synthesis), SUMO ligase (for SUMOylation-driven regulation), tRNA-splicing endonuclease and U3 small nucleolar RNA-associated proteins.

To validate the *in vivo* association of AteIF3E with its target mRNAs, we selected 4 candidates from the AteIF3E-YFP RIP-seq dataset, representing a range of enrichment levels; U3 small nucleolar RNA-associated protein 15 homolog (*U3 snRNAa15*, FC = 13.3, *P* < 0.25, *Nitab4.5_0001651g0120*), Ribosomal L9-like protein (*rPL6-SH3*, FC = 5.3, *P* < 0.13, *Nitab4.5_0002123g0080*), aminocyclopropane-1-carboxylate synthase (*Prop1AT*, FC = 179, *P* < 0.23, *Nitab4.5_0000439g0010*), and GPI ethanolamine phosphate transferase (*EthAP*, FC = 11.7, *P* < 0.2, *Nitab4.5_0001468g0100*). To track these mRNAs, we tagged their 3′-ends with the PP7 RNA aptamer (AUAUGG) from *Pseudomonas* RNA phage and co-expressed them with reporters proUBQ10:AteIF3E::YFP and proUBQ10:PP7::mCherry, a coat protein derived from bacteriophage that specifically binds to the PP7-tagged mRNA (Fig. S6d; Lim and Peabody 2002; Kumar and Hafidh 2024).

Live-cell imaging was conducted on *N. benthamiana* leaf pavement cells expressing the mRNA targets (U3snRNA15-PP7, rPL6-SH3-PP7, Prop1AT-PP7 or EthAP-PP7) at a ratio of 3:1 target mRNA to reporter. Live cell imaging revealed a clear distinct association of AteIF3E::YFP with its respective RNA targets, as evidenced by the formation of numerous RNP puncta bound to AteIF3E::YFP (Fig. S6d). Some mRNA targets (AteIF3E::YFP-U3snRNA15 and AteIF3E::YFP-rPL6-SH3) generated RNP puncta in both the nucleus and cytoplasm, while others, AteIF3E::YFP-Prop1AT and AteIF3E::YFP-EthAP formed puncta solely in the cytoplasm (Fig. S6d). Notably, these RNP puncta only appeared upon co-expression of the target mRNA with the reporter, but not when AteIF3E::YFP was expressed alone in the absence of the target RNA (Fig. S6d).

To verify the direct association of the AteIF3E derived puncta with target mRNAs, we performed affinity purification of AteIF3E::YFP after co-expressing each target mRNA in pavement cells. The results of RNA precipitation following YFP co-IP confirmed, via semi-RT-qPCR, that the selected target mRNAs specifically co-immunoprecipitated with the AteIF3E::YFP but not with the free YFP control (Fig. S6e).

### eIF3E regulates mRNA translatability through MC1 to MC3 translational repressor-enhancer *cis*-elements

**Figure 5:**
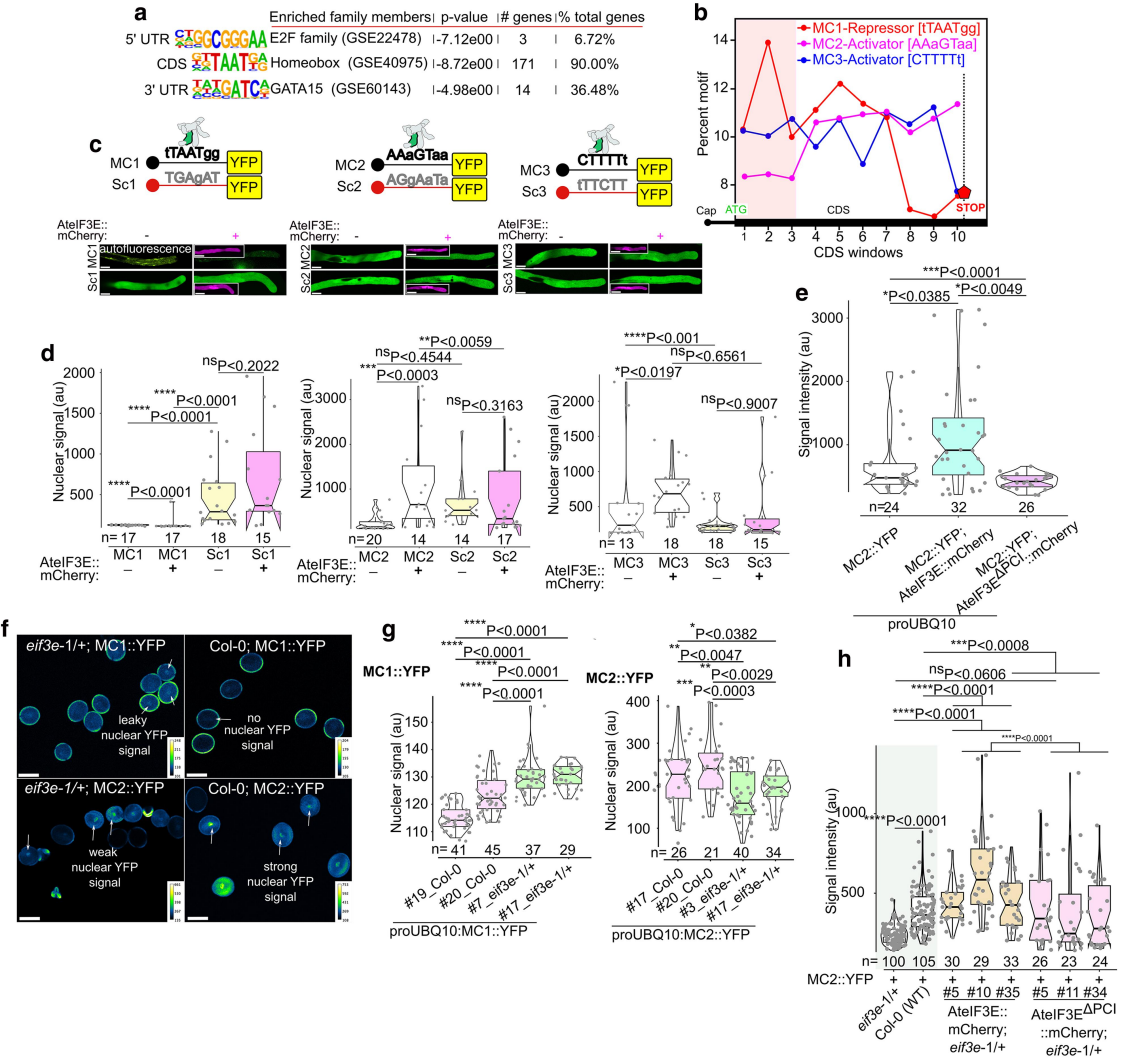
RIP-seq eIF3E-associated transcripts comprise of MC1-MC3 *cis*-elements with translation repression and activation capabilities. (a) Motif analysis using HOMER (R function *findMotifs*) on 191 enriched AteIF3E co-immunoprecipitated transcripts revealed overrepresented motifs within the 5′UTR, CDS, and 3′UTR of eIF3E target mRNAs (b) Positional distribution of the top 3 CDS *cis*-elements MC1 (tTAATgg), MC2 (AAaGTaa), and MC3 (CTTTTt) across the coding regions of 191 enriched target transcripts. MC1, a translational repressor, is strongly enriched near the CDS start but depleted toward the CDS end, whereas MC2 and MC3, both translational activators, show a more uniform distribution that increases following the MC1 peak relative to the ATG start codon. The pink shading highlights CDS regions where MC1 repressor activity is highest. *N* = 191 (c, d) A YFP mRNA reporter fused to 1 of the 3 top-selected CDS-associated motifs was expressed in transiently transformed tobacco pollen tubes. Each YFP construct contained 4 tandem repeats of the core motif sequence, fused in-frame with YFP under the UBQ10 promoter. Reporter constructs were transiently expressed in wild-type tobacco pollen tubes, either alone or co-expressed with the AteIF3E::mCherry effector. YFP intensity was quantified within a fixed ROI at the pollen tube tip 6 h post-transformation. Scrambled tandem repeat sequences were generated in silico as negative controls. Scale bar = 10 *μ*m for the GFP channel and 15 *μ*m for the mCherry channel was set for all the presented images in each panel. (e) A similar co-expression of the MC2::YFP reporter with a non-functional AteIF3E^ΔPCI^::mCherry under the UBQ10 promoter strongly inhibited MC2::YFP translation, confirming its eIF3E dependence. (f to h) The translational repression by MC1 and the eIF3E-dependent activation by MC2 were further validated in *eif3e*-1/+ Arabidopsis knockdown lines and in transient *N. benthamiana* leaves (Fig. S7). In Arabidopsis stable lines, YFP intensity was quantified in mature pollen in Col-0 or in the *eif3e*-1/+ mutant background using fixed ROI around the vegetative cell nuclei. (f, g) *eif3e*-1 mutant pollen grains showed reactivation of MC1::YFP and reduction in MC2::YFP translational activation. The images displayed using a color intensity code with the pixel range set for all images (reflecting differences in protein expression levels between mutant *eif3e*-/+ and Col-0 expressing MC1 and MC2 motifs). White arrows indicate the variation in protein levels of YFP reporter in mutant *eif3e*-/+ and Col-0 expressing MC1 and MC2 motifs. (h) Quantitative analyses in independent complemented *eif3e*-1/+ Arabidopsis lines demonstrated that MC2-dependent translation activation strictly requires functional AteIF3E. YFP intensity quantification was performed similarly as in (f). Scale bar = 10 *μ*m applied to all images of figure f panel. Center line in boxplot of above presented Fig. 5 panels (d, e, g, h) represent the median and the first and third quartiles indicate 25th and 75th percentiles, and the whiskers extend from minimum to maximum with 1.5 times the interquartile range from the 25th and 75th percentiles. Statistical differences were evaluated in GraphPad Prism 9.1.1 using unpaired nonparametric *t*-test employed by a Mann–Whitney *U* test. *n*, population size. *n* = number of samples size and # independent lines tested.

Next, we conducted a computational search for repetitive motifs across the entire gene structure of eIF3E co-immunoprecipitated high confidence mRNA targets using the HOMER R studio findMotifs script (http://homer.ucsd.edu/homer/motif/). This analysis revealed 3 enriched motifs associated with E2F-family genes in the 5′UTR of eIF3E target mRNAs, which accounted for 6.67% of the total target transcripts (3 out of 140 mapped genes, *P* < 0.0001). In the coding region (CDS), 14 motifs linked to Nkx6.1 Hox family, C3H, and C2C2 gata-dof families were identified, representing more than 90% of the total eIF3E target transcripts (182 out of 191 genes, *P* < 0.0001). Additionally, 9 motifs in the 3′UTR, with the strongest motifs associated with GATA15, WRKY55, and SPDEF gene families, were present in 5% to 36% of the total target transcripts (14 out of 140 genes, *P* < 0.002) (Fig. 5a). The 3 enriched motif families in the CDS region; E2F, Nkx6.1 Hox, and C3H/C2C2 gata-dof, are linked to cell fate specification and patterning, membrane morphology, cell cycle regulation, development, and stress resistance.

Since these CDS region motifs were over-represented (90% of the eIF3E RIP-seq target mRNAs), we focused on characterizing the CDS motifs with a dominant pattern around the core sequence compared with other flanking di- or tri-nucleotides (Fig. S6c). We selected the 3 most overrepresented CDS motif profiles, with consensus sequences TAATga/g, AAgGTaa, and CTTTTt, and analyzed their distribution among the 182 enriched eIF3E mRNA targets. We termed these motifs, Motif CDS 1 (MC1), MC2, and MC3, respectively (Fig. 5a, b). Notably, all 3 motifs, MC1 (TAATga/g), MC2 (AAgGTaa), and MC3 (CTTTTt), appeared together in tandem on the same target transcripts. Further analysis of their distribution across the 182 target transcripts revealed that MC1 was predominantly located at the start of most CDS regions, while MC2 and MC3 were frequently found downstream of MC1 (Fig. 5b).

To assess the influence of each motif on translation, we generated reporter constructs containing 3 tandem repeats of each motif type, determined by their average frequency occurrence per target mRNA. These constructs were then fused to the 5′-end of a YFP reporter RNA under the control of UBQ10 promoter. We assessed the translatability of YFP in transiently transformed tobacco pollen tubes as a native tissue, as well as in *N. benthamiana* leaf pavement cells, with both system expressing the native NteIF3E. Strikingly, the MC1::YFP *cis*-element completely suppressed translation of YFP reporter mRNA (Fig. 5c). The MC1-mediated translation repression was further enhanced when co-expressed with AteIF3E::mCherry in both tissue types (Figs. 5c, d and S7a to d). However, when the MC1 consensus sequence (MC1-TAATGa/g) was scrambled (Sc1-TGAgAT), the repression capability was abolished, particularly in the absence of excess AteIF3E::mCherry (Figs. 5c, d and S7a to d). Instead, Sc1 exhibited partial activation of the YFP mRNA translation (Figs. 5c, d and S7a to d). These results indicate that AteIF3E mediating translational repression via MC1 *cis*-element. Conversely, the MC2 and MC3 motifs significantly boosted YFP mRNA translation, with expression level rising over 500-fold compared with MC1::YFP (Figs. 5c, d and S7a to d). The addition of excess AteIF3E::mCherry further amplified this effect, enhancing translation by 1.5- to 6-fold for MC2 and MC3, respectively. When the MC2 and MC3 sequences were scrambled (MC2-AAgGTaa to Sc2-AggAaTa and MC3-CTTTTt to Sc3-tTTCTT), there was marked reduction in YFP translation even in the presence of excess AteIF3E::mCherry (Figs. 5c, d and S7a to d). To further support that MC2 to MC3 motifs activation is eIF3E-dependent, we transiently co-expressed MC2::YFP with AteIF3E^ΔPCI^::mCherry in tobacco pollen tubes and found that PCI domain deletion significantly represses MC2::YFP translatability compared with WT AteIF3E::mCherry co-expression (Fig. 5e). The repressive activity of the AteIF3E^ΔPCI^ was not as effective due to the expression of endogenous NteIF3E wild type copy in tobacco pollen tubes. To exclude any transcriptional effects of MC1 to MC3 on YFP RNA levels, we measured total expression of YFP RNA levels in the leaf pavement cells of *N. benthamiana*. Our results demonstrate that YFP RNA levels were uniform in all constructs, regardless of the motif type or the presence of excess eIF3E (Fig. S7c). Western blot analysis further confirmed that MC1 functions as a translational repressor, while MC2 and MC3 act as translational enhancers of the YFP-reporter mRNA (Fig. S7c).

To further confirm the mechanism by which the MC motifs operates in stable genetic context and determine whether their activities depend on AteIF3E, we created stable Arabidopsis transgenic lines that express MC1::YFP and MC2::YFP in both the wild-type Col-0 and *eif3e-*1/+ mutant backgrounds. Quantification of YFP intensity in mature pollen grains from both Col-0 and *eif3e-*1/+ primary transformants demonstrated that MC1 and MC2 motifs activities solely dependent on eIF3E in Arabidopsis (Fig. 5f, g). We observed that the expression of MC1::YFP was clearly suppressed in Col-0 pollen expressing native AteIF3E, nevertheless, this suppression was alleviated in the *eif3e*-1/+ heterozygous mutant pollen population (Fig. 5f, g). In contrast, the MC2::YFP reporter exhibited high expression levels in the Col-0 background, while its activity was reduced in *eif3e*-1/+ heterozygous pollen (Fig. 5f, g). Moreover, we also generated stable Arabidopsis lines co-expressing AteIF3E::mCherry, AteIF3E^ΔPCI^::mCherry and MC2::YFP motif in the *eif3e*-1/+ mutant background to further confirm the dependency of MC2-containing YFP reporter translatability with AteIF3E (Fig. 5h). Signal intensity quantification of MC2::YFP mRNA reporter from pollen revealed that the AteIF3E::mCherry fully restored the MC2 translation activation of the YFP reporter in *eif3e*-1/+ heterozygous pollen, whereas AteIF3E^ΔPCI^::mCherry could not, indicating that the PCI domain may be important for interaction of the eIF3E with the MC2 containing mRNA targets and for full eIF3E functionality (Fig. 5h). Collectively, these findings provide compelling evidence that MC2 and MC3 function as eIF3E mediated translational enhancer elements within the coding sequence (CDS) of target mRNAs. We propose that eIF3E regulates mRNA translation efficiency and protein homeostasis by co-ordinating the repressive effects of predominantly upstream MC1 *cis*-motifs with the translational activation of downstream motifs (MC2/MC3) in a sequential manner, thus ensuring a balanced translational output for the target mRNA (Fig. S7d).

### eIF3E-mediated translational activation of MC2 to MC3 enhancer *cis*-elements requires a functional PCI domain and PCI-phosphosites

Here we shown that deletion of the PCI domain significantly disrupts eIF3E nuclear localization, impairs its interaction with eIF3L, and prevent its efficient dissociation from the translation initiation complex (Figs. 2 to 4). Moreover, post-translational phosphorylation can serve as a rapid transient activation signal or an inhibitory modification that regulates protein function. In eukaryotes, approximately 86% of protein phosphorylation occurs on serine residues, 12% on threonine, and 2% on tyrosine (Schwartz and Murray 2011; Nishi et al. 2014).

**Figure 6:**
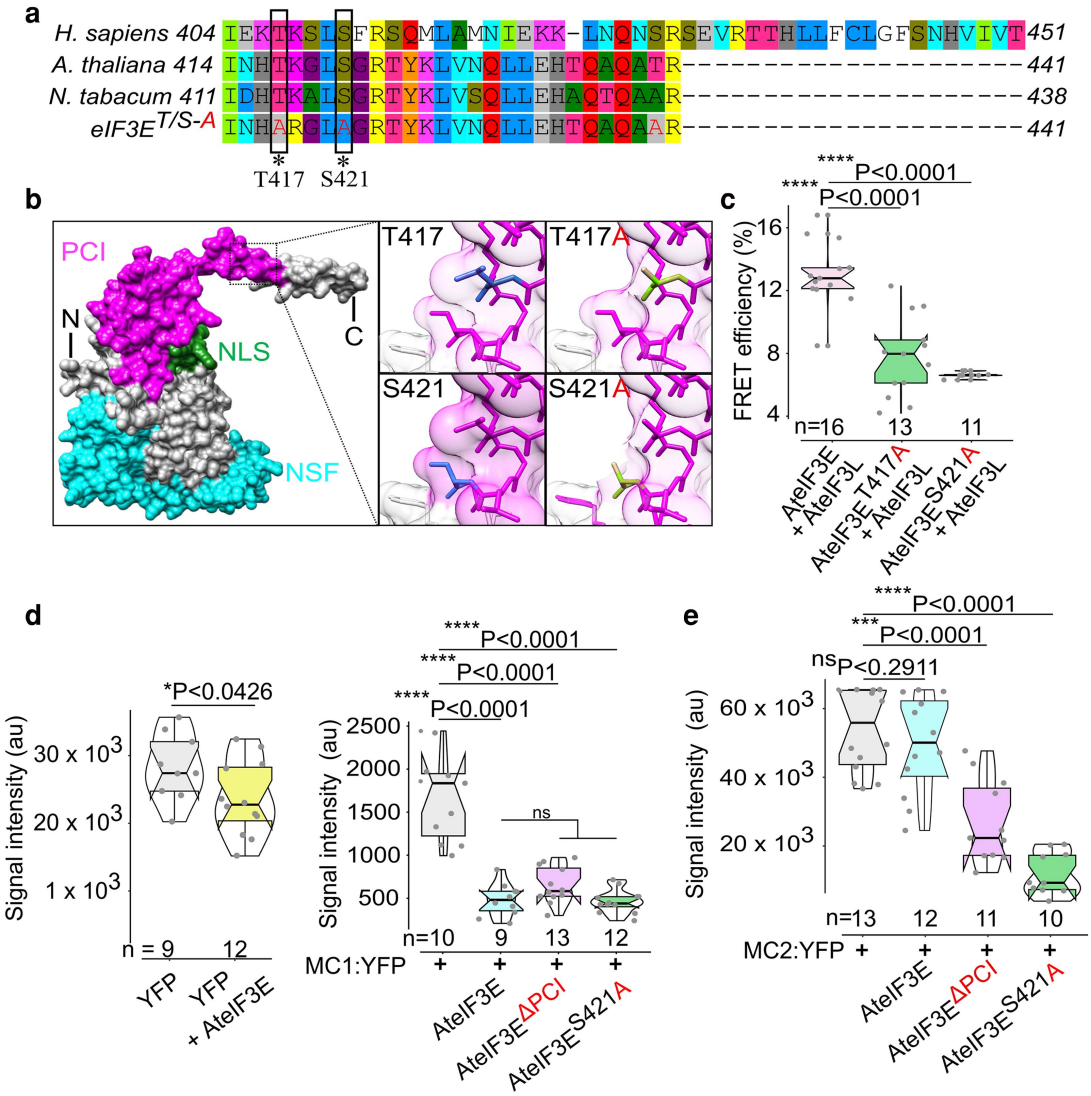
AteIF3E^ΔPCI^ deletion and AteIF3E^T421A^ phosphodead inhibit activation of MC2 translation enhancer element in vivo. (a) CLUSTALW multiple sequence alignment showing eIF3E mutated conserved Threonine (eIF3E^T417A^) and Serine (eIF3E^S421A^). (b) Schematic 2D model of eIF3E constructed using UCSF ChimeraX revealed that the phosphosites are located at the PCI domain and a rotamer module highlighting mutagenized residues. Model was adapted from the SWISS model homology search method. Inset, highlight a 2D orientation of the mutagenized residues from polar uncharged Ser/Thr (S/T) to a nonpolar hydrophobic Ala (a) to create phosphodead eIF3E. (c) Both single mutant phosphodead AteIF3E^T417A^ and AteIF3E^S421A^, lost interaction with AteIF3L subunit of the octameric complex. (d) Translational assay with free YFP control, repressor MC1::YFP alone (in WT), or co-expressed with full length AteIF3E, PCI deletion variant AteIF3E^ΔPCI^ or with a phosphodead AteIF3E^T421A^ mutant variant in *N. benthamiana* leaf tissues. Full length AteIF3E::mCherry strongly suppressed MC1::YFP translation. Comparable suppression of MC1::YFP was also observed following AteIF3E^ΔPCI^ and AteIF3E^T421A^ phosphodead mutant variants albeit by the background native wild type NteIF3E expression. (e) In parallel assays, MC2::YFP was activated by AteIF3E::mCherry, but not beyond the background activation observed with WT NteIF3E. In contrast, both AteIF3E^ΔPCI^ and the phospho-dead variant AteIF3E^T421A^ strongly inhibited MC2::YFP translation, indicating that these mutant forms interfere with MC *cis*-element–mediated activation of the MC2::YFP RNA reporter. The embedded notch-plot in violin plot, center line represent the median and the first and third quartiles indicate 25th and 75th percentiles, and the whiskers extend from minimum to maximum with 1.5 times the interquartile range from the 25th and 75th percentiles. Statistical differences were calculated in GraphPad Prism 9.1.1 using unpaired nonparametric *t*-test followed by a Mann–Whitney *U* test. *n*, population size. Statistical differences of panels (c, d, e) were calculated in GraphPad Prism 9.1.1 using unpaired nonparametric *t*-test employed by a Mann–Whitney *U* test.

To identify potential phosphorylation sites in AteIF3E, we utilized ProteomicsDB (https://www.proteomicsdb.org, Mergner et al. 2020) and MusiteDeep (http://www.musite.net), revealing 2 conserved phosphosites within the PCI domain; Threonine 417 (Thr417) and Serine 421 (Ser421), which are conserved in both mammals and plants (Fig. 6a, b). To investigate their functional role, we generated phosphodead variants by substituting the polar, uncharged residues Thr417 and Ser421 with nonpolar hydrophobic alanine (Ala/A), and created AteIF3E^T417A^ and AteIF3E^S421A^ phosphodeads, respectively (Fig. 6a, b).

To assess the molecular effects of eIF3E dephosphorylation within the PCI domain, we examined the interaction between the phosphodead variants and eIF3L using the FRET acceptor photobleaching method. The control interaction of wild type full length AteIF3E::mTurquoise with AteIF3L::mVenus showed an *in vivo* FRET efficiency of 13.5% ± 2.08, confirming their interaction (Fig. 6c). However, similar to the effects observed with PCI domain deletion, both AteIF3E phosphodead variants displayed significantly reduced interaction with AteIF3L, with FRET efficiencies of <7.7% ± 0.12 for AteIF3E^T417A^-AteIF3L pair and <6.5% ± 0.19 for AteIF3E^S421A^-AteIF3L pair (Fig. 6c). This suggests that phosphorylation at T417 and S421 residues is critical for maintaining the eIF3E interaction with eIF3L. It is yet to be investigated, whether the phosphodead eIF3E variants retain interactions with other subunits of the eIF3 complex. To investigate the functional effects of eIF3E dephosphorylation and PCI domain deletion, we compared the activities of AteIF3E^ΔPCI^::FLAG and AteIF3E^S421A^::FLAG with wild type full length AteIF3E::mCherry under the UBQ10 promoter. We assessed their impact on the translational repressor MC1-YFP and the translational enhancer MC2-YFP reporters by quantifying YFP fluorescence intensity following their co-expression. As expected, AteIF3E::mCherry effectively suppressed MC1::YFP translation (Fig. 6d). Interestingly, both AteIF3E^ΔPCI^::FLAG and AteIF3E^S421A^::FLAG also repressed MC1::YFP translation, albeit due to the expression of endogenous wild type eIF3E in *N. benthamiana* leaves (Fig. 6d). In contrast, the presence of excess AteIF3E::mCherry significantly enhanced MC2::YFP translation when compared with YFP translation without the MC2 *cis*-element (Fig. 6e). However, co-expression of MC2::YFP with either AteIF3E^ΔPCI^::FLAG or AteIF3E^S421A^::FLAG led to a 2.5- to 4.5-fold reduction in MC2::YFP translation, respectively (Fig. 6e). These findings suggest that eIF3E^ΔPCI^ and eIF3E^S421A^ act as translational inhibitors, potentially functioning as dominant-negative interfering with the activity of the wild type eIF3E and inhibit translation.

### Depletion of the eIF3E PCI domain or PCI-phosphosites mutagenesis impair pollen tube growth

To assess the phenotypic impact of AteIF3E^ΔPCI^ mislocalization, its inefficient dissociation post-translation initiation, and the inability of the phosphomutated forms AteIF3E^T417A^ and AteIF3E^S421A^ to interact with AteIF3L or to activate translation of MC2::YFP mRNA reporter (Fig. 6), we compared the growth rates of tobacco pollen tubes transiently expressing proUBQ10:AteIF3E::YFP to pollen tubes expressing proUBQ10:AteIF3E^ΔNSF^::YFP, proUBQ10:AteIF3E^ΔNLS^::YFP, proUBQ10:AteIF3E^ΔPCI^::YFP, proUBQ10:AteIF3E^T417A^::YFP, and proUBQ10:AteIF3E^S421A^::YFP, respectively.

**Figure 7:**
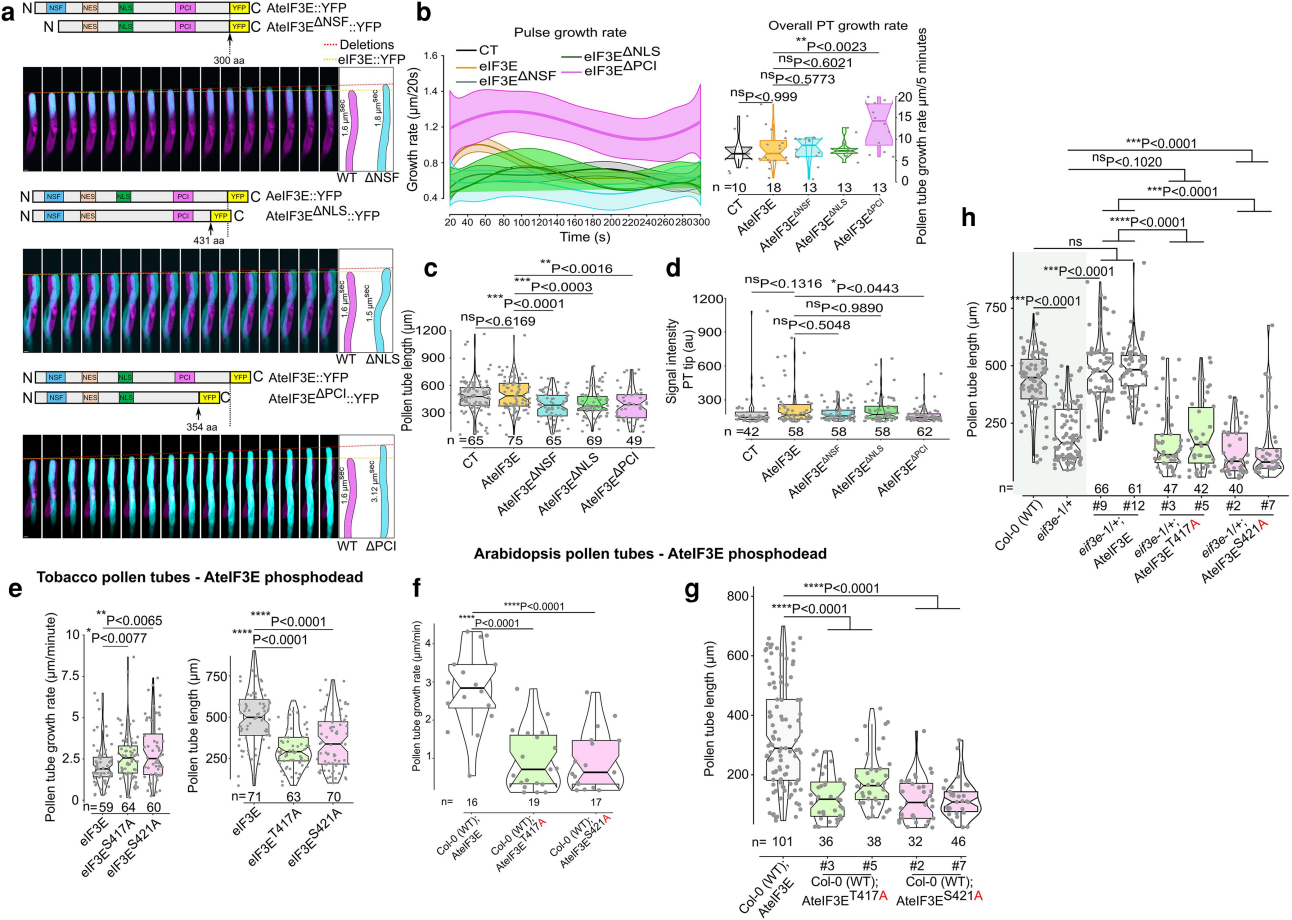
AteIF3E^ΔPCI^ domain deletion and phosphosites mutations alters pollen tube growth dynamics. (a) Schematic map of the respective constructs and time lapse images comparing the growth rate of in vitro grown tobacco pollen tubes expressing WT AteIF3E:YFP with eIF3E deletion variants AteIF3E^ΔNSF^, AteIF3E^ΔNLS^ and AteIF3E^ΔPCI^. Pollen tubes expressing mCherry:YFP alone were used as control (CT). Magenta colored are pollen tubes expressing full length wild type AteIF3E::YFP and cyan colored pollen tubes are those expressing respective deletion constructs. Dotted lines on the kymographs and their slope represent projection of the pollen tube growth rate. Equivalent kymograph for each time frame of each pollen tube is superimposed to provide direct visual comparison. Scale bars = 10 *μ*m. For real time live cell imaging supporting data, see Video S1. (b) Pulsating growth rate was measured from the equatorial distance from the pollen tube tip at 20 s intervals over 5 min period after 6 h post pollen germination. Overall average growth rate was measured after 6 h of pollen germination. The shaded areas around each line represent ± Sd of the mean pulse growth rate across replicates. Pulse growth rate (*µ*m/20 s) was measured as a function of time (s) for control (CT), wildtype eIF3E, and deletion mutants eIF3E^ΔNSF^, eIF3E^ΔNLS^, and eIF3E^ΔPCI^. (c, d) Pollen tube length were shorter in all AteIF3E domain deleted versions compared with full length AteIF3E, expressing tobacco pollen tubes. Right, protein accumulation in all deletion variants were comparable except eIF3E^ΔPCI^ which showed slightly reduced levels compared with full length AteIF3E::YFP. Signal intensity were measured from the tip of the growing pollen tube using standardized ROI measurement in ImageJ. Scale bar = 10 *μ*m. (e) Pollen tube growth rate and overall pollen tube length measurements following expression of AteIF3E^T417A^ and AteIF3E^S421A^ phosphodead in transient tobacco pollen tubes, 6 h after transformation. (f, g) Measurement of pollen tube growth rate and length from Arabidopsis transgenic stable lines expressing AteIF3E^T417A^ and AteIF3E^S421A^ in a wild type Col-0 background. Both AteIF3E-phosphodead mutants inhibited pollen tube growth, suggesting that AteIF3E^T417A^ and AteIF3E^S421A^ exert a dominant negative effect. (h) Complementation analysis of *eif3e*-1/+ Arabidopsis pollen tubes confirmed that both AteIF3E^T417A^ and AteIF3E^S421A^ -phosphodead variants are non-functional. Shaded areas represent controls. Notch-boxplots embedded in violin plot were prepared in R studio and statistics were applied in GraphPad Prism 9.1.1 using unpaired nonparametric *t*-test with Mann–Whitney *U* test to demonstrate the statistical difference of pollen tube growth rate and length from panel (b to h), where center line represent the median and the first and third quartiles indicate 25th and 75th percentiles, and the whiskers extend from minimum to maximum with 1.5 times the interquartile range from the 25th and 75th percentiles. Data points are shown as gray dots indicating individual pollen tube growth rate or length. Statistical comparisons performed with unpaired nonparametric *t*-test using the Mann–Whitney *U* test (GraphPad Prism 9.1.1). ^#^Number of independent lines evaluated. *N* = sample size (at least 3 replicates were performed for each individual data set).

We first analyzed the growth rate of AteIF3E^ΔNSF^::YFP, AteIF3E^ΔNLS^::YFP and AteIF3E^ΔPCI^::YFP in comparison to wild type full length AteIF3E::YFP. We observed that although AteIF3E^ΔNSF^::YFP and AteIF3E^ΔNLS^::YFP had no significant effect on the pollen tube growth rate, they notably reduced overall pollen tube length after 6 h of pollen growth (Fig. 7a to c and Video S1). In contrast, AteIF3E^ΔPCI^::YFP expression led to a dramatic increase in pollen tube growth rate, resulting in a doubling oscillatory intervals characterized by a distinct pause-and-go pattern compared with wild type AteIF3E::YFP (Fig. 7a, b and Video S1). Suprisingly, this rapid pulse-like growth was not sustained over long period, resulting in a final shorter pollen tube length than that of control AteIF3E::YFP expressing pollen tubes (Fig. 7c). Additionally, AteIF3E^ΔPCI^::YFP showed reduced protein expression in tobacco pollen tubes, whereas other 2 deletion variants were comparable to full length AteIF3E::YFP expression levels (Fig. 7d). Overall, all AteIF3E deletion variants exhibited a reduction in tobacco pollen tube length compared with full length AteIF3E::YFP. Similarly, in stable Arabidopsis transgenic lines expressing proUBQ10:AteIF3E^ΔNSF/ΔNLS/ΔPCI^::YFP, we observed a similar reduction in both pollen tube growth rate and overall pollen tube length, suggesting a common response between Arabidopsis and tobacco pollen tubes following deletion of conserved eIF3E domains (Figs. 7b, c and S3d to f).

To investigate the *in vivo* effects of phosphodead AteIF3E^T417A^ and AteIF3E^S421A^, we performed live-cell imaging of tobacco pollen tubes transiently expressing proUBQ10: AteIF3E^T417A^::YFP or proUBQ10: AteIF3E^S421A^::YFP and compared with wild type proUBQ10:AteIF3E::YFP expression. Both variants, AteIF3E^T417A^::YFP and AteIF3E^S421A^::YFP exhibited an accelerated growth rate similar to that observed with the deletion of PCI domain (Fig. 7e). This observation suggests that phosphorylation at Thr417 and Ser421 within the PCI domain may act as inhibitory modifications or as a modulator of pollen tube growth rate under standard growth conditions (Fig. 7e). However, like AteIF3E^ΔPCI^, the initial faster growth rate of AteIF3E^T417A^ and AteIF3E^S421A^ was not maintained, resulting in a shorter overall pollen tube length (Fig. 7e). Similar to observations in tobacco pollen tubes, the overexpression of proUBQ10: AteIF3E^T417A^::YFP and proUBQ10: AteIF3E^T421A^::YFP in wild type Col-0 Arabidopsis stable lines resulted in a reduced pollen tube growth after 4 h of germination similar to the effect of deleting the PCI domain in Arabidopsis stable lines (Fig. 7f, g). Both, proUBQ10: AteIF3E^T417A^::YFP and proUBQ10: AteIF3E^T421A^::YFP constructs also failed to complement the *eif3e*-1 pollen tube growth phenotype (Fig. 7h). Collectively, these results indicate that Thr417 and Ser421 are critical regulatory phosphosites within the PCI domain, playing an essential role in regulating eIF3E activity and facilitating proper pollen tube growth.

### eIF3E PCI domain phosphodead mutations T417A and S421A disrupt pollen tube membrane morphology

**Figure 8:**
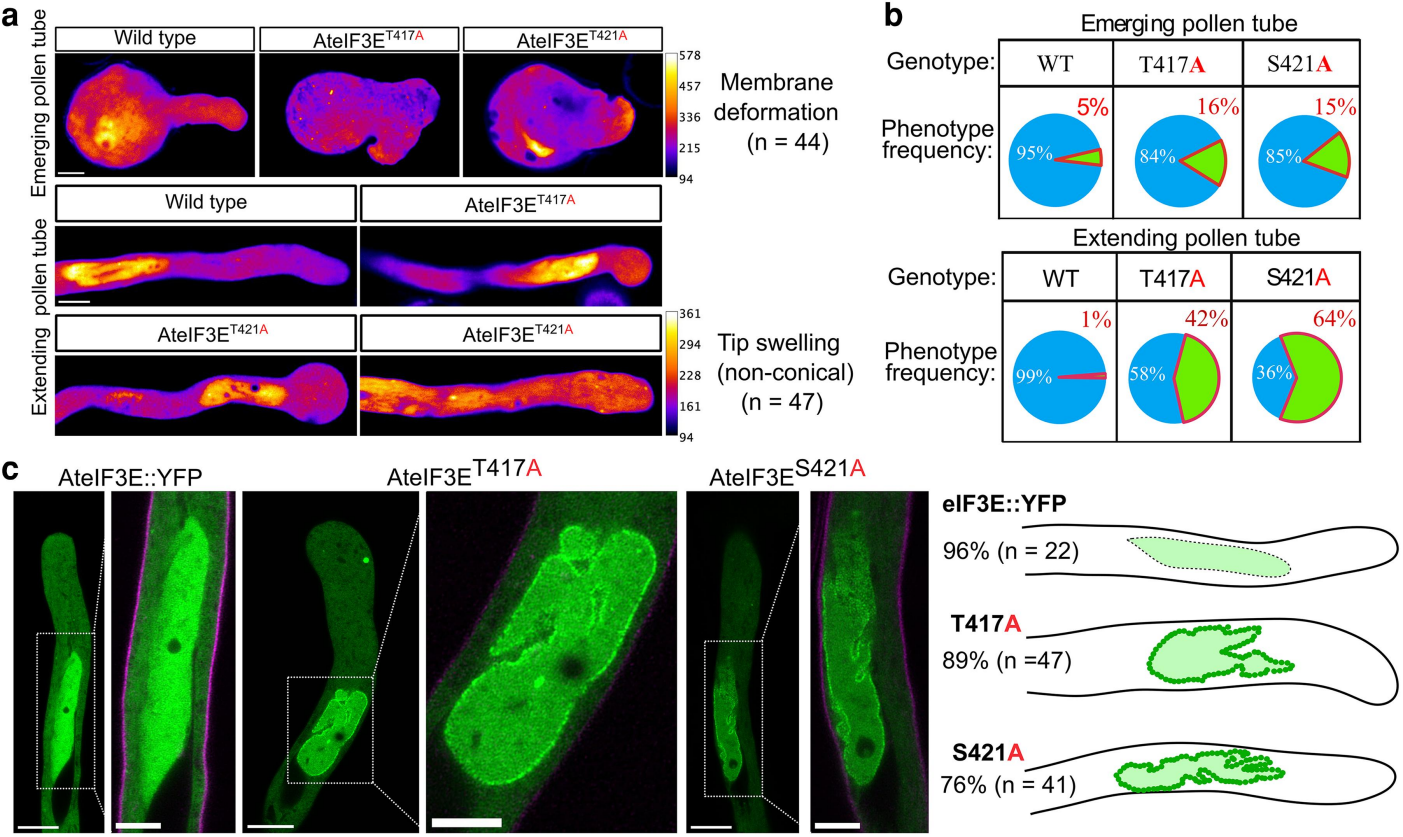
PCI domain phosphomutations in AteIF3E disrupt pollen tube membrane organization and vegetative nuclear envelope morphology. (a) Abnormal plasma membrane morphology of emerging and elongating tobacco pollen tubes expressing AteIF3E^T417A^ and AteIF3E^S421A^ phosphodead mutants variants. Images were fixed for color intensity code on ImageJ and the same pixel intensity range was set for all the images. Color intensity reflecting the differences in protein expression levels in wild type, AteIF3E^T417A^ and AteIF3E^S421A^ phosphodead mutants variants. Scale bar = 50 *μ*m set for all the images presented in this panel (b) Frequency of all the phenotypic classes subdivided between emerging pollen tube or extending pollen tubes subgroup. (c) Both phosphomutated eIF3E variants also induced defective vegetative cell nuclear membrane morphology in all pollen tubes expressing AteIF3E^T417A^::YFP or AteIF3E^S421A^::YFP phosphodead eIF3E variants. The phenotype was observed 6 h after tobacco pollen transformation. For real time live cell imaging supporting data, see Video S2. Scale bar = 10 and 5 *μ*m set for all the panel and the highlighted cropped images.

To further investigate the effects imposed by phosphodead AteIF3E^T417A^ and AteIF3E^S421A^, we examined the cellular morphology of tobacco pollen tubes expressing AteIF3E^T417A^::YFP and AteIF3E^S421A^::YFP. Strikingly, we observed severe plasma membrane deformations, including loss of polarity in emerging pollen tubes and membrane invaginations in extending pollen tubes, with a phenotype penetrance of 16% to 25% (*n* = 44) of transformed pollen tubes (Fig. 8a, b). In the case of fully elongated pollen tubes, 42% to 64% (*n* = 47) of the transformed pollen tubes displayed abnormal tip morphology, ranging from mild to extreme tip bulging or a loss of the characteristic conical shape tip seen in wild type tobacco pollen tubes (Fig. 8a, b). Moreover, cytoplasmic streaming and nuclear dynamic also appeared impacted upon AteIF3E phosphodead expression (Video S2). These morphological abnormalities indicate a disruption in polarity and uncoordinated pollen tube growth.

Furthermore, the expression of either AteIF3E^T417A^::YFP or AteIF3E^S421A^::YFP phosphodead eIF3E resulted in a high frequency (>80%, *n* = 89) of abnormal vegetative cell nuclear membrane morphology as early as 6 h post-transformation (Fig. 8c). These findings indicate that changes in the phosphorylation of eIF3E PCI-phosphosites Thr417 and Ser421 not only impaired pollen tube growth but also lead to substantial morphological distortion of both vegetative nuclear and pollen tube plasma membrane morphology. This underscores the essential role of post-translational modifications of eIF3E in preserving normal membrane structure.

## Discussion

Our current study elucidates the critical role of the translation initiation factor eIF3E in pollen tube growth and membrane morphology in Arabidopsis and tobacco. We show that eIF3E is part of the octameric eIF3 translation initiation complex and regulates the translation of a specific set of mRNAs, especially those enriched in MC1 (translational repressor) and MC2–MC3 (translational activators) *cis*-elements located within the mRNA CDSs (Fig. 5). These motifs co-exist on the same transcripts and act antagonistically to maintain translational equilibrium likely, by modulating ribosome pausing and release, impacting protein abundance (Figs. 5 and S7). Using RIP-seq and computational motif analysis, we have identified ∼191 mRNA targets, 95% of which harbor 5-mer MC motifs in their CDS. Many of these mRNAs encode proteins essential for pollen tube function, plasma membrane dynamics, and translation regulation (Fig. 5). The interplay of the MC motifs likely governs the on/off translation status by influencing 80S ribosomal complex behavior at the bound sites through associate-dissociate cycles or 80S overall stability.

Deleting the C-terminal PCI domain, prevented eIF3E dissociation from the 80S monosome post-initiation, leading to abnormal accumulation of eIF3E in heavy polysomes and impaired translation elongation (Fig. 4). Thus, the PCI domain not only facilitates integration of the eIF3E into the octameric complex but also likely mediates conformational changes necessary for the eIF3E dissociation post translation initiation. It will be important to test whether inefficient dissociation of eIF3E^ΔPCI^ also inhibit efficient dissociation of other remaining initiation subunits of the octameric complex and entry of elongation factors.

Furthermore, we have shown that 2 critical phosphorylation sites (Thr417 and Ser421) within the PCI domain act as functional “phosphoswitches.” Mutating these residues to phosphodead variants (AteIF3E^T417A^ and AteIF3E^S421A^) results in severe defects in pollen tube growth and membrane structure, mimicking the effects observed following PCI domain deletion (Fig. 8). These eIF3E-phosphodead variants fail to interact with eIF3L and thus likely unable to assemble into the octameric complex, failed to activate translation of MC2-containing mRNA reporters as well as unable to complement the *eif3e*-1 induced pollen tube growth phenotype (Figs. 2, 5 and 6 to 8). They also exhibited dominant-negative effects, leading to pollen tube growth arrest even in wild-type background (Fig. 7). Collectively, our findings support a model in which AteIF3E modulates translational equilibrium via the spatial arrangement and dynamic interplay of MC1–MC2–MC3 CDS *cis*-elements, as well as through its structural domains and phosphorylation status. This regulation is essential for balanced protein synthesis during the rapid and spatially dynamic growth of pollen tubes crucial for timely sperm cells delivery and fertilization in flowering plants.

### eIF3E associated mRNA targets and a putative mode of translational equilibrium

The regulation of translation is greatly affected by RNA regulatory elements and is crucial across a variety of organisms. In mice, the coexistence of 5′UTR translation inhibitory elements (TIEs), which inhibit general cap-dependent translation by blocking 43S scanning, and internal ribosome entry sites (IRES), that rely on ribosome heterogeneity on the recruitment of specific ribosomes for cap-independent translation initiation, establishes a translational equilibrium for distinct pools of mRNAs in a tissue-specific and temporal manner (Xue and Barna 2012). IRES elements typically promotes the translation of mRNAs related to stress-responsive and are highly translated, often in a tissue-specific context, by recruiting ribosomes when general translation is suppressed (Yang and Wang 2019; Son and Park 2023). The HOXA9 gene, which belongs to the Hox gene family, encodes mRNA that contains both TIE and IRES elements in its 5′UTR. Given the critical function of Hox gene in embryonic patterning, their expression is tightly regulated (Alexander et al. 2009). In *Hoxa9* IRES knockout mice, translation of HOXA9 in the neural tube and somite is suppressed under normal conditions, indicating that IRES elements are necessary for tissue-specific translation (Xue et al. 2015). Further investigation revealed that the translation of HOXA9 in these tissues is mediated by the specific recruitment of the ribosomal protein RPL38 at the IRES site, which likely counteracts TIE-induced translation inhibition (Xue et al. 2015). Similarly, knockdown of RPL38 reduces HOXA9 IRES activity, leading to the accumulation of IRES-knockout *Hoxa9* mRNAs in pre-polysomal fractions, suggesting a lack of association with actively translating ribosomal subunits (Xue et al. 2015).

**Figure 9:**
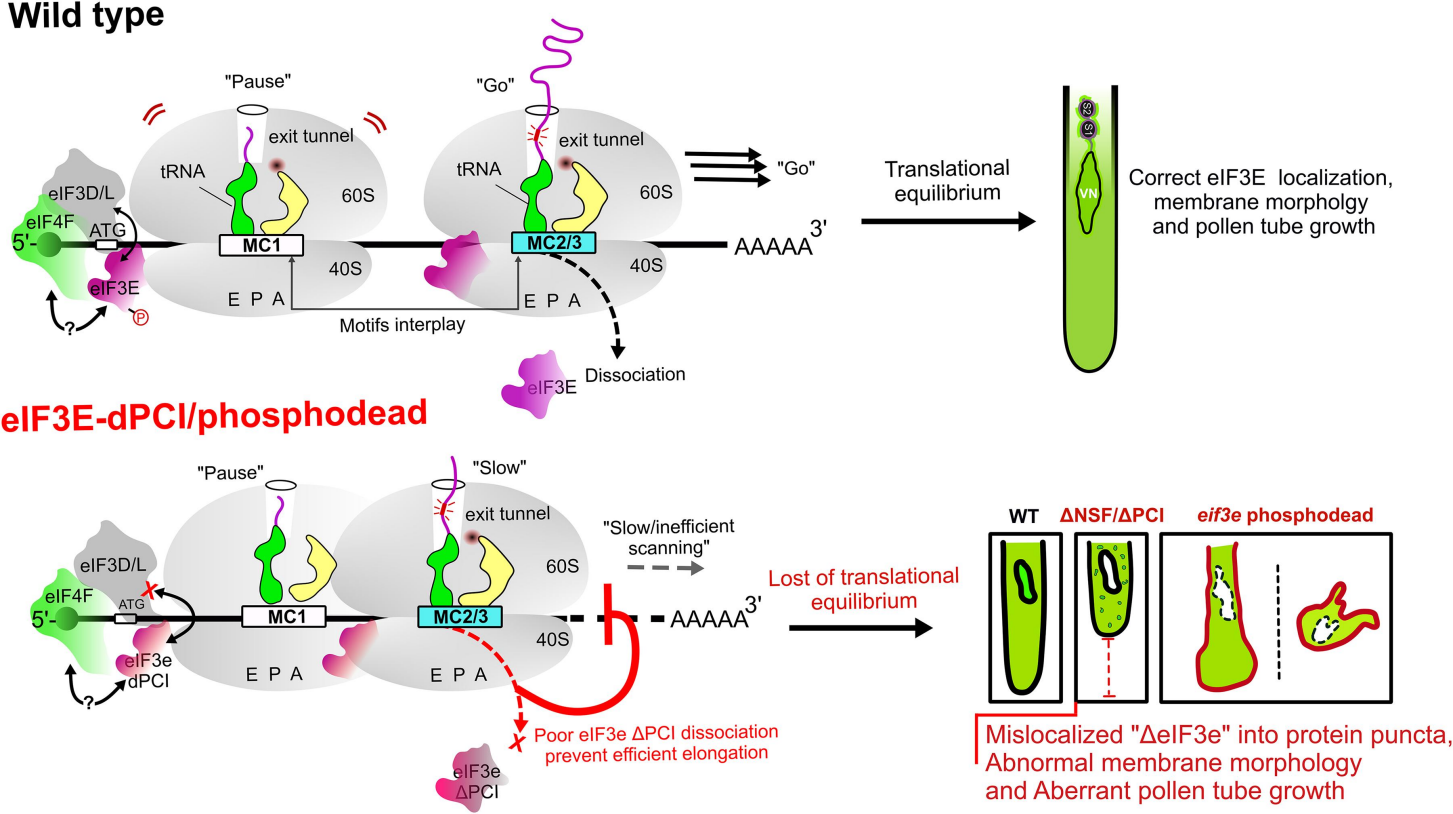
Proposed working model of eIF3E-mediated translational equilibrium for dynamic pollen tube growth. In wild-type growing pollen tubes, eIF3E binds mRNAs containing MC1–MC2–MC3 *cis*-elements to regulate their translation, thereby maintaining balanced protein synthesis necessary for proper membrane morphology and pollen tube growth. Disruption of eIF3E function, either through deletion of its PCI domain or mutation of its phosphorylation sites (eIF3E-phosphodead), impairs eIF3E interaction with eIF3D/L, causes eIF3E mislocalization, prevents eIF3E dissociation after translation initiation thereby inhibiting recruitment of translation elongation factors causing ribosome crowding, subsequently leading to defects in vegetative cell nuclear and plasma membrane organization as well as reduced pollen tube elongation. The observations marked with the question marks remain preliminary (Fig. S8) and require further experimental validation.

In our study, following AteIF3E::YFP immunoaffinity RIP-seq in tobacco pollen tubes, we identified a subset of core mRNA targets (182 target mRNAs representing 95% of the total identified targets) enriched with 5-mer *cis*-elements within their CDS (Fig. 5). Among these, the frequent motifs found are associated with mRNAs that encode members of the Hox gene family. We categorized these motifs as Motif CDS, MC1-repressor, MC2-activator, and MC3-activator. These motifs were tested using a transient assay in tobacco sporophytes (leaves) and pollen tubes, as well as in stable Arabidopsis pollen tubes, and their eIF3E-dependent activity was confirmed in the Arabidopsis *eif3e*-1 T-DNA mutant background (Figs. 5 and S7). The tested motifs represent top 3 of the 14 CDS motifs that we identified alongside additional 6 motifs at the 5′UTRs and 3 motifs at the 3′UTRs, respectively. The MC's motifs appeared in tandem within the same mRNA targets, with an average frequency of approximately 1:2.8 repressor:activator ratio (Fig. 5). Our analysis revealed that the MC1 repressor motif is most frequently located at the start of the CDS, followed by the activator motifs MC2 and MC3 (Fig. 5). This led us to speculate the mechanistic basis of how MC motifs could regulate translational on/off states within the CDS. Previous studies have shown that 5′UTR translational repressor *cis*-elements, such as TIE and uORF, or activators like IRES, can block translation by inhibiting 43S scanning or activate cap-independent translation by recruiting the pre-initiation complex internally (Xue and Barna 2012). Since the MC1 to MC3 motifs are located within the CDS, based on our results we propose a working model whereby MC1 and MC2/MC3 function antagonistically to impose pause and release of 80S ribosome during mRNA scanning, thereby modulating the translation rate of target mRNAs (Figs. 5, 9, and S7). CDS ribosome pausing can occur as a result of slow peptide bond formation involving asparagine, proline, glycine or cysteine residues at the ribosome P site or from delayed release of polylysine through the ribosomal exit tunnel at the E site, both of which reduce the translation rate of the associated mRNA (Buskirk and Green 2017; Krafczyk et al. 2021). MC1-motif indeed encode asparagine particularly at the beginning of CDS of the eIF3E-associated mRNA targets analyzed. The equilibrium between ribosome pause (repression) and release (activation) is likely to be determined by the frequency, positioning and proximity of the MC1 and MC2/MC3 *cis*-elements on the mRNA together with recruitment of *trans*-acting factors (Figs. 5 and 9). It will be critical to perform MC1-based Riboseq and disome-seq analysis on eIF3E target mRNA fragments to determine ribosome stalling/collision around the MC1 motif, as well as MC1-based Ribo-pulldown to identify co-translational *trans*-acting factors associated with the ribosome pause to further support the MC1–MC2/MC3 motifs mediated translational equilibrium (Fig. 5).

The biological importance of these motifs regulatory functions become more apparent when applying an enrichment cutoff of >2-fold (191 genes, 42.2%), these RNA targets primarily encode proteins associated with plasma membrane functions and signaling (37 genes, 33%), RNA biosynthesis and translation regulation (29 genes, 26%) and regulation of protein secretion (18 genes, 16%) (Table S2). Most of these annotated proteins are involved in pollen tube growth by regulating cell wall and plasma membrane status as well as signaling. These include receptor-like kinases (Galindo-Trigo et al. 2020; Gao et al. 2023; Xiang et al. 2024), GPI-anchor protein complexes such as COBRA-like protein 4 (Xu et al. 2024a, 2024b), PIG-5-like (Desnoyer et al. 2020), GPI17 (Desnoyer et al. 2020; Desnoyer and Palanivelu 2020), cellulose synthase-like D6 (Zhao et al. 2023), GDSL esterase-lipase (Yuan et al. 2020; Zhang et al. 2020), Fasciclin-like arabinogalactan protein 9 (Li et al. 2010a, 2010b; Lu et al. 2023), pectinacetylesterase, and regulators of phospholipids such as Phosphatidylinositol binding clathrin assembly protein (Zhao et al. 2010; Gou et al. 2012; Hocq et al. 2020), Phosphatidyl synthase HAD-superfamily (Burroughs et al. 2006), and Lipid-binding START (Schrick et al. 2004; Potocký et al. 2014).

In RNA biosynthesis and translation regulation, the eIF3E target mRNAs include ARGONAUTE 1 (Niedojadło et al. 2020), La-related protein-like RNA-binding protein (Billey et al. 2021; Guo et al. 2022), BRUNO-like RNA-binding protein (Lyon et al. 2009), several poly(A) RNA polymerases, including PAP25A and poly(A) polymerase 1 (Ramming et al. 2023), polyadenylate-binding proteins and cleavage and polyadenylation specificity factor subunit A (Liu and Manley 2024). Among the translation regulators targets, they include eIF2A/-2B (Byrne et al. 2012; Dutt et al. 2015; Hafidh et al. 2018), eRF1, RPS4, and RPL7 (Yan et al. 2016; Fakih et al. 2023), along with regulators of RNA splicing such as U3 small nucleolar RNA-associated protein 15 (Li et al. 2010a, 2010b), protein G (Ma et al. 1999), Arginine-serine-rich splicing factor (Jin 2022), HAT-helix PRP39 (Chang et al. 2022), Lsm5 (Golisz et al. 2013), and tRNA-splicing endonuclease (Trotta et al. 1997; Hopper and Nostramo 2019). In regulation of protein secretion, the targets include Rab 5-related proteins (Hao et al. 2023; Shi et al. 2023), AP3 complex subunit 2 (Feng et al. 2018), vacuolar sorting proteins (VSP4B, VSP24, VSP54, and VSP62) (Xiang et al. 2013), SEC14 (Xu et al. 2024a, 2024b), SEC61 (Mitterreiter et al. 2020), trafficking protein particle complex 10 (Wiese et al. 2024), BROX (BRO1-domain) (Shen et al. 2018), and Got1-like protein (Jia et al. 2018). All of these target mRNAs contain tandem repeats of the MC1–MC2–MC3 *cis*-elements within their CDS regions (Fig. 5; Table S2). In summary, the RIP-seq analysis revealed that AteIF3E mediates close translational control of core regulators involved in pollen tube growth and plasma membrane functions. This regulation likely occurs through a repressor-activator *cis*-element interplay that imposes the translational equilibrium required for pollen tube growth.

### Domains architecture of eIF3E

Systematic domain deletions and subsequent expression in both Arabidopsis and tobacco pollen tube, further revealed that the cytoplasmic-to-nuclear translocation of eIF3E protein relies on all eIF3E domains: the N superfamily domain, NLS, and the C-terminal PCI domain (Figs. 1 and 2). The dynamics of eIF3E nuclear translocation may also be influenced by interactions with other eIF3 subunits and nuclear transport factors (Watkins and Norbury 2004; Sha et al. 2009). In *S. pombe*, eIF3E can freely move to the nucleus in *eif3d*/*moe1* loss-of-function mutants, while eIF3D translocates to the nucleus in the absence of *eif3e*, indicating that the eIF3D–eIF3E interaction restricts their nuclear localization (Bandyopadhyay et al. 2002). Additionally, AteIF3E interacts with nuclear transport factors like importins-b Kap123p and Sal3p, potentially as a cargo (Yen and Chang 2000; Sha et al. 2009).

The PCI domain is known to facilitate the association of eIF3 core and non-core subunits within the octameric complex (Yahalom et al. 2001; Querol-Audi et al. 2013). Our study suggests that the PCI domain also plays a role in the dissociation of eIF3E from the translation initiation complex (Fig. 4). This dissociation is initiated by the hydrolysis of the eIF2-bound GTP ternary complex through eIF5b, along with the dissociation of eIF1A, potentially involving the C-terminal helices of the PCI domain (Thompson et al. 1977; Trachsel and Staehelin 1979; Thomas et al. 1980; Kolupaeva et al. 2005; Smith et al. 2016). Our findings support the gradual dissociation of wild type eIF3E from the 80S monosome, with minimal presence in heavy translating polysomes (Fig. 4). In contrast, the deletion of the PCI domain, the eIF3E^ΔPCI^ is notably trapped within the 80S monosomes, leading to its significant accumulation in both light and heavy polysomes (Fig. 4). These results suggest that the PCI domain contributes to the dissociation of eIF3E from the initiation complex. However, it remains unclear how the AteIF3E without the PCI domain can still associate with the 48S initiation complex. A plausible hypothesis is that the remaining N-terminal NSF domain might still interact with the 40S ribosomal protein eS9 as shown previously in budding yeast, thus prolonging the eIF3E^ΔPCI^ association with the polysomes (Paz-Aviram et al. 2008). Alternatively, the AteIF3E^ΔPCI^ could directly interact with the eIF4E cap-binding complex subunits (Chiluiza et al. 2011), bypassing the canonical interaction with the subunits of the octameric complex. A structural study of eIF3K and eIF3L further supported the need for the PCI domain for efficient eIF3 subunits engagement into the octameric complex. The assembly of subunits -3K and -3L are interdependent and occur only in the presence of eIF3H (Smith et al. 2016). Cryo-EM structural models of eIF3 revealed that subunits K and L are closely associated with the C-terminal helix of eIF3L forming the majority of contacts within the helical bundle (des Georges et al. 2015). The deletion of an N-terminal 42-amino-acid extension from eIF3K (eIF3K 43 to 237 aa) did not affect assembly, however, the additional removal of its C-terminal helix (eIF3K 43 to 217 aa) prevented its incorporation into eIF3, though it still allowed dimerization with eIF3L. Conversely, N-terminal truncations of eIF3L did not disrupt eIF3 complex assembly (eIF3L 69 to 475 aa or eIF3L 187 to 475 aa). Nonetheless, the integration of eIF3L 187 to 475 aa into eIF3 appears inefficient, as indicated by its poor expression and stability (Smith et al. 2016). Collectively, these deletion studies suggest that the efficient incorporation of subunits K and L into eIF3 requires both their C-terminal helices and a stable interaction between their PCI domains. In our experiment, our data suggest that the eIF3E PCI domain in combination with the extended C-terminal helices, also triggers conformational changes that promote the dissociation of AteIF3E from the 48S translation initiation complex. Additionally, prolonged association of AteIF3E^ΔPCI^ with polysomes appears to hinder the efficient recruitment of translation elongation factors eEF1A and eEF2 to the polysomal fractions, which likely interferes with the efficiency of translation elongation, potentially affecting key cellular processes (Fig. 4).

### Essential eIF3E phosphosites within the PCI domain

The core and non-core subunits of the octameric translation initiation complex in animals and yeast, such as eIF3A, -3B2, -3C, -3E, -3H, and eIF3J, are known to undergo phosphorylation at multiple sites (Dennis et al. 2009; Andaya et al. 2014; Shi et al. 2009; Mergner et al. 2020). Mutating conserved phosphosites in AteIF3E to create phosphodead variants of AteIF3E and their subsequent expression in transient tobacco pollen tubes, as well as by complementing Arabidopsis *eif3e*-1/+ pollen tube mutant phenotype, we identified 2 critical phosphoswitches, Thr417 and Ser421, within the PCI domain (Fig. 6). By combining biochemical approaches with live-cell imaging, we revealed that PCI Thr417 and Ser421 regulate pollen tube growth, vegetative cell nuclear membrane morphology as well as pollen tube plasma membrane morphology by inducing significant membrane deformation and invagination following AteIF3E^T417A^ or AteIF3E^S421A^ phosphodead overexpression (Figs. 6 to 8). The altered pollen tube growth phenotype resulted from phosphodead AteIF3E^T417A^ or AteIF3E^S421A^ expression, closely phenocopied the effects observed with the complete deletion of the PCI domain eIF3E^ΔPCI^ where the pollen tube growth rate initially tripled in transient tobacco pollen tubes (and partially in Arabidopsis eIF3E^ΔPCI^ stable lines) but ultimately resulted in shorter pollen tubes in both systems (Figs. 2, 7, and S3). To elaborate this initial inverse outcome in transient tobacco pollen tubes, it is plausible to hypothesize that the faster pollen tube growth rate is unsustainable as it requires accommodating an accelerated translation rate for constructing an effective pollen tube cell wall and adaptable faster overall metabolic rate required to support the faster growth rate. It remains intruiging, how a pollen tube can undergo a pulsatile growth exhibiting bursts of fast growth rate followed by pauses or slow growth with overall shorter pollen tube length?. Pollen tubes grow in pulses regulated by an oscillatory network in which cytosolic Ca^2+^, actin dynamics, cell wall mechanics and turgor pressure interact with defined phase lags relative to peak elongation (Benkert et al. 1997; Robinson et al. 2002). These coordinated feedbacks can enhance instantaneous growth rates yet increase the frequency of pauses, growth arrests or even pollen tube rupture, so pollen tubes may grow faster but ultimately achieve shorter final lengths (Feijó et al. 2001; McKenna et al. 2009; Hepler et al. 2013; Damineli et al. 2017). For instance, oscillations in tip-focused Ca²⁺ gradients promote exocyst-mediated vesicle fusion and actin regulation during fast growth, but sustained high Ca^2+^ depolymerizes actin and stiffens the wall, preventing pollen tube elongation (Hepler et al. 2013; Michard et al. 2008; Rojas et al. 2011). Similarly, the subapical actin fringe oscillates, with transient polymerization enhancing vesicle delivery and faster growth rates, whereas depolymerization during Ca^2+^ peaks prolongs non-growing phases, shortening the effective growth cycle (Hepler et al. 2013; Winship et al. 2021). The cell wall status also fluctuate, as newly secreted, highly methyl-esterified pectin transiently softens the pollen tube apex while increase PME activity and Ca^2+^ cross-linking re-stiffen the cell wall. Larger oscillations in wall extensibility thus generate rapid expansion during soft phases but long stiff phases or even bursting, limits cumulative length resulting in overall shorter pollen tube length (Bosch and Hepler 2005; McKenna et al. 2009; Zerzour et al. 2009). Although the effect of turgor pressure has only been reported from limited experimental and modeling evidence, transient increases in effective pressure relative to wall softness can accelerate growth by increasing exocytosis of cell wall material, but stiffening phases and overpressure-induced rupture constrain the average growth and shorten net pollen tube length (McKenna et al. 2009; Dumais 2021).

In comparison, Arabidopsis stable lines expressing eIF3E^ΔPCI^ construct also showed higher growth rate compared with plants expressing eIF3E^ΔNSF^or eIF3E^ΔNLS^, partially phenocopying the dominant negative effect of eIF3E^ΔPCI^ expression observed in transient tobacco pollen tubes (Figs. 7 and S3). We reason that this difference response may arise from differences in the temporal dynamics of pollen germination and elongation by the 2 model systems. In the transient tobacco assay, pollen tube length was assessed as a fixed end-point measurement (6 h post-germination) when the early expression of eIF3E^ΔPCI^ in transformed tobacco pollen appears to initially suppresses pollen germination relative to pollen expressing full-length eIF3E:YFP or the eIF3E^ΔNSF^/eIF3E^ΔNLS^ variants, most likely as a consequence of the dominant-negative cytotoxic activity of eIF3E^ΔPCI^. In the subsequent post-germination growth, the eIF3E^ΔPCI^ transiently expressing pollen tubes undergo partial adaptation, potentially through progressive transcriptional downregulation of eIF3E^ΔPCI^ or its proteolytic turnover, thereby alleviating the toxicity and compensate the delayed germination with pulsatile elongation. This could account for the paradoxical observation of overall shorter pollen tube length, yet faster growth rate at the 6 h time point measurement (Figs. 7 and S3).

By contrast, in stably transformed Arabidopsis lines, continuous accumulation of eIF3E^ΔPCI^ throughout pollen development perturbs pollen maturation resulting in mature pollen phenotype (Fig. S3). Consequently, germination efficiency and growth rate is substantially compromised from the onset in eIF3E^ΔPCI^ stable lines pollen germination. This developmental impairment likely underlies the pronounced defects in pollen tube emergence and elongation in Arabidopsis eIF3E^ΔPCI^ stable lines (Fig. S3).

A similar correlation between rapid cell elongation and reduced overall length has been observed for instance in auxin receptor IAA17/AXR3 overexpression, which affects cell wall integrity in Arabidopsis roots leading to root growth arrest (Kubalová et al. 2024). To elaborate the molecular impact of the AteIF3E^T417A^ or AteIF3E^S421A^ loss-of-function similar to the effect observed following eIF3E^ΔPCI^ deletion, we demonstrated that AteIF3E^S421A^ fails to interact with eIF3L, suggesting its inability to assemble into an octameric complex. Additionally, AteIF3E^S421A^ phosphodead is incapable of activating the MC2-mRNA::YFP reporter translation and that it fails to complement the Arabidopsis *eif3e*-1/+ T-DNA mutant (Figs. 6 and 7). Both AteIF3E^T417A^ and AteIF3E^S421A^ exhibited a dominant-negative effect by inhibiting pollen tube growth in wild type Col-0 background expressing native eIF3E in both stable Arabidopsis and in transient tobacco pollen tubes (Fig. 7). These findings underscore the significance of eIF3E phosphoswitches in activating translation of its mRNAs targets during pollen tube growth (Fig. 9).

In conclusion, our study provides a molecular insight into the mechanistic function of AteIF3E in achieving the translational equilibrium of target mRNAs regulating pollen tube growth and membrane morphology, crucial for plant fertility (Fig. 9). We propose that the PCI domain and its phosphoswitches orchestrate AteIF3E interaction with the octameric complex and ribosomal subunits, balancing translation repression and activation of mRNAs containing MC1–MC2–MC3 *cis*-element motifs (Figs. 3, 5, 6, and 9). The tandem occurrence of the translational repressor element MC1 and the enhancer elements MC2 and MC3 on the same target mRNA provides a translational equilibrium for target proteins (Fig. 9). Outstanding questions remain regarding the exact mechanism by which eIF3E-dependent MC1–MC2–MC3 *cis*-elements achieve the on/off translation status of specific mRNAs and whether other MC motifs-dependent *trans*-acting factors work alongside AteIF3E to regulate the translational equilibrium of eIF3E co-immunoprecipitated mRNAs in pollen tubes. A possible hypothesis could be that eIF3E, in coordination with other MC motifs *trans*-acting factors, regulates ribosomal subunits stability (pause-release) at the bound MC1–MC2/MC3 *cis*-elements sites, thereby modulating 80S ribosome scanning and translation efficiency. This regulation of proteome homeostasis plays a crucial role in maintaining the correct growth rate, adequate membrane synthesis, and morphology, which is essential for proper pollen tube growth, sperm cell delivery, and successful fertilization.

## Materials and methods

### Plant material and growth conditions

Mutant (*eif3e*-1) and complemented transgenic lines are in *A. thaliana* ecotype Columbia-0 (Col-0) background. The *eif3e*-1 (SALK_121004; Yahalom et al. 2008; Roy et al. 2011) mutant line was ordered from the NASC Arabidopsis stock center. The floral dip technique was used to generate stable complemented lines (Clough and Bent 1998). For *in vitro* culture, seeds were first surface sterilized with 100% ethanol for 1 min and followed by 20% bleach for 5 min with repetitive washes with water (5 times) and sown in Murashige and Skoog (MS) medium. Arabidopsis plants were cultivated in 22% and 60% humidity in a phytotron with continuous cycles of 16-h light/8-h dark conditions. The *eif3e*-1 was genotyped using a mixed combination of gene-specific primers, and T-DNA primer (LBb1.3). Genotyping primers are listed in Table S3. For extended methods, see Supplementary Methods file.

### RIP-seq preparation and analysis

RNA co-IP was performed as previously described (Billey et al. 2021) using the eIF3E:YFP transformed pollen tubes or untransformed pollen tubes as a negative control. Briefly, tissue powder from PTs were incubated in 1 mL of lysis buffer (200 mm Tris, pH 9.0, 110 mm potassium acetate, 0.5% Triton X-100, 0.1% Tween 20, 5 mm DTT, and 1.5% protease inhibitor, 0.375% formaldehyde). The mixture was incubated on ice for 10 min. The crosslinking reaction was quenched by adding glycine at a final concentration of 200 mm for 5 min. After cell debris removal by centrifugation (10 min at 16,000 × *g*, 4 °C), the supernatant was incubated with 25 *μ*L of GFP-trap beads (ChromoTek) for 1.5 h at 4 °C under rotation. Beads were washed 5 times with 0.75 mL of lysis buffer. RNA Elution was performed with 200 *μ*L of 8 M guanidium for 5 min on ice and precipitated overnight with 300 *μ*L of 100% ethanol before RNA extraction using Monarch Total RNA Miniprep Kit. RNA library preparation was performed using the NEBNext Ultra II RNA Library Prep Kit according to the manufacturer's instructions. Libraries were multiplexed and sequenced on a NextSeq 550 instrument in SR75.

Raw reads were trimmed using Trimmomatic v0.39 (Bolger et al. 2014) to eliminate adapters and low-quality sequences. The trimmed reads were then aligned to the *N. tabacum* genome obtained from the Solgenomics project (Nitab-v4.5, Edwards et al. 2017), along with the corresponding gtf file annotations, using Hisat2 v2.2.1 (Kim et al. 2019) with default parameters. Only uniquely mapped reads were retained by utilizing samtools v1.13 with the “-q 10” option (Li et al. 2009). Subsequently, read counts were determined using htseq-count v1.99.2 in “union” mode (Anders et al. 2015). Motif analysis was executed using the HOMER software's findMotifs function. Gene annotations from Nitab v4.5 (5′UTR, 3′UTR, CDS) were utilized to define the respective background for each dataset.

### RNA labeling and RNA immunoprecipitation for RIP-seq-PCR

The selected AteIF3E RIP-seq identified target RNAs, including {U3 small nucleolar RNA-associated protein 15 homolog (U3 snRNAa15), ribosomal L9-like protein (rPL6-SH3), aminocyclopropane-1- carboxylate synthase (Prop1AT), GPI ethanolamine phosphate transferase (EthAP) as well as motifs (MC1, MC2, and MC3) and scramble variants (Sc1, Sc2, and Sc3)} were amplified from tobacco pollen or pollen tube using Phusion High-Fidelity DNA Polymerase (Thermo Fisher Scientific, Waltham, MA, USA). PCR products were ligated into pDONOR-P1-P2 to generate respective entry clones. Similarly, an aptamer PP7 or YFP fluorophore were cloned into pDONOR-P2-P3 to create the entry clone. UBQ10 (ubiquitin 10) promoter was subcloned into pDONOR-P4-P1. The over-expression constructs of respective RNAs/motifs target labeled with PP7 aptamer/YFP were prepared by recombining the above generated entry modules into expression cassette pB7m34GW,0 through Multisite Gateway LR Clonase II Enzyme mix (Thermo Fisher Scientific). Tagged RNAs with PP7 and all individual motifs (MC1, MC2, and MC3) scramble (Sc1, Sc2, and Sc3) were co-infiltrated either with AteIF3E-mCherry and AteIF3E::YFP, AteIF3E^ΔPCI^::Flag, AteIF3E^S421A^::Flag and mCherry:YFP as control into *N. benthamiana* leaves in a ratio of 3:1 and 1:1. Images were taken by super-resolution confocal microscopy LSM900 in Airyscan module with standardized algorithms and downstream analysis was performed either on ImageJ plug-ins or Zen blue software package (Zeiss). For RIP-seq PCR, total protein was isolated from the co-infiltrated leaves from PP7 tagged RNA targets and motifs. Protein pull-down was performed using anti FLAG M2 magnetic beads (Sigma-Aldrich) and GFP-trap beads (ChromoTek). The RNA elution was done according to the method described in the RIP-seq preparation and analysis section with slight modifications. After, overnight precipitation (−20 °C), the RNAs were pelleted by centrifugation at maximum speed (14,000 rpm) for 45 min. The RNA pellet was washed with 750 *μ*L of 80% ethanol at full speed for 5 min. After the air drying, the pellet was resuspended in 10 *μ*L of DNase/RNase free water. DNase treatment was done with RQ1 RNase-free DNase I (Promega, MD, USA) and the cDNA library were prepared using recombinant M-MLV reverse transcriptase (Promega). SemiRT-PCR was finalized with gene specific and YFP primers for RNA targets and the motifs (listed in Table S3).

### Polysome profiling

AteIF3E:YFP and AteIF3E^ΔPCI^:YFP were transiently expressed in *N. benthamiana* leaves (Billey et al. 2021). Two days post infiltration leaves were collected and processed for polysome analysis according to Mustroph et al. (2009a, 2009b) and Hafidh et al. (2018). Briefly, tobacco leaves were collected, weighed and samples were quickly frozen in liquid nitrogen, ground to powder in equal weight input and resuspended in 1:3 ratio of polysome extraction buffer (200 mm Tris-HCl pH 9.0, 200 mm KCl, 36 mm MgCl_2_, 25 mm EGTA, 1% Triton X-100, 1% Tween 20, 2% polyoxyethylene cholate, 5 mm DTT, and 25 *µ*g/mL cycloheximide, 1% Brij-35, 2% deoxycholic acid). Equal volume solution was then loaded on top of 10% to 45% sucrose density gradient prepared using Biocomp GRADIENT MASTER 108 in a 13.5 mL pierceable polypropelene ultracentrifuge tubes (Beckmann Coulter) 24 h before and stored overnight at 4 °C. The samples were centrifuged using SW41Ti rotor at *rcf*_1_ = 110,000 × *g*) for 3 h at 4 °C. The polysome profile was recorded with the analog and CALIBRICK digital detector with Clarity software chart recorders using Teledyne gradient fractionator and Foxy R1 fraction collector connected to a UA-5 detector (Teledyne ISCO, USA). Six biological replicates were collected. After fractionations (40S, 60S, 80S, and polysomes), samples were collected and precipitated in 2 volumes of 100% ethanol overnight at −20 °C. Protein samples were equally loaded and separated on 10% resolving SDS–PAGE (Miniprotean, BioRad). Subsequently, proteins were transferred to the PVDF membrane (0.45 *μ*m, Thermo Scientific, USA), and washed 3 times with 1× TBST (1× TBS-0.1% tween 20) followed by membrane blocking (5% fat-free milk in 1× TBST) for 30 min. The membrane was washed 3 times with 1× TBST, and incubated in primary antibody rabbit anti-GFP/anti-RFP (Proteintech) with 1:10,000 dilution at 4 °C overnight. The membrane was washed 3 times in 1× TBST and incubated for an hour with a secondary anti-rabbit conjugated with Horseradish peroxidase (Agrisera). The Membrane was developed with chemiluminescence solutions (Agrisera ECL SuperBright western blot reagents) and exposed in CCD G:BOX F3-gel imaging for Chemiluminescence (Syngene). The intensity of the bands was measured in Plot Lanes using ImageJ.

### AlphaFold 3-ColabFold-ChimeraX PPI predictions

Protein sequences for eIF3 complex subunits were downloaded from the UniProt and TAIR and subsequently uploaded into the AlphaFold3 server (https://alphafoldserver.com) as a molecule type in a JSON format (Abramson et al. 2024). In a similar manner, we used ColabFold MMseqs2 with AlphaFold2 or RoseTTAFold AlphaFold2/integrated in ChimeraX (Goddard et al. 2018; Pettersen et al. 2021; Meng et al. 2023). The GPU usage was enabled by default (https://alphafold.colabfold.com). All predictions steps were executed continuously by every cell in the notebook. By default, ColabFold generates 5 structure models and the plots for sequence coverage and predicted aligned error (PAE). The most accurate predicted structures from both AlphaFold and ColabFold were modified in ChimeraX and the contact points for PPi were determined by generating the AlphaFold error plot and employing command (alphafold/A to /B), where A and B denote the bait and pray proteins. The protein confidence for the PPi were accessed by the interface predicted TM-score (ipTM) which measure the the accuracy of the predicted relative positions of the subunits forming the protein–protein complex and to infer the structural accuracy, we used the predicted template modeling (pTM) and per-residue confidence metric (pLDDT) and model confidence scores. The average or best ipTM and pTM scores from those models were used in our analysis. The model confidence scores were calculated by equating the ipTM and pTM score in the equation (0.8 × ipTM + 0.2 × pTM).

### Recombinant protein expression, IMAC affinity purification and immunoprecipitation from tobacco pollen tubes

BL21-Codon Plus (DE3)-RIPL *Escherichia coli* strains (Agilent Technologies) were used for recombinant eIF3E protein expression. Transformation of control (His:MBP) tag only, and NteIF3E-His:MBP in BL21-Codon Plus (DE3)-RIPL was done according to manufacturer instructions. Transformed BL21 cells were inoculated in a 5 mL Luria broth (LB) medium comprising appropriate antibiotic selections for the expression constructs. Cultures were grown overnight in a shaker at 37 °C at 180 rpm. Subsequently, 500 *μ*L of the culture was inoculated into a 100 mL flask containing 10 mL LB medium with antibiotic and incubated in a shaker at 37 °C with 250 rpm. Optical density was monitored until reached 0.8 OD, then recombinant protein expression was induced with 0.4 mm IPTG (ICN Biomedicals) onto the culture. The inoculated culture was kept overnight and cells were harvested by centrifugation for 10 min at 4 °C at 4,000 rpm, and pellets were kept at −80 °C until further use.

Total protein from soluble fractions was isolated by grounding pellet in liquid nitrogen with 250 *μ*L of rapid immunoprecipitation assay buffer (RIPA: 10 mm Tris/HCl pH 7.5 [MP Biomedicals], 150 mm NaCl [Sigma], 0.5 mm EDTA [Duchefa], 0.5% NP-40 [Sigma], pierce protease inhibitor [Thermo Fisher Scientific, 1 tablet per 30 mL buffer], 10 mm phenylmethylsulphonyl fluoride [Sigma]). Grounded samples were incubated on ice for 20 min. Followed by centrifugation at 4 °C at maximum speed for 10 min. The supernatant (sample) was transferred into 1.5 mL eppendorf tubes. One hundred microliters of Ni-NTA agarose beads (Qiagen) were equilibrated with 500 *μ*L of wash buffer (10 mm Tris/HCl pH 7.5 [MP Biomedicals], 150 mm NaCl [Sigma], 0.5 mm EDTA [Duchefa]). After washing, beads were transferred onto eppendorf tubes containing the samples. 10 *μ*L of the supernatant was kept as an input control. Samples were incubated for 1 h on a rotating wheel at 4 °C. Post incubation, samples were centrifuged at 2,500 × *g* for 30 s at 4 °C. The beads were washed 3 times with 500 *μ*L of 20 mm imidazole (Sigma), and centrifuged at 2,500 × *g* for 30 s after each wash. His-tag proteins were eluted with 50 *μ*L of 500 mm imidazole. Five microliters of pull-down samples were taken for western blot analysis to assess the expression of eIF3E protein with anti-His (Agrisera) and anti-eIF3E custom antibody (Moravian-Biotech).

For *in vitro* immunoprecipitation, Ni-NTA purified recombinant NteIF3E::His:MBP (45 *μ*L) or tag only His:MBP control (45 *μ*L) were dialyzed with 2 mL PBS buffer (pH7.0) using Amicon Ultra dialysis filter with 3 kDa cut off (Merck Millipore). Next, the dialyzed 45 *μ*L samples of NteIF3E::His:MBP or control His:MBP tag only were incubated with 500 *μ*L of pollen and PT extracts (isolated using the standard RIPA buffer as mentioned above) in 2 mL eppendorf tube. Ni-NTA agarose beads were equilibrated with washing buffer (Tris/HCl pH 7.5, 150 mm NaCl, and 0.5 mm EDTA) and subsequently transferred (100 *μ*L) to 2 mL eppendorf containing either recombinant NteIF3E::His:MBP with pollen and PT extracts or control His:MBP with pollen and PT extracts mixture. The mixtures were then incubated on a rotating wheel for 2 h at 4 °C to pull-down NteIF3E interacting partners from pollen and pollen tube. After incubation, beads were washed 3 times with 500 *μ*L of wash buffer and centrifuged at 2,500 × *g* for 30 s to collect the beads. After the final wash, beads were collected in 30 *μ*L of wash buffer, and 5 *μ*L beads were used to run SDS PAGE stained with Coomassie CBB G-250. The remaining beads were subjected to LC-MS/MS proteome analysis following trypsin digest.

### LC-MS/MS analysis

Samples were digested by trypsin O/N at 37 °C and analyzed using a liquid chromatography system Vanquish (Thermo Scientific) connected to the timsTOF SCP mass spectrometer equipped with Captive spray (Bruker Daltonics). The Mass spectrometer was operated in a positive data-dependent mode. One microliter of peptide mixture was injected by autosampler on the C18 trap column (Pepmap Neo C18 5 *µ*m, 0.3 × 5 mm, Thermo Scientific). After trapping, peptides were eluted from the trap column and separated on a C18 column (Pepsep C18 150 × 0.15 mm, 1.5 *µ*m, Bruker Daltonics) by a linear 35 min water−acetonitrile gradient from 5% (v/v) to 35% (v/v) acetonitrile at a flow rate of 1.5 *μ*L/min. The trap and analytical columns were both heated to 50 °C. Parameters from the standard proteomics PASEF method were used to set timsTOF SCP. The target intensity per individual PASEF precursor was set to 20,000, and the intensity threshold was set to 1,500. The scan range was set between 0.6 and 1.6 V s/cm^2^ with a ramp time of 100 ms. The number of PASEF MS/MS scans was 10. Precursor ions in the m/z range between 100 and 1,700 with charge states ≥2+ and ≤6+ were selected for fragmentation. The active exclusion was enable for 0.4 min. The raw data were processed by PeaksStudio 10.0 software (Bioinformatics Solutions, Canada). The search parameters were set as follows: enzyme—trypsin (semispecific), carbamidomethylation as a fixed modification, oxidation of methionine and acetylation of protein N-terminus as variable modifications. The data were searched against the *A. thaliana* protein database.

### Yeast 2 hybrid

For Y2H, AteIF3E, AteIF3A, AteIF3D1, and AteIF3L1 were amplified from Arabidopsis inflorescence cDNA by 2-step PCR with stop codon and subsequently ligated in Gateway donor vectors (pDONOR221-P1-P2) to generates entry clones using BP Clonase II enzyme mix. The above generated entry module then recombined into the yeast expression vectors pDEST32 and pDEST22 plasmid and vice versa to create the bait and pray plasmid clones. The yeast strain MaV203 was used to perform Y2H experiments. Three control plasmids based on the interaction of Krev1 (a known member of the Ras family of GTP binding proteins) with RalGDS (Ral guanine nucleotide dissociator stimulator protein, Herrmann et al. 1996; Serebriiskii et al. 1999). Interaction of RalGDS-WT with Krev1, was considered as strong positive control. The mutant variants of RalGDS-WT, termed m1, that affect interaction with Krev1 were used as weak positive control. MaV203 competent cells were co-transformed with bait and prey constructs and selected on synthetic media lacking leucine and tryptophan and supplemented with histidine/Uracil plates (SC-Leu-Trp + His + Ura). Three independent yeast colonies were streaked onto new media containing (SC-Leu-Trp + His + Ura). After the growth, colonies were dissolved in 250 *μ*L water and spotted on synthetic media excluding leucine, tryptophan, histidine, and on 15 mm 3-AT (3-amino-1,2,4-triazole) (SC-Leu-Trphistidine + Uracil + 15 mm 3-AT) for positive interactions. For negative selection, yeast colonies were spotted on media containing (SC-Leu-Trp + histidine + Uracil + 0.2%) 5FOA (5-fluoroorotic acid). The experiments were repeated 3 times.

### FRET cloning and analysis

For FRET experiments, AteIF3E, AteIF3A, AteIF3G, AteIF3D1, and AteIF3L1 were amplified from Arabidopsis inflorescence cDNA by 2-step PCR. Amplified PCR products were ligated using the BP Clonase II enzyme mix into Gateway donor vectors (pDONOR221-P3-P2, and pDONOR-P1-P4) to generates entry clones. For AteIF3E and NteIF3E domain deletions, we first amplified CDS sequence from pollen and pollen tube of Arabidopsis and tobacco using 2 step PCR method and then ligated into the pDONR221-P1-P2 and generated the entry module. Then the entry clones were used to obtain delete the Nsuperfamily (At/NteIF3E^ΔNSF^), NLS (At/NteIF3E^ΔNLS^) and PCI (At/NteIF3E^ΔPCI^), respectively by employing the Infusion Contech TaKaRa kit. Subsequently, above created entry clones were used in LR Clonase II Plus (Thermo Fisher Scientific) reactions with pFRETtv-2in1-CC (Hecker et al. 2015) and pB7m34GW,0 via Multisite Gateway LR Clonase II Enzyme mix (Thermo Fisher Scientific) to obtain the expression construct comprising 2 protein-coding genes tagged C-terminally with mTurquoise (donor fluorophore-eIF3E) and mVenus (acceptor fluorophore- eIF3A, eIF3D1, and eIF3L1) and deletion variants of AteIF3E and NteIF3E under overexpression promoter either LAT52 or UBQ10 (ubiquitin 10) and C-terminal protein fused with YFP fluorophore. Confirmed clones were used to transfect the *N. benthamiana* pavement cells for FRET, polysome profiling and transient transformation in tobacco pollen tube or generation of Arabidopsis stable lines. FRET data collection was conducted with ZEISS LSM880 using 405 and 488 nm lasers for excitation and 488 nm laser for acceptor photobleaching at 100% power. FRET events were recorded with ZEN blue software and subsequent analysis was conducted following the acceptor photobleaching method. Briefly, FRET efficiency (E) was calculated according to the equation below, where *I*_A_ and *I*_D_ denoted the background-subtracted fluorescence intensities of the acceptor and the donor, respectively (*β* is the leakage of mTurquoise signal into YFP channel; γ is the ratio of quantum efficiencies of the 2 channels) (E = *I*_A_-β*I*_D_ / *I*_A_+γ*I*_D_). All primers used for cloning are listed in Table S3.

### Statistical evaluation

Box–Whisker and notch-violin plots were generated using R studio (Version 2022.12.1 + 364 (2022.12.1 + 364) and GraphPad Prism 9.1.1. Unpaired nonparametric *t*-test using the Mann–Whitney *U* test and *χ*^2^ test were employed to perform statistical analysis using GraphPad Prism 9.1.1 version (223).

### Accession numbers

All the listed gene sequence data can be found in the TAIR/NCBI/Sol genomics network under the following accession numbers; eIF3E (AT3G57290), KAKU4 (AT4G31430), PABP5 (AT1G71770), eIF3A (AT4G11420), eIF3C (AT3G56150), eIF3D1 (AT4G20980), eIF3L1 (AT5G25757), eIF3G1 (AT3G11400), RBP47A (AT1G49600), DCP5 (AT1G26110), eIF4E1 (AT4G18040), eIF4G (AT3G60240).

## Supplementary Material

koag005_Supplementary_Data

## Data Availability

RIP-seq data was deposited at NCBI under the project number PRJNA1049184. LCMS/MS data is available at ProteomeXchange (PRIDE) under accession PXD048337.

## Dive Curated Terms

The following phenotypic, genotypic, and functional terms are of significance to the work described in this paper:

BiP1 Gramene: AT5G28540
BiP1 Araport: AT5G28540

PRP39 Gramene: AT1G04080
PRP39 Araport: AT1G04080

SEC14 Gramene: AT4G39180
SEC14 Araport: AT4G39180

WRKY55 Gramene: AT2G40740
WRKY55 Araport: AT2G40740

EIF3e Gramene: AT3G57290
EIF3e Araport: AT3G57290

COP9 Gramene: AT4G14110
COP9 Araport: AT4G14110

HSP70 Gramene: AT3G12580
HSP70 Araport: AT3G12580

RPS6 Gramene: AT5G46470
RPS6 Araport: AT5G46470

UBQ10 Gramene: AT4G05320
UBQ10 Araport: AT4G05320
