# Correction to: Domain architecture of plant eukaryotic translation initiation factor 3 subunit E governs interaction with translational *cis*-elements to regulate pollen tube growth

**DOI:** 10.1093/plcell/koag084

**Published:** 2026-04-01

This is a correction to: V Kumar, R Merret, MC Carpentier, D Honys, S Hafidh, Domain architecture of plant eukaryotic translation initiation factor 3 subunit E governs interaction with translational cis-elements to regulate pollen tube growth, *The Plant Cell*, Volume 38, Issue 2, February 2026, koag005, https://doi.org/10.1093/plcell/koag005

Following article publication, the authors noted an error in Figure 4E; along the graph's x-axis, the far-right label should have been “polysome”, not “monosome”. The authors have since updated the figure, as well as the names of both the third co-author and the ninth person acknowledged. Additionally, the Publisher has updated a few typographical errors in the article title, abstract, and Acknowledgements section, which were introduced after the article proof had been reviewed by the corresponding author.

