# Supplementary material for: Domain architecture of plant eukaryotic translation initiation factor 3 subunit E governs interaction with translational cis-elements to regulate pollen tube growth
Source: Plant Cell. 2026 Feb 17;38(2):koag005. doi: 10.1093/plcell/koag005 (PMC13043079; doi:10.1093/plcell/koag005)
Supplement: koag005_Supplementary_Data [file koag005_supplementary_data.zip › Supplementary Video S1 2025.pptx]

## Slide 1
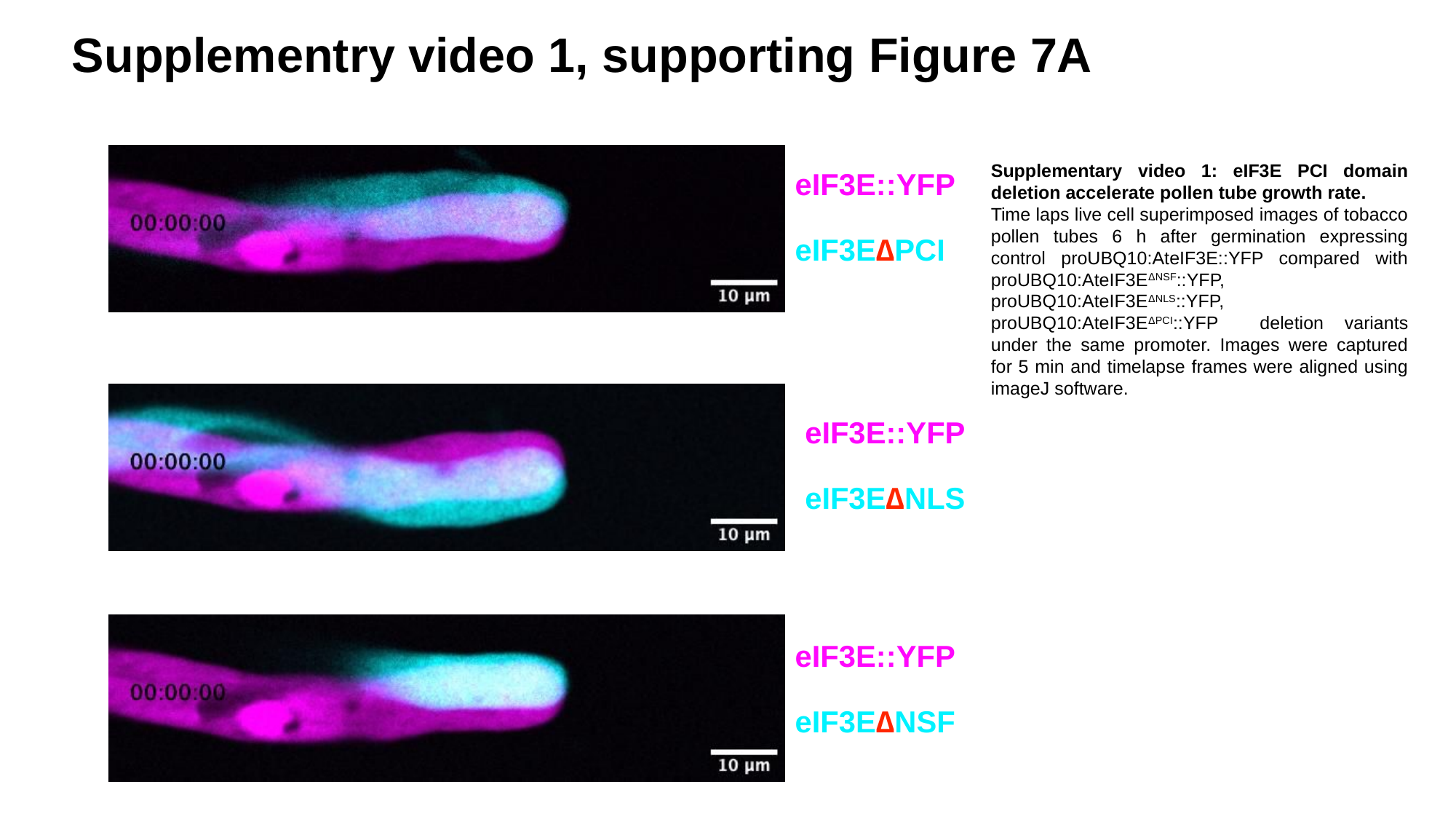

Supplementry video 1, supporting Figure 7A
Supplementary video 1: eIF3E PCI domain deletion accelerate pollen tube growth rate.
Time laps live cell superimposed images of tobacco pollen tubes 6 h after germination expressing control proUBQ10:AteIF3E::YFP compared with proUBQ10:AteIF3EΔNSF::YFP, proUBQ10:AteIF3EΔNLS::YFP, proUBQ10:AteIF3EΔPCI::YFP deletion variants under the same promoter. Images were captured for 5 min and timelapse frames were aligned using imageJ software.
eIF3E::YFP
eIF3E∆PCI
eIF3E::YFP
eIF3E∆NLS
eIF3E::YFP
eIF3E∆NSF
