# Supplementary material for: Domain architecture of plant eukaryotic translation initiation factor 3 subunit E governs interaction with translational cis-elements to regulate pollen tube growth
Source: Plant Cell. 2026 Feb 17;38(2):koag005. doi: 10.1093/plcell/koag005 (PMC13043079; doi:10.1093/plcell/koag005)
Supplement: koag005_Supplementary_Data [file koag005_supplementary_data.zip › Supplementary Video S2 2025.pptx]

## Slide 1
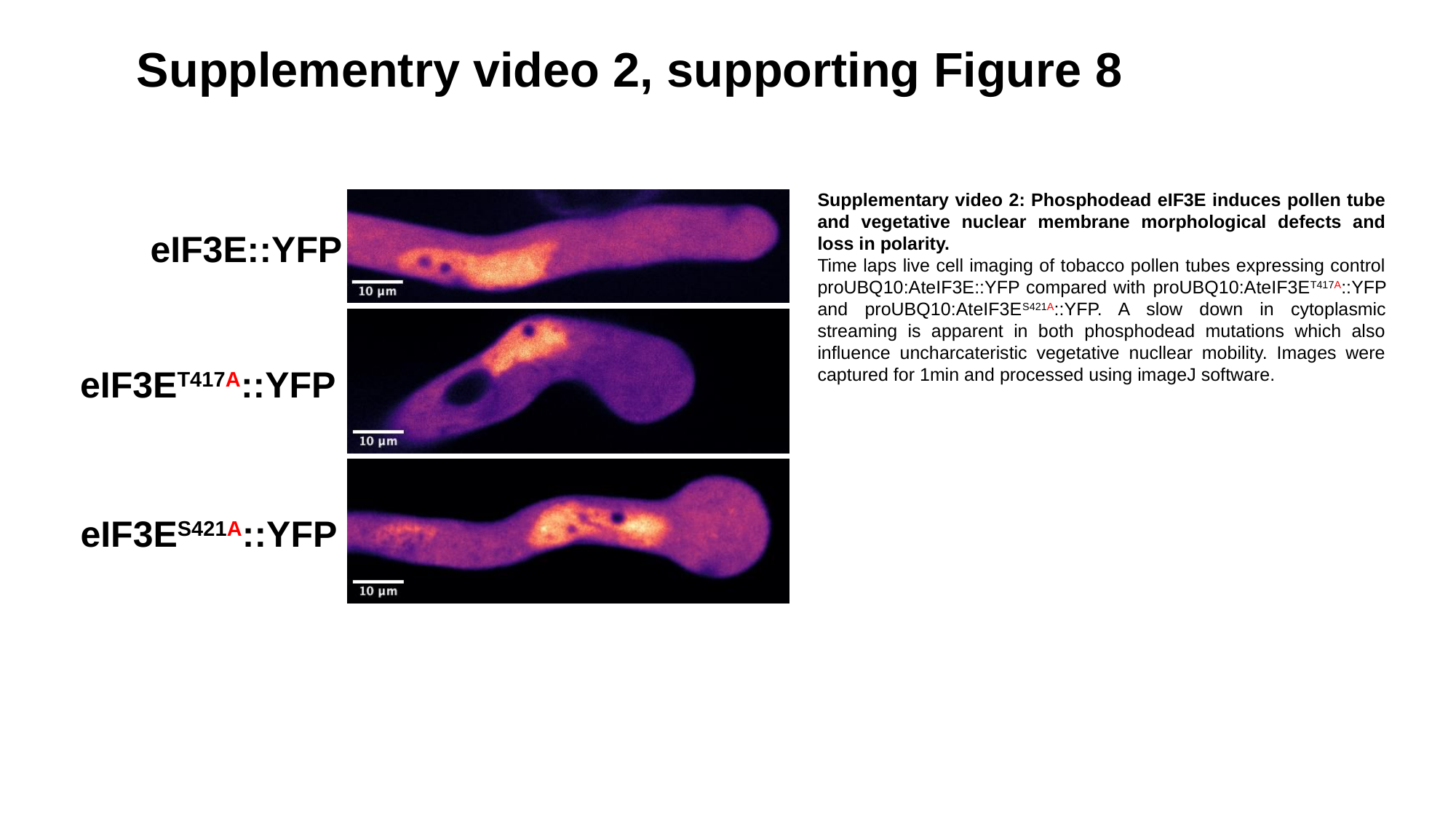

Supplementry video 2, supporting Figure 8
Supplementary video 2: Phosphodead eIF3E induces pollen tube and vegetative nuclear membrane morphological defects and loss in polarity.
Time laps live cell imaging of tobacco pollen tubes expressing control proUBQ10:AteIF3E::YFP compared with proUBQ10:AteIF3ET417A::YFP and proUBQ10:AteIF3ES421A::YFP. A slow down in cytoplasmic streaming is apparent in both phosphodead mutations which also influence uncharcateristic vegetative nucllear mobility. Images were captured for 1min and processed using imageJ software.
eIF3E::YFP
eIF3ET417A::YFP
eIF3ES421A::YFP
