# Supplementary material for: Domain architecture of plant eukaryotic translation initiation factor 3 subunit E governs interaction with translational cis-elements to regulate pollen tube growth
Source: Plant Cell. 2026 Feb 17;38(2):koag005. doi: 10.1093/plcell/koag005 (PMC13043079; doi:10.1093/plcell/koag005)
Supplement: koag005_Supplementary_Data [file koag005_supplementary_data.zip › TPC-2025-0418R2_Supplementary Video Legends.docx]

**Supplementary Video 1:** Time lapse video comparing the growth rate of *in vitro* grown tobacco pollen tubes expressing WT AteIF3E:YFP with eIF3E deletion variants AteIF3E**^Δ^**^NSF^, AteIF3E^ΔNLS^ and AteIF3E^ΔPCI^.

**Supplementary Video 2:** Time lapse video of induced defective vegetative cell nuclear membrane morphology in pollen tubes expressing AteIF3E^T417A^::YFP or AteIF3E^S421A^::YFP phosphodead eIF3E variants. The phenotype was observed 6 h after tobacco pollen transformation.
