# Supplementary material for: Domain architecture of plant eukaryotic translation initiation factor 3 subunit E governs interaction with translational cis-elements to regulate pollen tube growth
Source: Plant Cell. 2026 Feb 17;38(2):koag005. doi: 10.1093/plcell/koag005 (PMC13043079; doi:10.1093/plcell/koag005)
Supplement: koag005_Supplementary_Data [file koag005_supplementary_data.zip › Supplementary Data_revised.pdf]

Supplementary Data

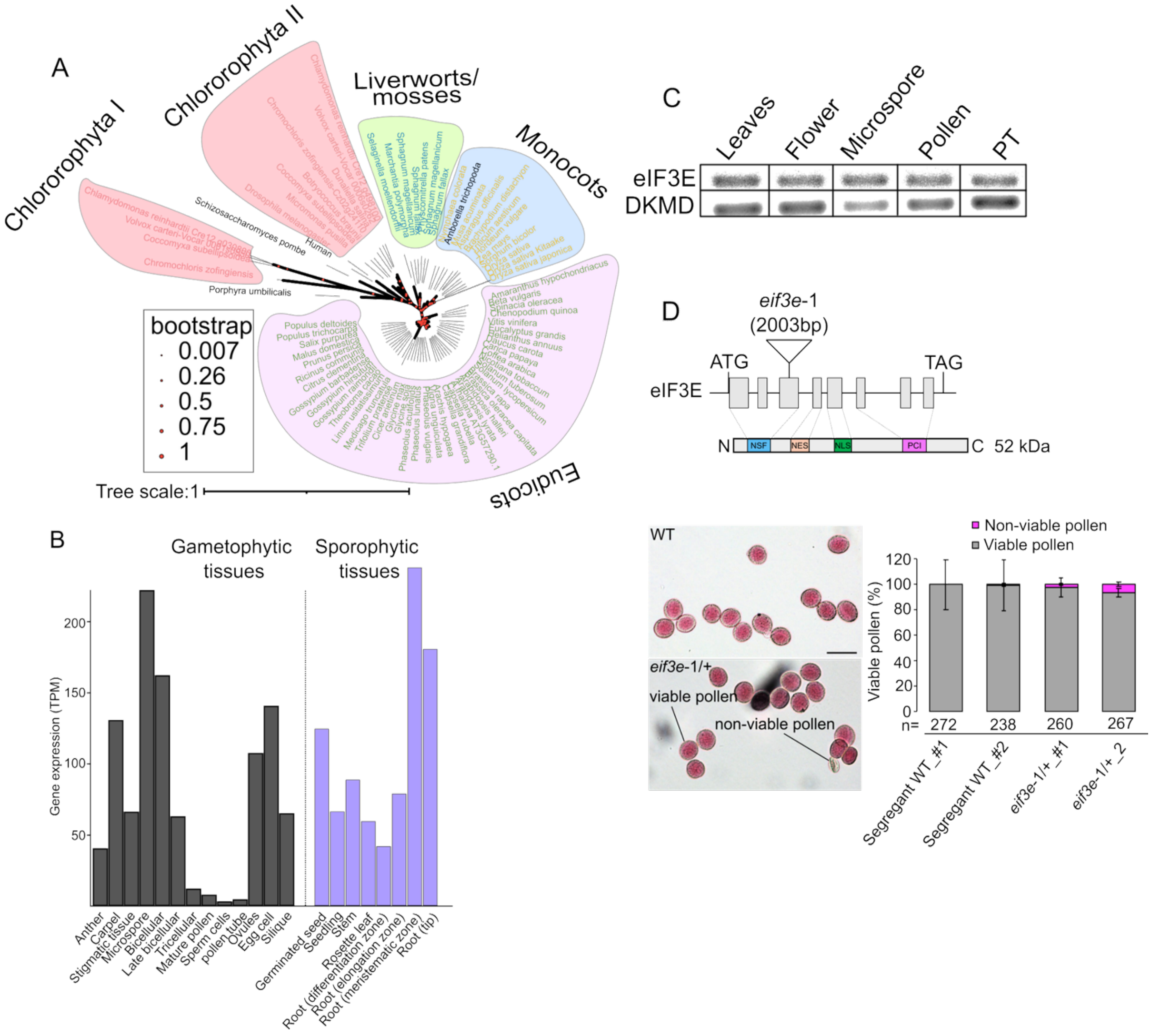

Supplementary Figure S1. eIF3E structural and functional conservation.

(**A**) The eIF3E protein and its ortholog sequences were downloaded from phytozome v13 and NCBI databases. Phylogenetic tree was constructed using the maximum likelihood method and the scale bar represents the rate of substitution of amino acids. (**B, C**) Public extracted RNAseq data (CoNeKT, Proost S and Mutwil M., 2019) and semi-RT PCR results further validated NteIF3E expression in both sporophytic and reproductive tissues such as leaves, flower, microspore, pollen and PT (pollen tube). TPM (Transcripts Per Million). DKMD housekeeping gene was used as a control (Primers are listed in Supplementary Table S3). This figure support data from Figure 1 on phylogenetic distribution and domains structure of eIF3E. **(D)** Gene model showing the T-DNA *eif3e*-1 mutant allele position at the 3rd exon of the eIF3E used in this study for functional assays. Below, Alexander staining revealed that both wild

type and *eif3e*-1/+ mature pollen had viable mature pollen cytoplasm. Error bars indicate ±SD, n= pollen population size. scale bar= 10 μm.

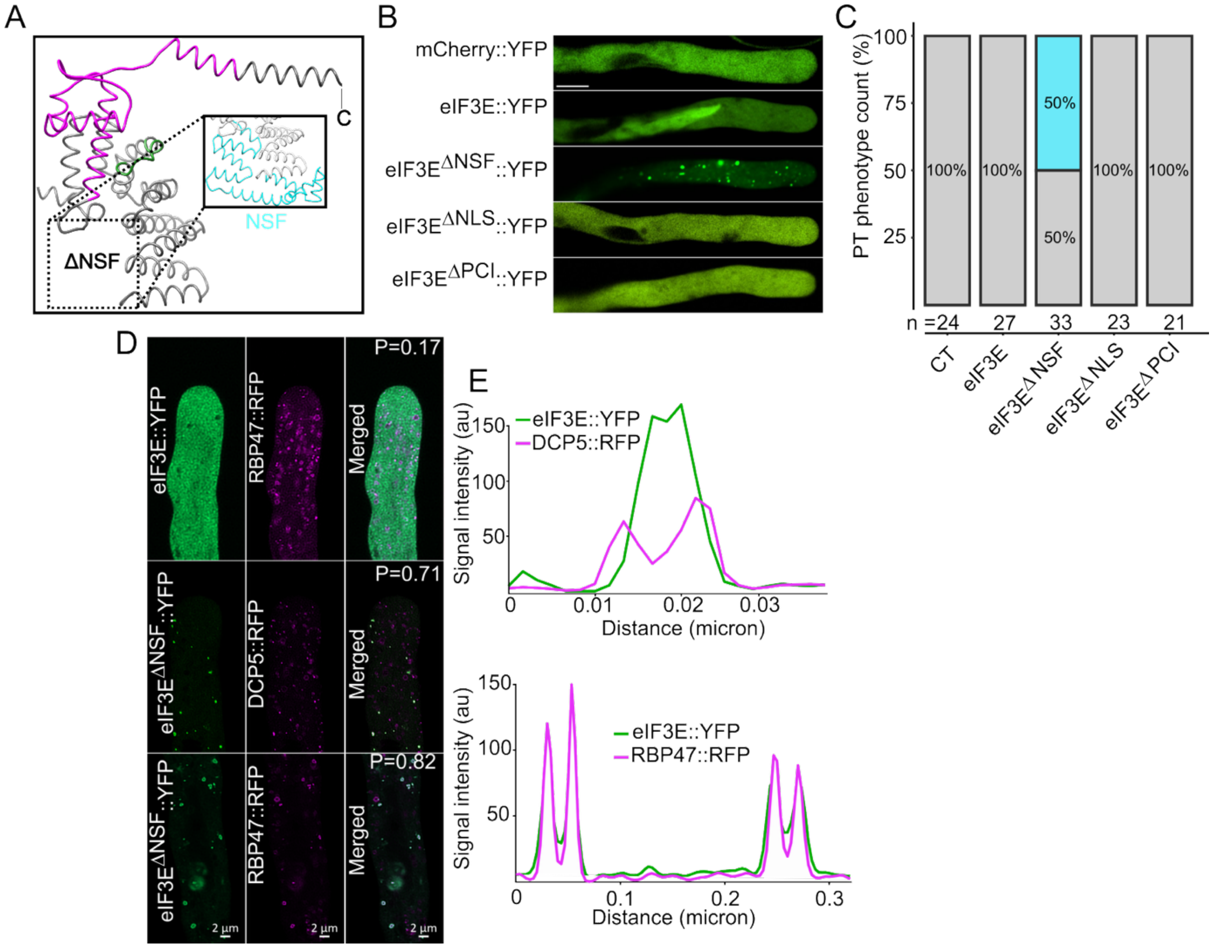

Supplementary Figure S2. Truncated NteIF3E$^{\Delta NSF}$ induces cytoplasmic aggregates in tobacco pollen tubes. (A) Predicted 2D secondary structure of the NteIF3E deletion variant NteIF3E$^{\Delta NSF}$, highlighting the N-terminal region. The dotted rectangle indicates the omitted N-superfamily domain. **(B)** Confocal images of transient expression of control mCherry:YFP, full length NteIF3E::YFP, NteIF3E$^{\Delta NSF}$, NteIF3E$^{\Delta NLS}$ and NteIF3E$^{\Delta PCI}$ 10 h post transformation. Scale bar = 10 μm applied to all panel images **(C)** Quantification of frequency of pollen tubes displaying the cytoplasmic aggregates 10 h after pollen transient transformation. **(D, E)** NteIF3E$^{\Delta NSF}$ induced cytoplasmic aggregates strongly co-localizes with stress granule marker RBP47::RFP and mRNA storage processing body marker DCP5::RFP in pollen tubes. Scale bar = 2 μm for all panel. For co-localization, images were analysed by ImageJ using JACoP option in plugin and plot profile. This figure support data from Figure 2 on the effect of NteIF3E domains deletion and formation of protein aggregates.

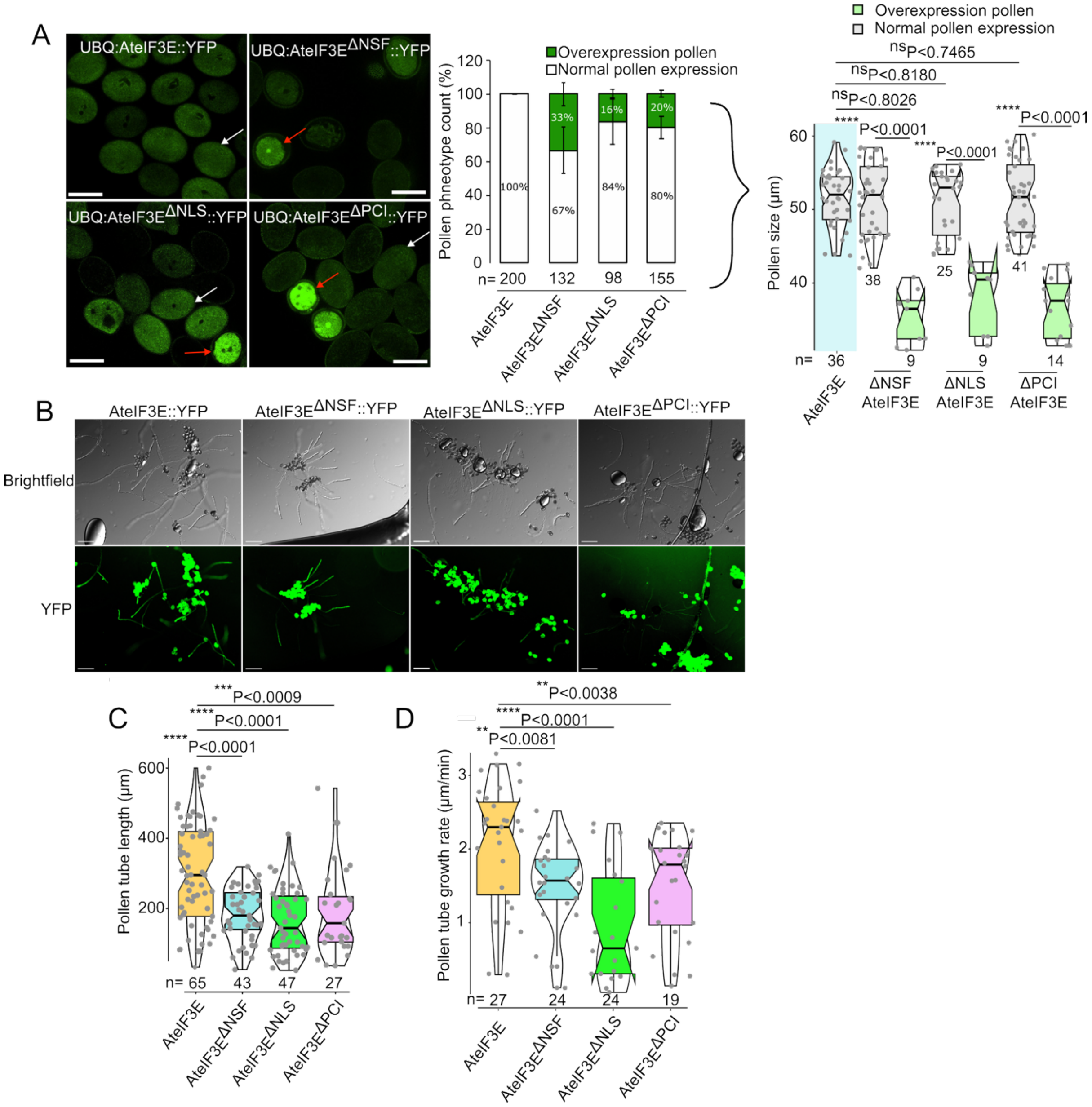

Supplementary Figure S3. The N-Super Family, bipartite NLS and the PCI domains are critical for eIF3E function in Arabidopsis. (**A**) Unlike the wild-type *pUBQ10:AteIF3E::YFP* expression in the Col-0 background, overexpression of all *AteIF3E* domain-deletion variants caused excessive protein accumulation and produced abnormally small pollen with fragmented cytoplasm in a dominant-negative manner. Right: Frequency of the small-pollen phenotype for each deletion variant and pollen size distribution compared with normal fluorescent pollen. Error bars indicate ±SD. Far right: All deletion variants showed two distinct pollen populations: one with normal-sized grains exhibiting YFP expression comparable to full-length *AteIF3E*, and another with smaller grains displaying elevated YFP expression relative to the full-length protein. (**B**) *In vitro* pollen tube germination performed using stable

transgenic lines expressing AteIF3E::YFP, AteIF3E$^{\Delta NSF}$::YFP, AteIF3E$^{\Delta PCI}$::YFP and AteIF3E$^{\Delta NLS}$::YFP. Images were captured in Zeiss AxioImager ApoTome2 after 4 h post pollen germination. (**C, D**) Pollen tube length and growth rate of full-length *AteIF3E* were compared with its deletion variants by measuring segmented lines from the base of tube emergence to the tip. Notched box plots were used to assess the statistical reduction in pollen tube length in the Arabidopsis Col-0 background. Embedded notch-box plot in violin plot representing pollen phenotype (figure S3A) and pollen tube growth rate and length (figure S3C and S3D), where center line represent the median and the first and third quartiles indicate 25th and 75th percentiles, and the whiskers extend from minimum to maximum with 1.5 times the interquartile range from the 25th and 75th percentiles, whereas all the data points are shown as grey dots. Statistical analyses in panels S3A, C, and D were performed using an unpaired nonparametric Mann–Whitney U test in GraphPad Prism 9.1.1. This figure supports the data from Figures 2 and 7, highlighting the conserved function of Arabidopsis AteIF3E and tobacco NteIF3E in regulating pollen tube growth rate and overall tube length, and demonstrates the reproducible dominant-negative effects of eIF3E domain deletions in stable Arabidopsis pollen tubes. n= pollen population size. Scale bars= 10 µm applied to figure panel A and B.

A

AlphaFold3					
**Bait-prey combination**	**ipTM**	**pTM**	**Model confidence**	**Residue or atom pairs within distance 3Å**	**Interaction region reference to eIF3E**
eIF3E-eIF3L	0.48	0.55	0.494	13	PCI domain
eIF3E$^{\Delta PCI}$-eIF3L	0.2	0.54	0.268	11	N-terminal part
eIF3E-eIF3D	0.68	0.48	0.644	19	PCI domain
eIF3E-eIF3C	0.7	0.61	0.682	12	C-terminal part, PCI domain
eIF3E-eIF3A	0.17	0.4	0.216	6	PCI domain
eIF3E-eIF3G	0.26	0.42	0.292	2	PCI domain

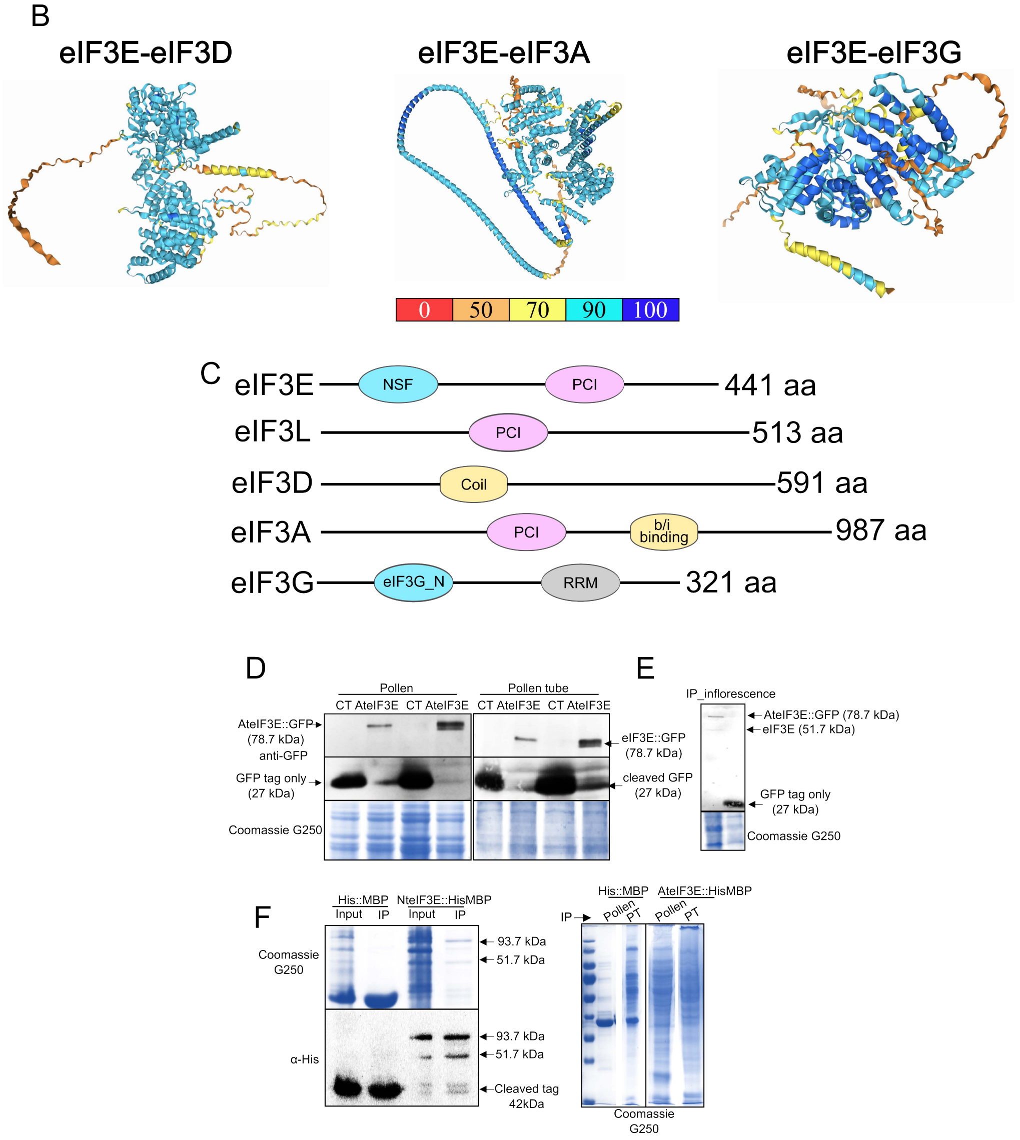

Supplementary Figure S4. eIF3E Alphafold 3 and ColabFold AlphaFold 2-RoseTTAFold 2D protein-protein interaction predictions.

(A-B) AlphaFold3 predictions, based on ipTM and pTM scores, indicate that eIF3E interacts with eIF3D, eIF3L, and eIF3C via its C-terminal PCI domain, but no significant interaction was predicted with eIF3A or eIF3G. **(B)** Interaction structure of AteIF3E with AteIF3D, AteIF3A and AteIF3G. Comparative structures of Arabidopsis-related protein-protein interactions as predicted by AlphaFold2/3 models is shown. The structure is color-coded based on pLDDT score representing per-residue confidence scores. Dark blue signifies a pLDDT score> 90, indicating very high confidence, light blue indicates pLDDT score between 70 to 90, imply confidence, yellow represent a pLDDT score in between 50 to 70, defining low confidence and orange suggesting a pLDDT score < 50, indicating a very low confidence on those residues. Contacts point per atom and domains are highlighted in UCSF ChimeraX. **(C)** Domain structure of eIF3 complex subunits predicted by PROSITE and SMART using InterPro. (**D**) Immunoblot of the control CT (proAteIF3E:YFP tag only) and native proAteIF3E:eIF3E::YFP representative samples isolated from dry pollen, 4 h *in vitro* pollen tubes and (**E**) open flowers, detected with anti-GFP antibody (Agrisera). (**F**) LC-MS/MS peptide analysis of co-IP samples shows enrichment of eIF3 subunits in native promoter AteIF3E::YFP samples (native), but not in the YFP-only control (CT).

(**G**) Raw peptide counts from LC-MS/MS of an *in vitro* pull-down assay, where recombinant NteIF3E::His:MBP (3E rec) was incubated with protein extracts from mature pollen and pollen tubes.
CT, HIS::MBP tag only control. (**H**) Validation of the *in vitro* interaction. (Left) Immunoblot with anti-His antibody confirming the capture of NteIF3E::His:MBP and the control His::MBP. (Right) Coomassie-stained gel of input and IP samples from the LC-MS/MS shown in (G). This figure supports the data from Figure 3 on eIF3E interactome and pairwise interaction.

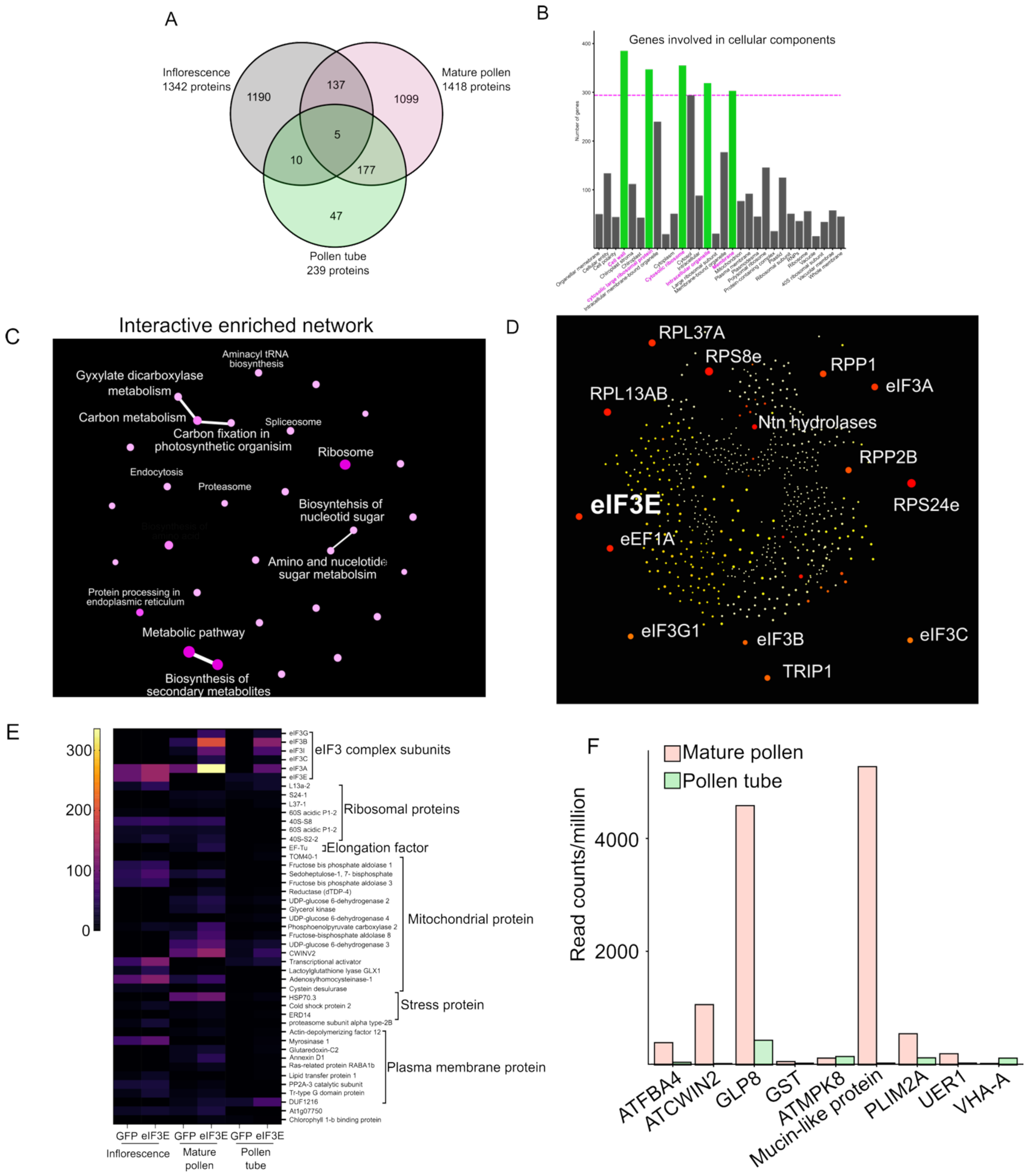

Supplementary Figure S5. AteIF3E interactome in Arabidopsis reproductive tissues. (**A**) Venn diagram of the total AteIF3E::YFP interactome. (**B**) Gene ontology (GO) enrichment analysis of the interactome, categorized by biological function. (**C**) Pathway interaction network. Nodes represent enriched pathways, sized by gene set size and darkened by significance. Edges connect pathways sharing ≥20% of their genes, with thickness scaled to the degree of overlap. (**D**) Co-expression network analysis generated by Function Explorer. Node color

indicates expression pattern, from up-regulated (red) to neutral (yellow). (**E**) Heatmap of protein abundance (log2-fold change relative to GFP-tag control), based on LC-MS/MS peptide counts. Color intensity scale bar represents relative protein abundance found in each sample with low value shown in dark purple (lowest detected protein levels to yellow (highest detected protein levels). (**F**) Relative transcript abundance of identified membrane proteins, based on public RNA-seq data (Klodova et al., 2023). This figure extends the data from Figure 3, providing a comprehensive analysis of the Arabidopsis eIF3E interactome.

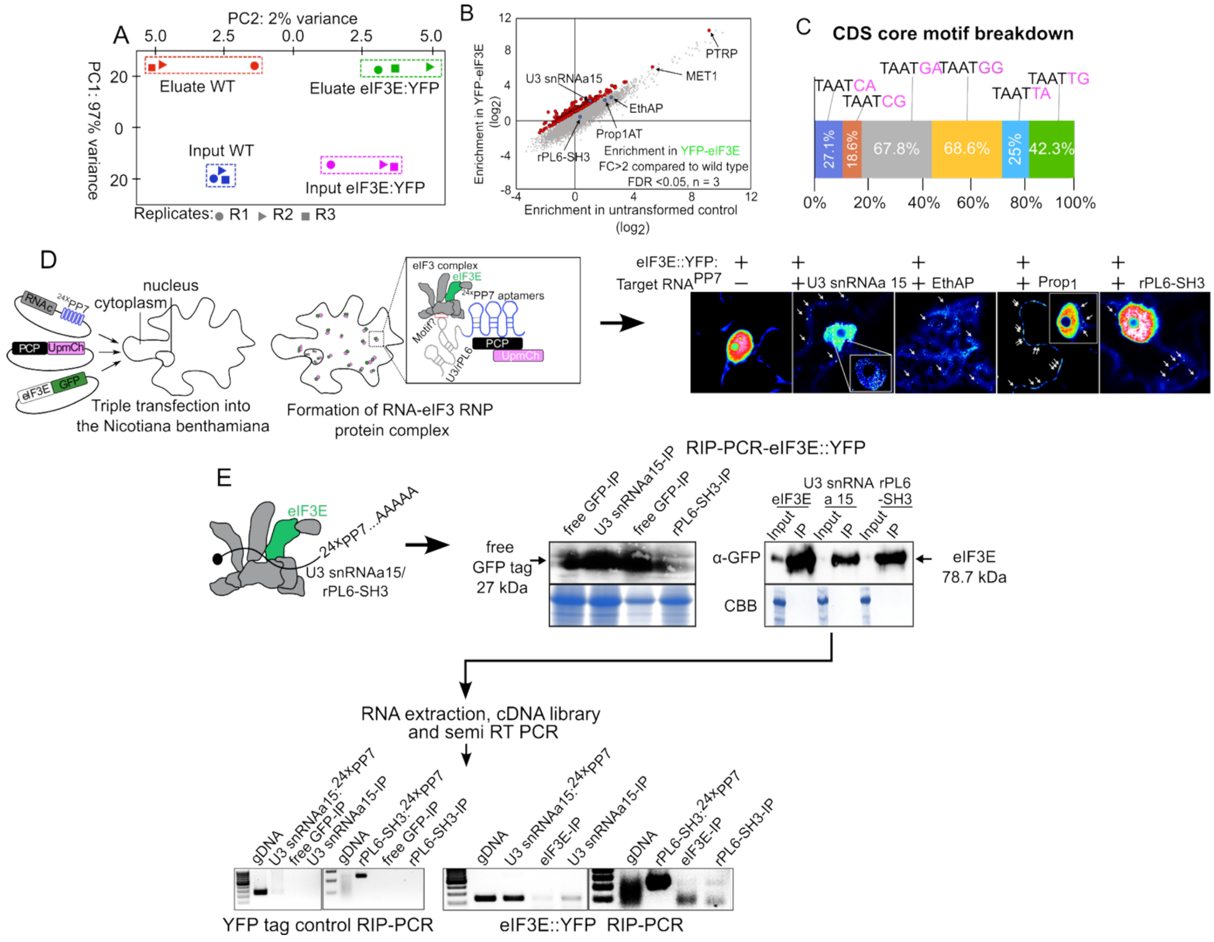

Supplementary Figure S6. PP7-aptamer RNA tagging confirm eIF3E-RNA target association *in vivo*. (**A**) PCA analysis of the eIF3E-RIPseq data showing replicates grouping reflecting biological and technical reproducibility. (B) Scatter plot of eIF3E-enriched transcripts from tobacco pollen tubes. Red dots represent the normalized log2 read counts of 191 AteIF3E co-immunoprecipitated targets significantly enriched in the AteIF3E::YFP RIP-seq library compared with total co-immunoprecipitated RNA (FDR < 0.05, n = 3). The complete list is provided in Supplementary Table S2, and

raw data are available at NCBI (accession PRJNA1049184). (**C**) Dominant patterns of the top CDS MC1 *cis*-element account for >90% (171 genes) of the eIF3E mRNA targets. (**D**) *In vivo* labeling of AteIF3E target RNAs using bacterial PP7 aptamer repeats, and visualization of pUBQ10:eIF3E-target RNP formation in transiently transformed tobacco leaf pavement cells. The construct pUBQ10:PCP::mCherry, encoding the bacterial PP7 coat protein fused to mCherry, was used as an additional reporter to recognize and bind the PP7 aptamer repeats. Arrows represent RNP formation labelled following eIF3E::YFP binding to the target mRNA (**E**) RIP-PCR (semi-qRT-PCR) validation of eIF3E-associated target RNAs. Candidate RNAs with medium-to-low confidence scores from RIP-seq were selected to assess the sensitivity of the approach and to test the verification of low-confidence targets. Selected RNAs, *U3 snRNA* and *SH3-rPL6* mRNA, were co-transiently expressed either with proUBQ10:AteIF3E::YFP, with the free proUBQ10:YFP tag, or alone in wild-type plants. Pull-down assays were performed using anti-GFP agarose beads (ChromoTek). Left: Immunoblot showing detection of eIF3E::YFP and free YFP with anti-GFP antibody, confirming successful pull-down. Below: Semi-RT PCR from cDNA libraries prepared from bead-precipitated RNAs demonstrates the association of eIF3E with its target RNAs. Non-specific binding was evaluated by the free YFP and wild-type samples. This figure supports data from Figure 5 on eIF3E-associated mRNA targets and their validation by RNP co-localization and RIP-PCR.

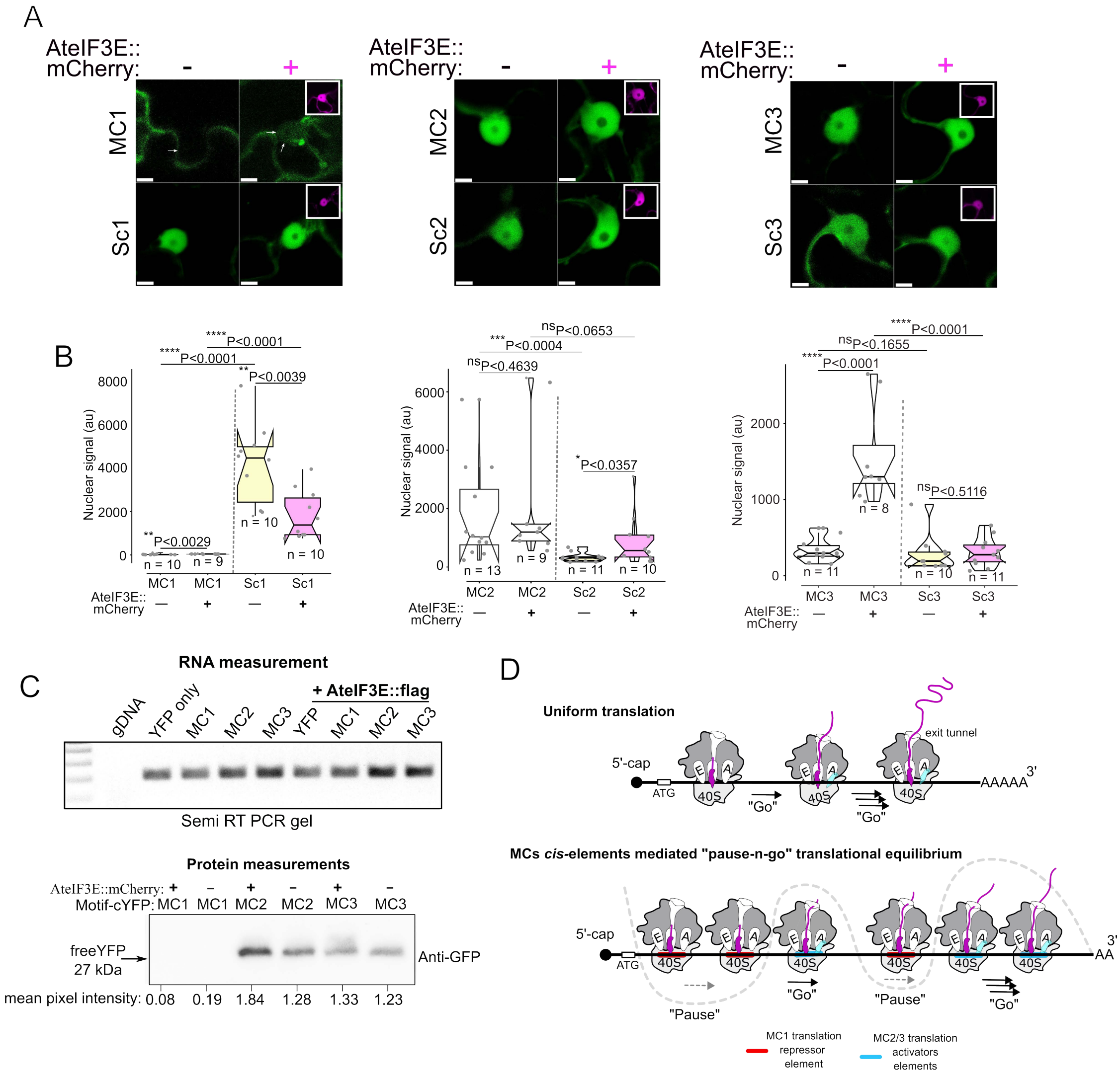

Supplementary Figure S7. eIF3E-dependent MC1-MC3 translation repression-activation function similarly in sporophyte. (**A, B**) Transient expression of the eIF3E co-immunoprecipitated enriched CDS motifs (MC) RNA reporters, MC1::YFP, MC2::YFP and MC2::YFP and their respective scramble motifs, Sc1, Sc2 and Sc2 in *Nicotiana benthamiana* leaves. These results confirm the conserved gametophytic activity of MC1 and MC2 cis-elements in regulating YFP RNA reporter translation within sporophytic tissues. Specifically, the MC1 motif functions as a translational repressor, while the MC2/MC3 motifs act as translational activators, in an AteIF3E-dependent manner, consistent with observations in transient tobacco pollen tubes and in Arabidopsis Col-0 and *eif3e-1/+* stable transgenic lines (see Figure 5). Embedded notch-box plot in violin plot, where center line represent the median and the first and third quartiles indicate 25th and 75th percentiles, and the whiskers extend

from minimum to maximum with 1.5 times the interquartile range from the 25th and 75th percentiles, whereas all the data points are shown as grey dots representing YFP signal intensity. Statistical significance was assessed using the Mann–Whitney U test (unpaired, nonparametric) in GraphPad Prism 9.1.1. Scale bars= 10 µm applied to all the images to figure 1A panels (**C**) Semi-qRT-PCR and western blot analyses of YFP transcripts and protein expression were performed with at least three independent replicates. All motifs showed no variation at the transcriptional level of the YFP mRNA reporter but revealed strong effects at the translational level. (**D**) Summary schematic illustrating the proposed effect of tandem MC1–MC2–MC3 *cis*-elements on target RNA translation. eIF3E maintains translational balance by inducing ribosome slowdown (pausing) at MC1 repressor *cis*-elements, while promoting reactivation and stability through MC2/MC3 *cis*-elements, thereby ensuring optimal protein abundance. This model supports the data presented in Figure 5 on eIF3E-dependent translational repression–activation mediated by MC1–MC2/3 cis-elements.

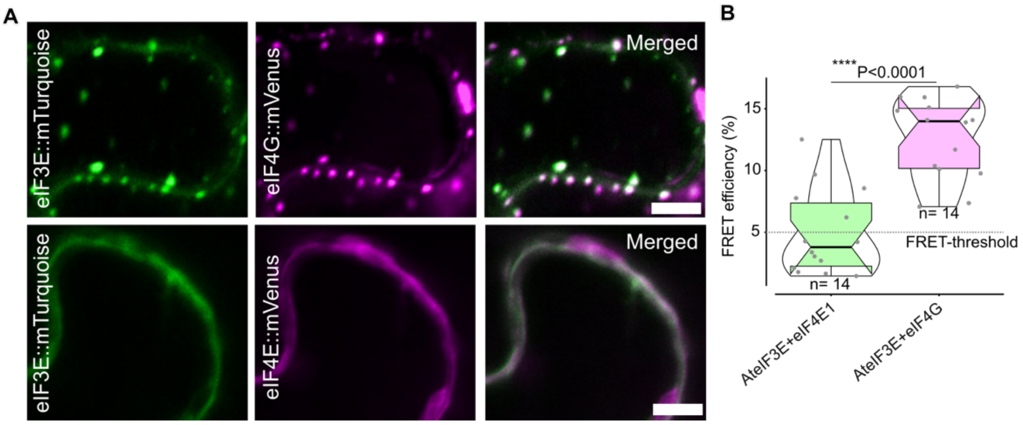

Supplementary Figure S8. eIF3E could associate with its mRNA targets via interaction with eIF4G subunit of the eIF4F 5'-cap complex.

(**A**) Transient co-expression of AteIF3E::mTorquoise with eIF4G::mVenus or eIF4E::mVenus in Nicotiana benthamiana leaf under the UBQ10 promoter. AteIF3E strongly co-localize with eIF4G in RNP granules and likely with eIF4E in the cytosol. Scale bars = 5 µm . (**B**) preliminary FRET data demonstrated direct interaction between AteIF3E with AteIF4G but not with AteIF4E. Notch-box plot in violin plot, the center line represent the median and the first and third quartiles indicate 25th and 75th percentiles, and the whiskers extend from minimum to maximum with 1.5 times the interquartile range from the 25th and 75th percentiles, whereas all the data points are shown as grey dots representing FRET efficiency. In human, the eIF3 complex,

which includes eIF3E (INT6), bridges the 43S pre-initiation complex to the mRNA 5′ cap via the eIF4F complex (eIF4E, eIF4G, and eIF4A). eIF3E contributes to this bridge through interactions (direct or within the eIF3 subcomplex) with eIF4G, facilitating mRNA recruitment to the ribosome (LeFebvre et al., 2006; Masutani et al., 2007). This study provides preliminary evidence for a direct interaction between At-eIF3E and At-eIF4G; further experiments are necessary to confirm whether this interaction acts as a bridge for eIF3E-mRNA association. This figure support the working model presented in Figure 9. Scale bar = 5 µm for all panels.

Supplementary Table S3. List of primers used

	Primer name	Primer sequences (5'-3')
1.	attB1F	GGGGACAAGTTTGTACAAAAAAGCAGGCTTA
2.	attB2R	GGGGACCACTTTGTACAAGAAAGCTGGGTG
3.	attB3F	GGGGACAACTTTGTATAATAAAGTTGCAATG
4.	attB4R	GGGGACAACTTTGTATAGAAAAGTTGGGTG
5.	attB4R	GGGGACTGCTTTTTTGTACAAACTTG
6.	attB1R	GGGGACAACTTTGTATAGAAAAGTTG
7.	pUBQ10-attB4F	TGTATAGAAAAGTTGCTCAACAACAAACTTTCCATT
8.	pUBQ10-attB1R	TTTTGTACAAACTTGCTGTTAATCAGAAAAACTCAG
9.	eIF3E-CDS-attB2F	TTCTTGTACAAAGTGGCTATGGAGGAAAGCAAACAGAACTA
10.	eIF3E-CDS+STOP-attB3R	TGTATAATAAAGTTGCTAGCGAGTTGCTTGCGCCTGTG
11.	eIF3E-CDS-attB1F	AAAAAAGCAGGCTTAATGGAGGAAAGCAAACA
12.	eIF3E-CDS+STOP-attB2	GTACAAGAAAGCTGGGTGCTAGCGAGTTGCTTGC
13.	M13F	GTAAAACGACGGCCAGT
14.	M13R	CAGGAAACAGCTATGAC
15.	GFP-attB1F	AAAAAAGCAGGCTTAATGGTGAGCAAGGGCGAGGAGCT
16.	GFP-attB2R	AAGAAAGCTGGGTGCTTGTACAGCTCGTCCATGCCGA
17.	SALK-121004-LP	AACCTGGACAGACACTTGGTG
18.	SALK-121004-RP	AACGCAAGGAAATAGATGGTG
19.	LB1.3	ATTTTGCCGATTTCGGAAC
20.	*eif3e*-1-qRTF2	GGAAGGACACAGATCATTGA
21.	*eif3e*-1-qRTR2	TTGGATCTTTGTAGGAGTAG
22.	eIF3E-deltaN.SE-F	AGAACTATGCTGCTGATTATCTTTACCAGTAC
23.	eIF3E-deltaN.SE-R	CAGCAGCATAGTTCTGTTTGCTTTCCTCC
24.	eIF3E-deltaNLS-F	CTGCTTTCAAAGAATTCATTAAGGTCATTCAGC
25.	eIF3E-deltaNLS-R	ATTCTTTGAAAGCAGTTGCCAAGTAGC
26.	eIF3E-deltaPCI-F	CAACTGTACGAACATACAAGTTAGTGAATCAGC
27.	eIF3E-deltaPCI-R	ATGTTCGTACAGTTGAAAAGTTTCCATCCTC
28.	KAKU4-CDS-attB1F	AAAAGCAGGCTTAATGGATTCCGTCTCCGG
29.	KAKU4-CDS-attB2R	AGAAAGCTGGGTGTTTGGCCCGTCCTTTGC
30.	Nt-eIF3E-CDS-attB1F	ACAAAAAAGCAGGCTTAATGGCGGCGAAGTACGACC
31.	Nt-eIF3E-CDS-attB2R	TACAAGAAAGCTGGGTGGCGAGCAGCCTGTGTTTG
32.	NtDKMD-RT-PCR-FP	CGTGAGCCAAAGGAATTTGT
33.	NtDKMD-RT-PCR-RP	GAGCCTCCCGAATTTTCTTC
34.	eIF3E-Fw-Threonine (417)-CDS-Alanine	CAGTTGATAAACCACGCCAAAGGCTTATCAGGA
35.	eIF3E-R-Threonine (417)-CDS-Alanine	TCCTGATAAGCCTTTGGCGTGGTTTATCAACTG
36.	eIF3E-Fw-Serine (421)-CDS- Alanine	CACACCAAAGGCTTAGCAGGACGAACATACAAG
37.	eIF3E-R-Serine (421)-CDS-Alanine	CTTGTATGTTCGTCCTGCTAAGCCTTTGGTGTG
38.	eIF3L-CDS-attB1F	AAAAAAGCAGGCTTAATGGCGAGCAGCAATG
39.	eIF3L-CDS-attB4R	AGAAAAGTTGGGTGTTCCAGCTTGACACG
40.	eIF3L-CDS+STOP-attB2R	ACAAGAAAGCTGGGTGTCATTCCAGCTTGACACGATC
41.	eIF3D1-CDS-attB1F	TACAAAAAAGCAGGCTATGGTAACCGAAGCTTTCG
42.	eIF3D1-CDS-attB4R	ATAGAAAAGTTGGGTGAGCTTGAGCATCATCAGCTT
43.	eIF3D1-CDS+STOP-attB2R	ACAAGAAAGCTGGGTGAGCTTGAGCATCATCAGCTT
44.	eIF3G1-CDS-attB1F	CAAAAAAGCAGGCTTAATGACGATCGATTCGC
45.	eIF3G1-CDS-attB4R	TAGAAAAGTTGGGTGGGTTGGTCTTGGAGTTG
46.	eIF3A-CDS-attB1F	CAAAAAAGCAGGCTTAATGGCGAATTTTGCCAAA
47.	eIF3A-CDS-attB4R	ATAGAAAAGTTGGGTGACGCTGTGTTGGCCTGGG
48.	eIF3E-deltaN.SE-CDS-attB3F	AATAAAGTTGCAATGGAGGAAAGCAAACAG
49.	eIF3E-deltaN.SE-CDS-attB2R	AAGAAAGCTGGGTGGCGAGTTGCTTGCGCCTGT
50.	eIF3E-deltaPCI-CDS-attB3F	AATAAAGTTGCAATGGAGGAAAGCAAACAG
51.	eIF3E-deltaPCI-CDS-attB2R	AAGAAAGCTGGGTGGCGAGTTGCTTGCGCCTGT
52.	EthAP-CDS-attB1F	GGGGACAAGTTTGTACAAAAAAGCAGGCTCTATGAGGACCGATGGGATCTTA
53.	EthAP-CDS-attB2R	GGGGACCACTTTGTACAAGAAAGCTGGGTCCATTTTTTTACGAGAAAACTGC
54.	rPL6-SH3-CDS-attB1F	GGGGACAAGTTTGTACAAAAAAGCAGGCTCTATGGCGCCCAAGAAAACAATC
55.	rPL6-SH3-CDS-attB2R	GGGGACCACTTTGTACAAGAAAGCTGGGTCAAAGACTAACTCATGTGGTCTC
56.	Prop1AT-CDS-attB1F	GGGGACAAGTTTGTACAAAAAAGCAGGCTCTATGACAAGGTCCCGGGACCG
57.	Prop1AT-CDS-attB2R	GGGGACCACTTTGTACAAGAAAGCTGGGTCACTCAGAGACCTACGCATCTC
58.	U3 snRNAa15-CDS-attB1F	GGGGACAAGTTTGTACAAAAAAGCAGGCTCTATGGCAGAAAATCAATCCATAG
59.	U3 snRNAa15-CDS-attB2R	GGGGACCACTTTGTACAAGAAAGCTGGGTCTCTCCTTGCAGCAATCTTG
60.	U3 snRNAa15 RT-PCR F2	GGTAGCTGCTTTGAGTGGG
61.	U3 snRNAa15 RT-PCR R2	GTGACCTCTCAGTTCATCTG
62.	rPL6-SH3 RT-PCR F2	GTTGAGAAGACTGATGATAAGTAT
63.	rPL6-SH3 RT-PCR R2	GACTAACTCATGTGGTCTCATGC

64.	eIF3E-x4-Motif-1-F'P	CACCGTTAATGGTAATGGTAATGGTAATGG
65.	eIF3E-x4-Motif-1-R'P	CCATTACCATTACCATTACCATTAAC
66.	eIF3E-x4-Scramble-1-F'P	CACCTGATTTTGAAAGATTATGGTGAGGGA
67.	eIF3E-x4-Scramble-1-R'P	TCCCTCACCATAATCTTTCAAAATCA
68.	eIF3E-x3-Motif-2-F'P	CACCAAAGTAAAAAGTAAAAAGTAA
69.	eIF3E-x3-Motif-2-R'P	TTACTTTTTACTTTTTACTTT
70.	eIF3E-x3-Scramble-2-F'P	CACCAGAAAAAAAAATTAAGTAAGA
71.	eIF3E-x3-Scramble-2-R'P	TCTTACTTAATTTTTTTTTCT
72.	eIF3E-x3-Motif-3-F'P	CACCCTTTTTCTTTTTCTTTTT
73.	eIF3E-x3-Motif-3-R'P	AAAAAGAAAAAGAAAAAG
74.	eIF3E-x3-Scramble-3-F'P	CACCTCTTTTTTTCTTTTTTTC
75.	eIF3E-x3-Scramble-3-R'P	GAAAAAAAGAAAAAAAGA
76.	sYFP-SeqF	ATGGTGAGCAAGGGCGA
77.	sYFP-SeqR	AGGTGGTCACGAGGGTA
