# Supplementary figures and images for: Domain architecture of plant eukaryotic translation initiation factor 3 subunit E governs interaction with translational cis-elements to regulate pollen tube growth
Source: Plant Cell. 2026 Feb 17;38(2):koag005. doi: 10.1093/plcell/koag005 (PMC13043079; doi:10.1093/plcell/koag005)

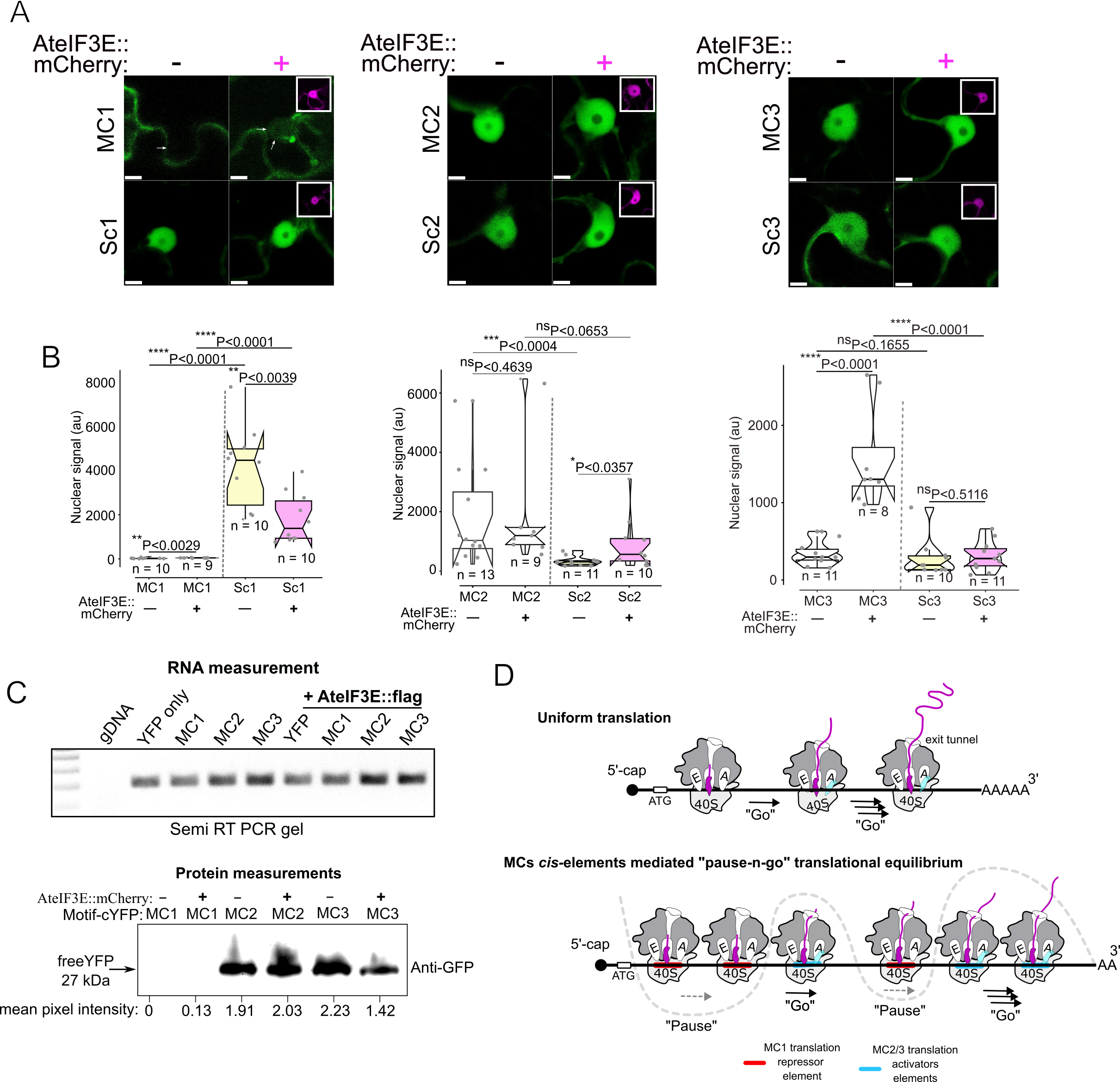

Supplement: koag005_Supplementary_Data [file koag005_supplementary_data.zip › Supplementary Figure S7.png]

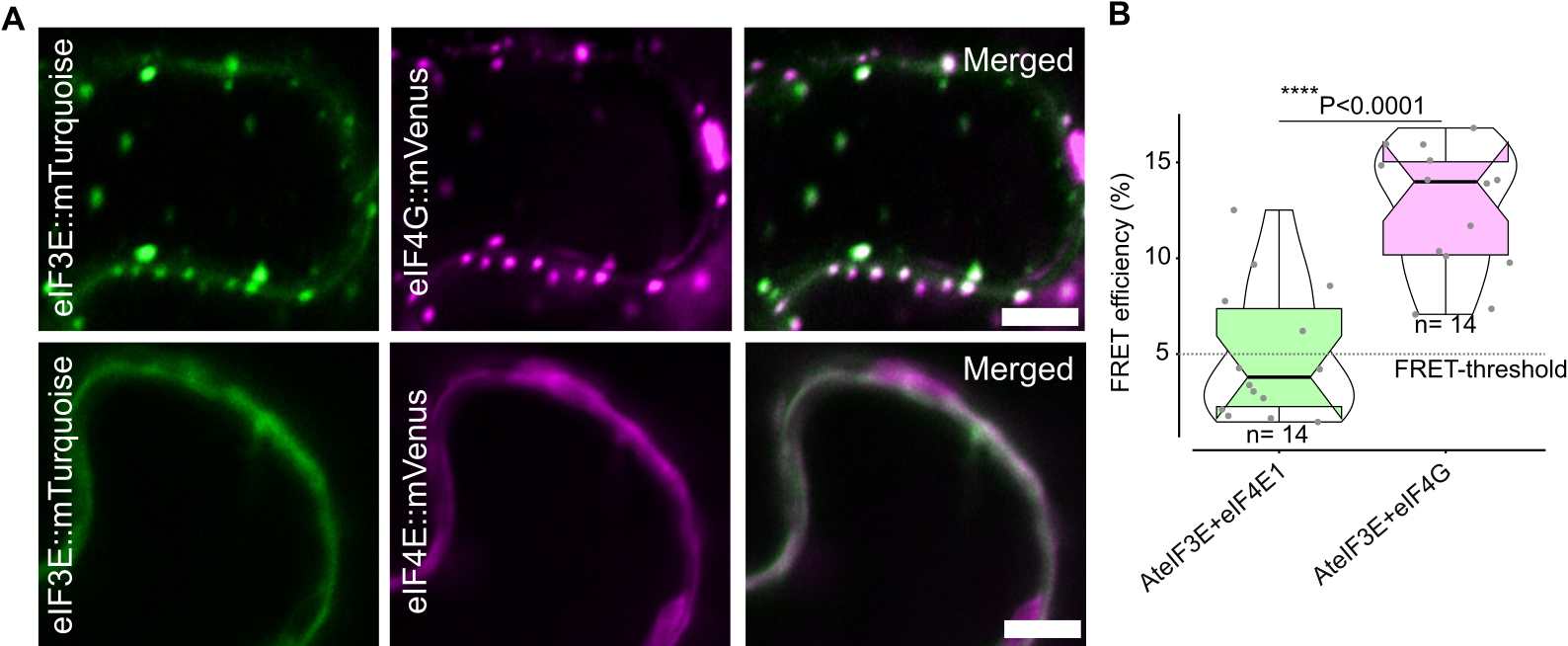

Supplement: koag005_Supplementary_Data [file koag005_supplementary_data.zip › Supplementary Figure S8.png]

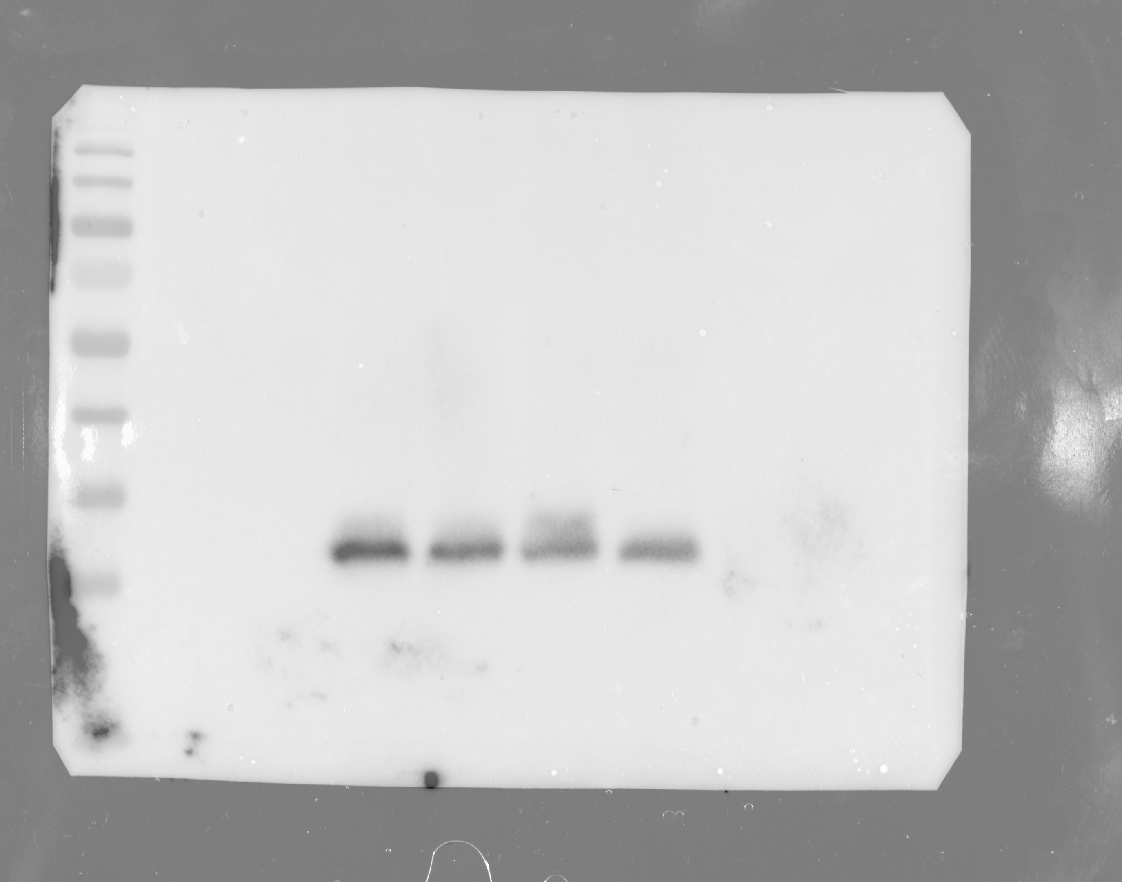

Supplement: koag005_Supplementary_Data [file koag005_supplementary_data.zip › Figure S7C_Raw.tif]

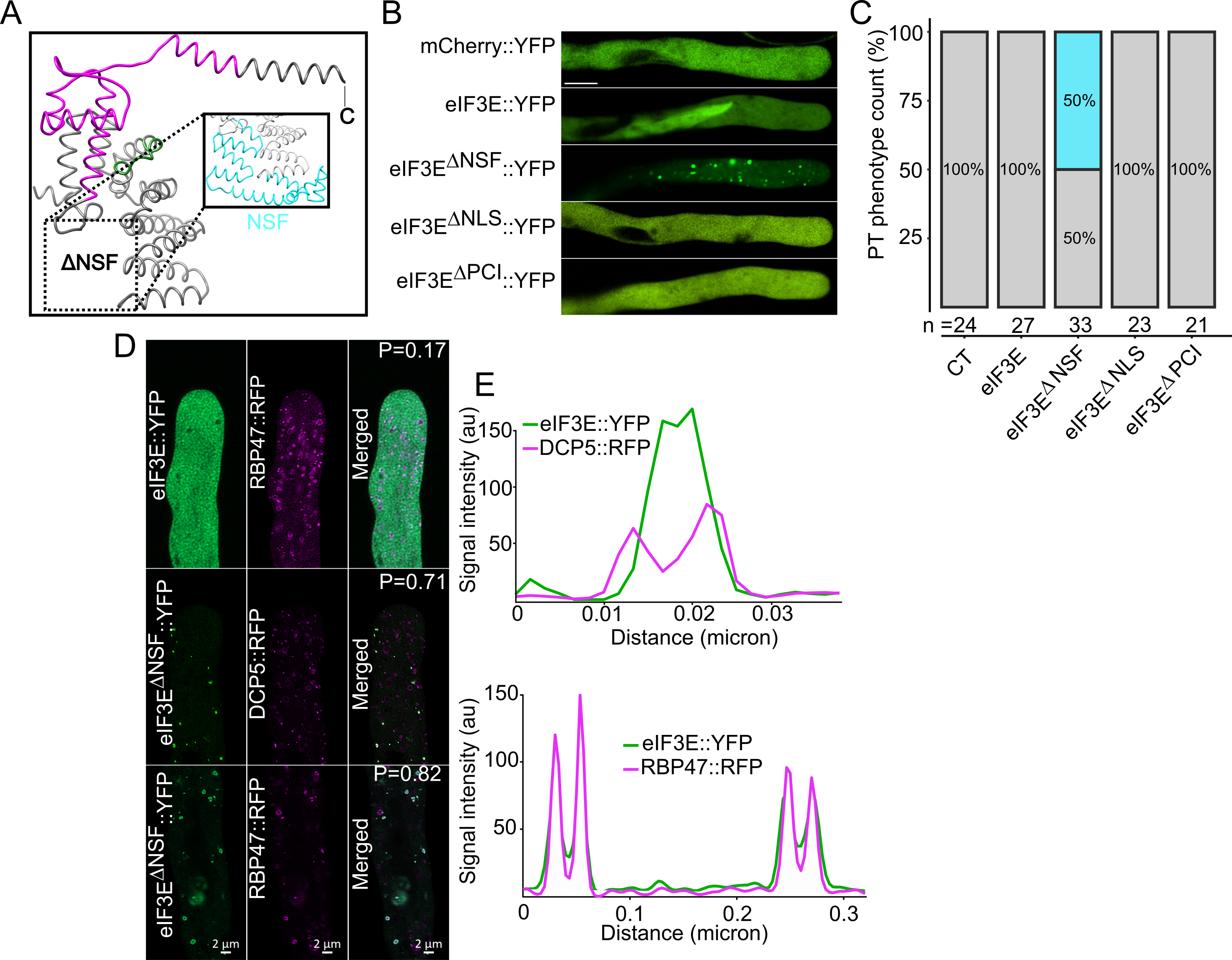

Supplement: koag005_Supplementary_Data [file koag005_supplementary_data.zip › Supplementary Figure S2.png]

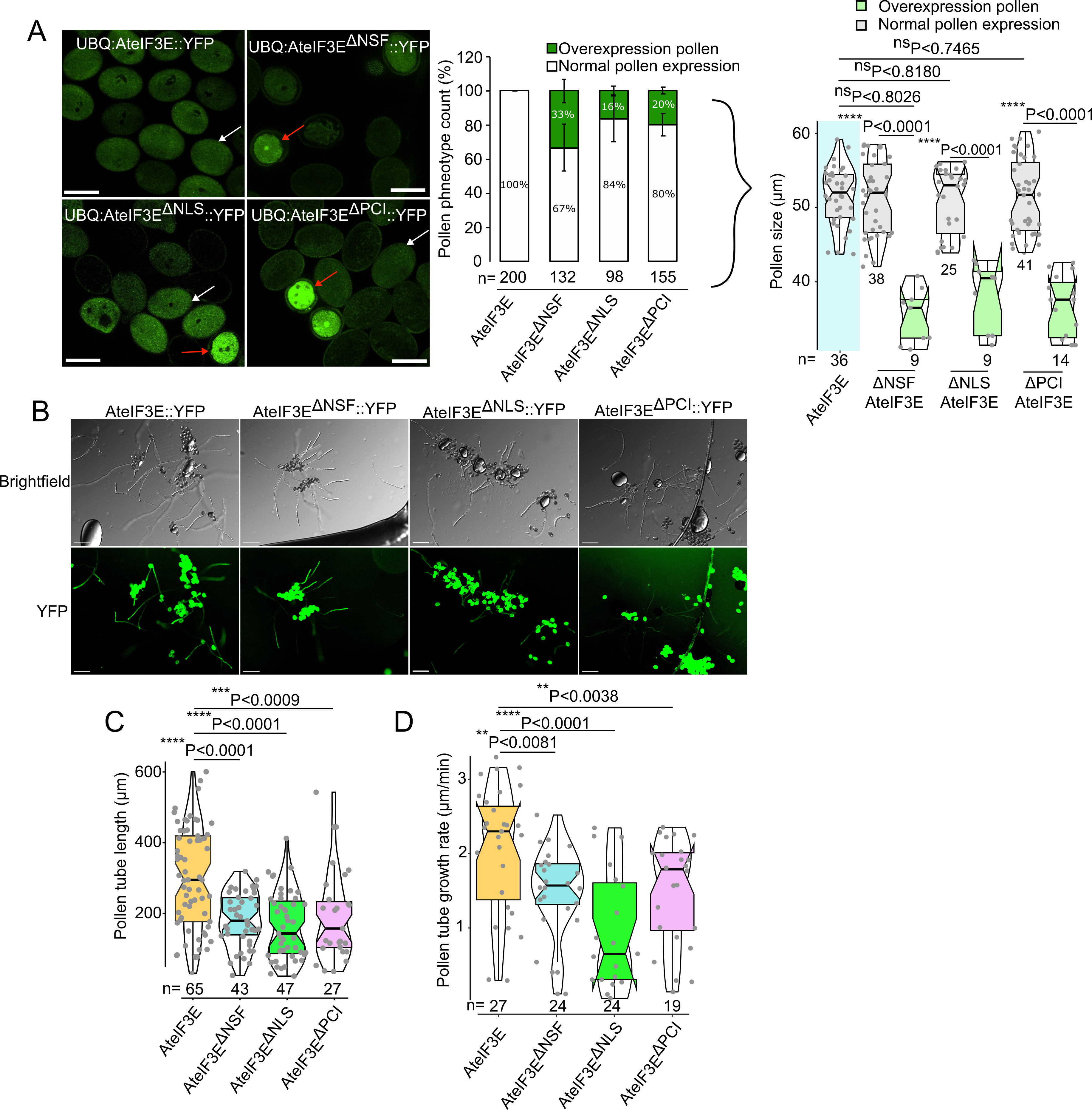

Supplement: koag005_Supplementary_Data [file koag005_supplementary_data.zip › Supplementary Figure S3.png]

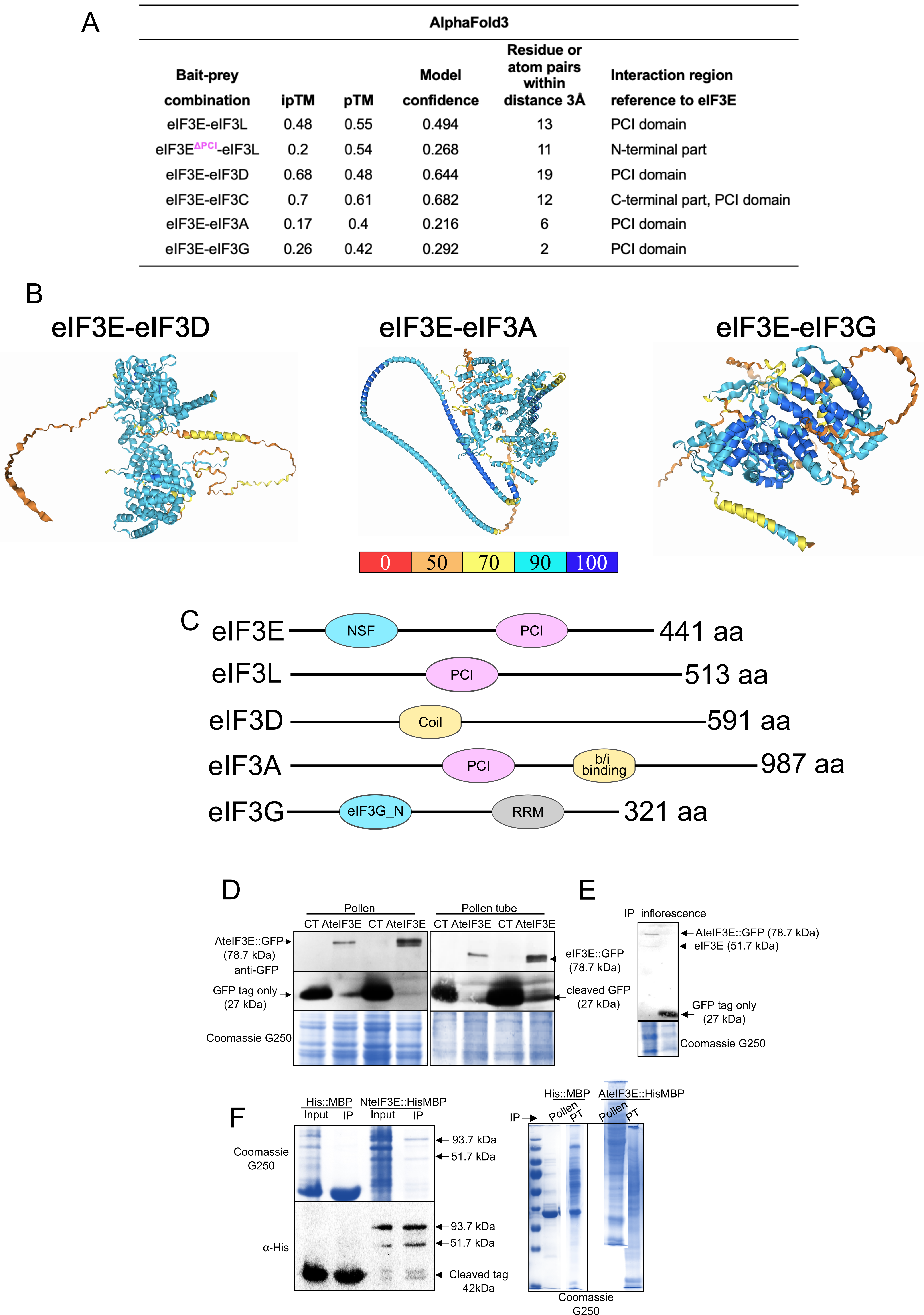

Supplement: koag005_Supplementary_Data [file koag005_supplementary_data.zip › Supplementary Figure S4.png]

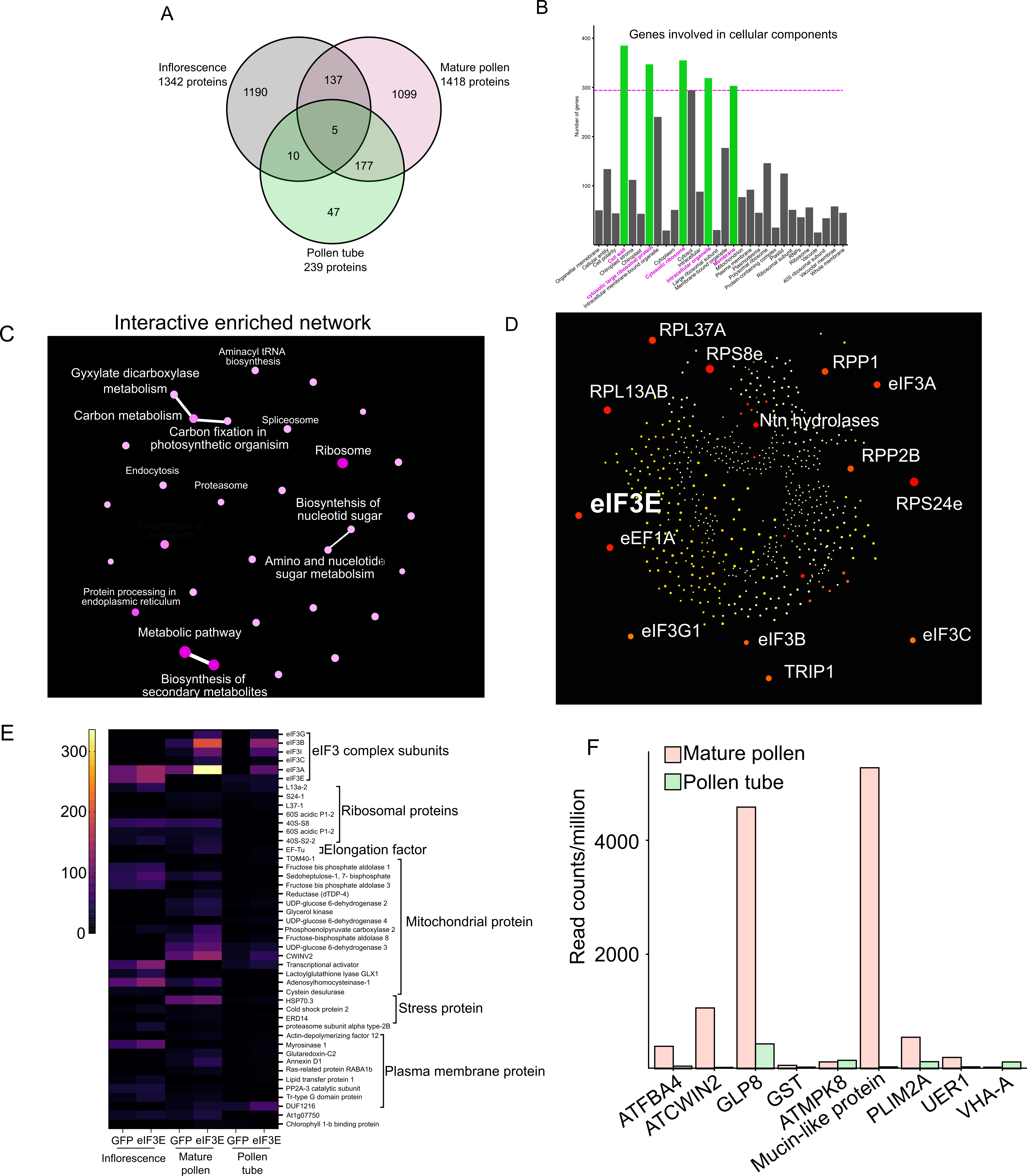

Supplement: koag005_Supplementary_Data [file koag005_supplementary_data.zip › Supplementary Figure S5.png]

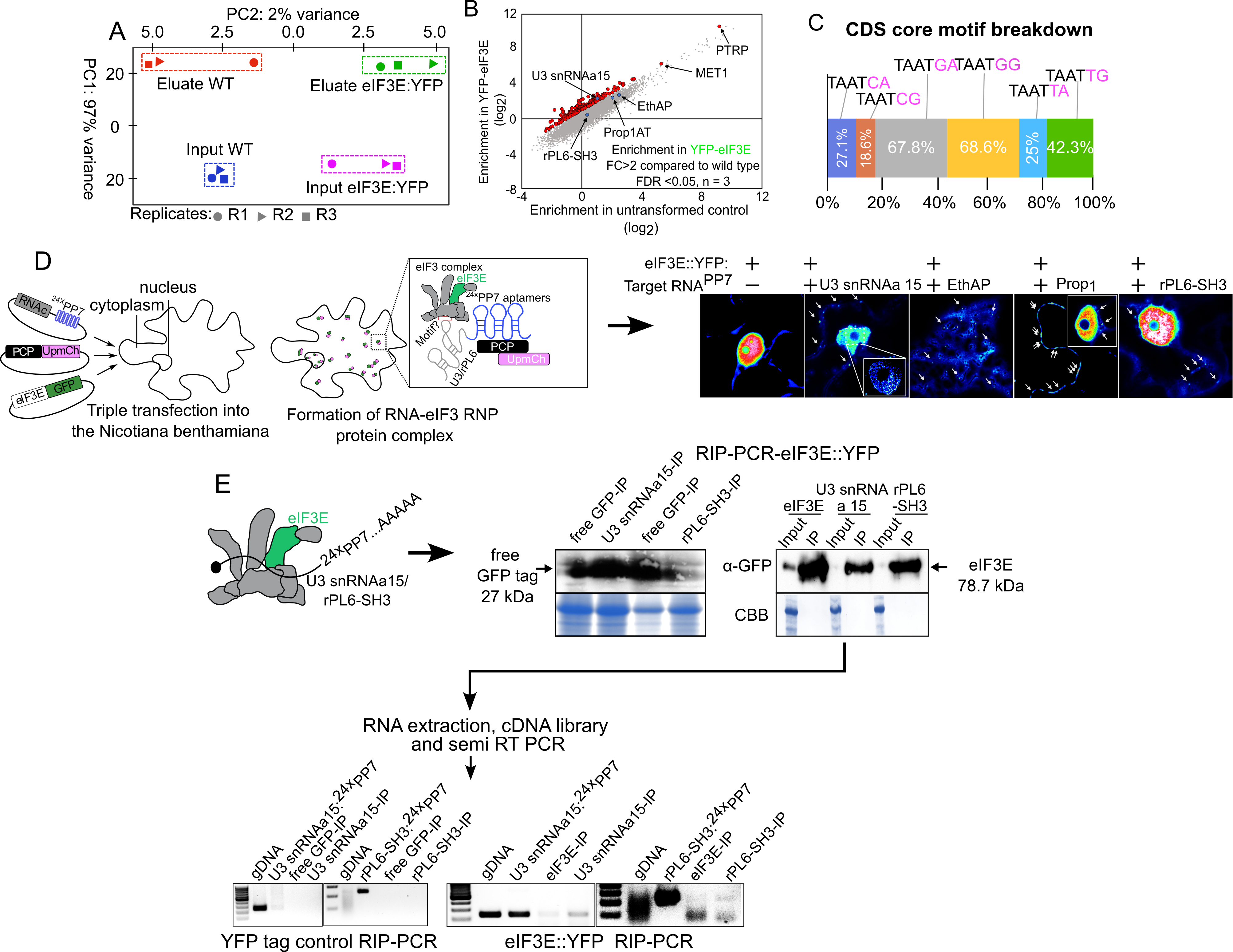

Supplement: koag005_Supplementary_Data [file koag005_supplementary_data.zip › Supplementary Figure S6.png]
